# Cyclizations and fragmentations in the alkylation of 6‐chloro‐5‐hydroxy‐4‐aminopyrimidines with aminoalkyl chlorides

**DOI:** 10.1002/jhet.4228

**Published:** 2021-02-10

**Authors:** Edwige M. H. Picazo, Amy B. Heptinstall, David M. Wilson, Céline Cano, Bernard T. Golding, Michael J. Waring

**Affiliations:** ^1^ Chemistry, School of Natural and Environmental Sciences Cancer Research UK Newcastle Drug Discovery Unit, Newcastle University Centre for Cancer, Newcastle University Newcastle upon Tyne UK; ^2^ Oncology Innovative Medicines Unit, AstraZeneca Cambridge UK; ^3^ Chemistry, School of Natural and Environmental Sciences Newcastle University Newcastle upon Tyne UK

## Abstract

Substituted aminopyrimidines are an important class of compounds, in part because they frequently show biological activity. Facile synthesis of polysubstituted aminopyrimidines is highly desirable for the synthesis of screening libraries. We describe a route to 4,6‐diamino‐5‐alkoxypyrimidines via a S_N_Ar‐alkylation‐S_N_Ar sequence from readily available 4,6‐dichloro‐5‐methoxypyrimidine, which allows the synthesis of such compounds with regiochemical control. The extension of this approach to alkylating agents bearing amino substituents led to unexpected and, in some cases, unprecedented products resulting from intramolecular S_N_Ar cyclization and subsequent fragmentation.

## Graphical abstract

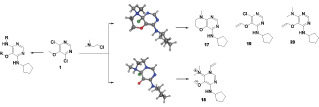

## 1 INTRODUCTION

**FIGURE 1:**
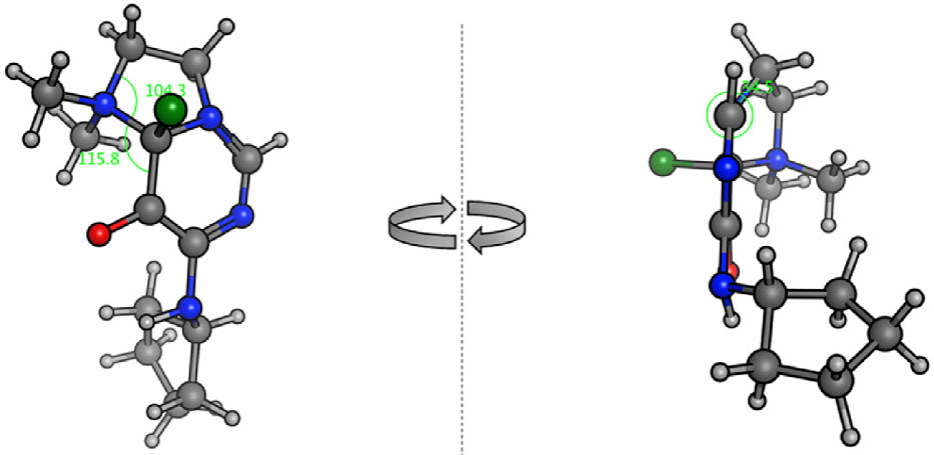
Energy minimized intermediate for the S_N_Ar cyclization leading to **9**

Pyrimidines can be regarded as privileged scaffolds that occur frequently in pharmaceuticals owing to their low lipophilicity and stability in vivo.^[^ ^1^ ^]^ Recently, amino‐substituted pyrimidines have found extensive application as kinase inhibitors in particular due to their ability to mimic the binding interactions of the adenosine moiety of ATP.^[^ ^2^ ^]^ As part of a program to prepare a library of adenosine mimics for biological screening, we required 4,6‐diaminopyrimidines further substituted at the 5‐position with an alkoxy group, in particular, those bearing basic functionality. The synthesis of *N*‐ and *O*‐substituted pyrimidines and related heterocycles is commonly achieved by displacement of a halide, or other leaving group from the heterocycle by S_N_Ar reaction;^[^ ^3^ ^]^ a metal‐mediated coupling (e.g. Buchwald‐Hartwig reaction)^[^ ^4^, ^5^ ^]^; or by alkylation of an amino or hydroxy substituent.^[^ ^6^ ^]^ In systems with multiple substituents, lack of control of regioselectivity often delivers a mixture of products. We envisaged that sequential S_N_Ar reactions of 4,6‐dichloro‐5‐methoxypyridine combined with methyl ether cleavage and *O*‐alkylation would afford the required templates (Figure 1). Consecutive S_N_Ar reactions of 4,6‐dichloro‐5‐alkoxypyrimidines with a wide range of amines to produce 4,6‐diamino‐5‐alkoxypyrimidines with alkyl or aryl substituents on the amino groups are extremely well precedented. Thus, ca. 1000 reports of the first S_N_Ar reaction and ca. 700 reports of the second with a variety of aliphatic and aromatic amines are in the current literature (Scifinder, accessed September 9, 2020).^[^ ^7^, ^8^, ^9^, ^10^ ^]^ However, to our knowledge, there are no reports of an intermediate *O*‐alkylation step. If achievable, this route would allow the introduction of diverse substituents selectively at each position from readily available amines and alkyl halides.

## 2 RESULTS AND DISCUSSION

**SCHEME 1:**
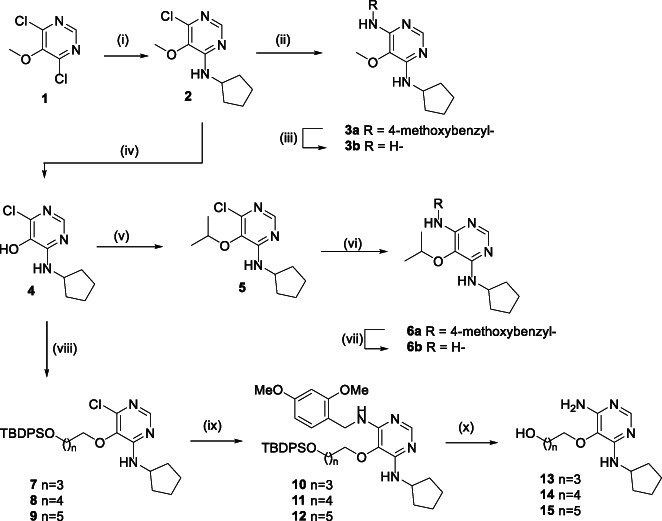
Reagents and conditions. (i) Cyclopentylamine, toluene, reflux, 93%; (ii) *p*‐methoxybenzylamine, DIPEA, dioxane, 220 °C, MW, 58%; (iii) TFA, DCM, reflux, 50%; (iv) BBr_3_, DCM, reflux, 98%; (v) 2‐bromopropane, K_2_CO_3_, MeCN / DMF, 80°C, 95%; (vi) *p*‐methoxybenzylamine, DIPEA, dioxane, 220°C, MW, 50%; (vii) TFA, DCM, reflux, 67%; (viii) RX, K_2_CO_3_, MeCN, DMF, 80 °C, n = 3 (RX = TBDPSO[CH_2_]_4_Cl), 73%, n = 4 (RX = TBDPSO[CH_2_]_5_Cl), 28%, n = 5, (RX = TBDPSO[CH_2_]_5_Br), 27%; (ix) 2,4‐dimethoxybenzylamine, DIPEA, dioxane, 220 °C, MW, n = 3, 33%, n = 4, 41%, n = 5, 54%; (x) TFA, DCM, reflux, n = 3, 38%, n = 4, 55%, n = 5, 61%

**SCHEME 2:**
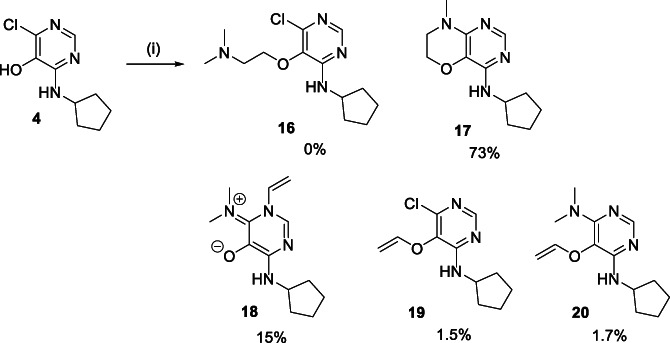
Reagents and conditions: (i) 2‐chloro‐N,N‐dimethylaminoethylamine hydrochloride, K_2_CO_3_, MeCN/DMF, 80°C, 20 hours

**SCHEME 3:**
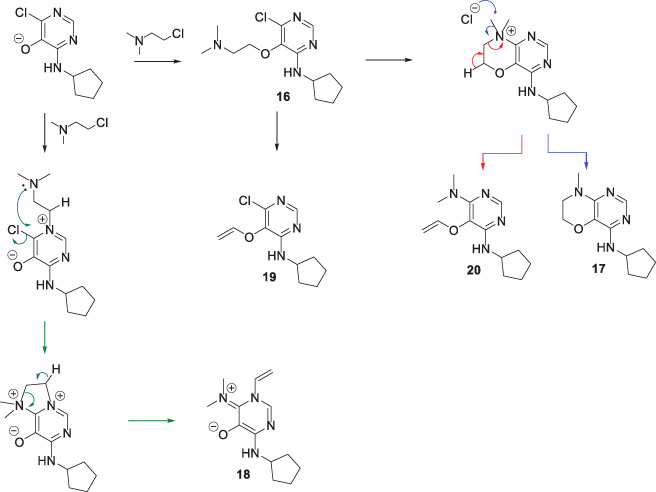
Proposed mechanism of formation of **17**, **18**, **19**, and **20**

**SCHEME 4:**
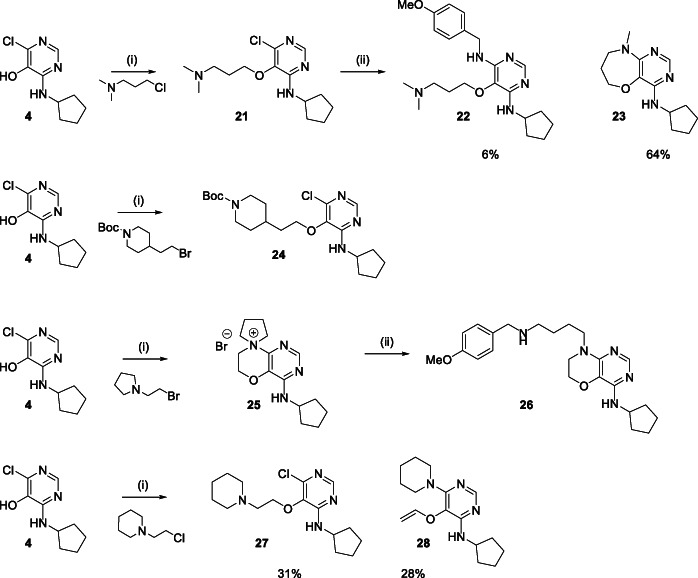
Reagents and conditions: (i) K_2_CO_3_, MeCN/DMF, 80°C, 3 hours; (ii) *p*‐methoxybenzylamine, DIPEA, dioxane, 220°C, MW

Introduction of the first amino‐substituent was easily achieved by the reaction of 4,6‐dichloro‐5‐methoxypyrimidine **1** with an amine, for example, cyclopentylamine, affording the monoamino derivative **2** in high yield (Scheme 1). A second S_N_Ar reaction with 4‐methoxybenzylamine under forcing conditions installed the 6‐amino‐substituted **3a** with moderate yield. In this case, the 4‐methoxybenzyl group could be removed with TFA to reveal the primary amine **3b**. Alternatively, BBr_3−_mediated cleavage of the 5‐methoxy group of **2** gave the 5‐hydroxy derivative **4** in high yield. Compound **4** was alkylated with 2‐bromopropane to afford the ether **5**, which also underwent S_N_Ar reaction with 4‐methoxybenzylamine to give 6‐amino derivatives in an analogous manner to **2**. Compound **4** could also be alkylated with a series of *O*‐silyloxy substituted alkyl halides to afford the silylether derivatives **7**–**9**, which underwent analogous S_N_Ar reactions with 2,4‐dimethoxybenzylamine to give the amino derivatives **10**–**12** in moderate to good yields. TFA‐mediated removal of both the 2,4‐dimethyoxybenzyl and TBDPS groups afforded the library compounds **13**–**15**, which contained hydroxyl groups at varying distances from the pyrimidine ring. These illustrative examples, coupled with the extensive precedent for the S_N_Ar steps show that the sequence provides an efficient route to a library of 4,5,6‐trisubstituted pyrimidines with diverse substituents. Alkylation of **4** with amino‐substituted alkyl halides proved more complicated. Treatment of **4** with 2‐dimethylaminoethyl chloride resulted in a mixture of products, none of which corresponded to the desired 5‐(2‐dimethylaminoethoxy)pyrimidine **16** (Scheme 2). The products consisted of the *N*‐methylmorpholine fused derivative **17** as the major component (73%). The ^1^H NMR spectrum of a second product formed in appreciable quantity indicated the presence of a vinyl group attached to a heteroatom [δ 5.32 (1H, dd, *J* = 8.1 and 3.9 Hz), 5.47 (1H, dd, *J* = 15.4 and 3.9 Hz), 6.98 (1H, dd, *J* = 15.4 and 8.1 Hz] and the absence of a neutral pyrimidine ring system [δ 7.47 (1H, s)]. The structure was thus ascribed to the zwitterion 6‐imino‐1‐vinyl‐1,6‐dihydropyrimidin‐5‐olate **18**. The mass spectrum, DEPT, and 2D NMR studies were consistent with this structure (see Supporting Information). Two minor products were also isolated from the reaction, which were assigned from mass and NMR spectra to the 6‐chloro‐1‐vinylpyrimidinium‐5‐olate **19** and the 6‐dimethylamino‐5‐(vinyloxy)pyrimidine **20** (see Supporting Information). Shortening the reaction time from 20 to 2 hours, although not proceeding to full conversion, allowed the ether **16** to be isolated (53% yield) with only minor amounts of **17** and **20** observed. The formation of these products can be explained by initial competing *O*‐ versus *N* ^1^‐alkylation of the anion of hydroxypyridine **4** (Scheme 3), with *O*‐alkylation favored by approximately 5:1. Intramolecular S_N_Ar reaction of the pendant dimethylamino‐moiety of **16** affords a fused morpholinium species, which can undergo chloride mediated demethylation (blue arrows) to form the morpholine **17** as the major product. Alternatively, elimination of the ammonium group (red arrows) leads to the vinyl ether **20**. Compound **16** can also eliminate the dimethylamino‐ group, possibly promoted by quarternization of the amine with excess of alkylating agent, to give vinyl ether **19**. The *N* ^1^‐alkylated product can undergo a similar cyclization by S_N_Ar displacement of the chloride by the dimethylamino group (green arrows) followed by elimination of the ammonium group leading to the iminodihydropyrimidinolate **18**. The initial S_N_Ar cyclization would, in this case, proceed via a strained transition state to accommodate an angle of attack of the amine on the aryl system with a trajectory close to the Burgi‐Dunitz angle.^[^ ^11^ ^]^ An energy minimized model of the resulting intermediate (Figure 1) suggests that such a reaction is feasible. To accommodate the incipient ring requires a distortion of the dihedral angle at *N* ^1^ of 34.5°, with tetrahedral angle at the dimethylammonium nitrogen of 104° internal to the 5‐membered ring and 116° external between the C—N bond and the pyrimidine ring. Corresponding angles in an equivalent minimized structure with the N^1^ dihedral constrained to 0° are 104° and 128°, respectively (not shown). Analogous cyclization of 3‐aminoethyl‐2‐chloropyridines have been reported.^[^ ^12^, ^13^, ^14^ ^]^ Subsequent elimination to form **18** requires removal of the proton from the CH *anti* to the C‐N bond undergoing cleavage, facilitated by relief of ring‐strain and quenching of the positive charge on the amine. Alkylation of **4** with 3‐dimethylamino‐1‐propyl chloride gave the desired 5‐(3‐dimethylaminopropoxy)pyrimidine **21** in 70% yield (Scheme 4). Heating **21** with 4‐methoxybenzylamine formed **22** to a minor degree, but led to the cyclized, demethylated tetrahydrooxazepine **23** as the major product (64% yield). Hence, an intramolecular S_N_Ar reaction occurs in preference to intermolecular S_N_Ar displacement to produce **22**. As anticipated, the process leading to a 7‐membered system must occur less readily than that giving the 6‐membered system because **23** was only observed under the subsequent S_N_Ar reaction conditions, in contrast to the formation of **17** in the initial alkylation reaction. Alkylation of **4** with 4‐(2‐chloroethyl)‐N‐Boc‐piperidine proceeded in 78% yield to the ether **24** with no observed side‐products, showing that the side reactions can be prevented by masking the amino group. Treatment of **4** with N‐(2‐bromoethyl)pyrrolidine resulted in the 6,7‐dihydrospiro[pyrimido[5,4‐b][1,4]oxazine‐8,1′‐pyrrolidin]‐8‐ium **25** (isolated as the chloride salt after workup including aqueous ammonium chloride wash), in which the intramolecular S_N_Ar occurred subsequent to *O*‐alkylation to form the morpholinium motif. The presence of a spirocyclic morpholine system fused to the pyrimidine ring was established by NMR (see Supporting Information). The presence of the pyrrolidine system renders the system more stable to further dealkylation and fragmentation processes as no other side‐products were observed in this case. Treatment of compound **25** with 4‐methoxybenzylamine resulted in the formation of *N*‐alkylated morpholine **26**, arising from nucleophilic attack on the pyrrolidine ring. In contrast, a similar procedure with the homologous piperidine resulted in a 1:1 mixture of the ether **27** and vinyl ether **28** with a morpholinium species not being observed. This result indicates that with the piperidine, cyclization occurs less readily, while elimination is more favorable, compared to the pyrrolidine. These observations could be attributed to a stereoelectronic impediment to elimination with the spiro species **25**, compared to the piperidine case. The formation of morpholine **26** by nucleophilic ring opening of morpholinium **25** implies a more general route to pyrimidine‐fused *N*‐substituted morpholines. This chemistry could be expanded further if alternative cyclized species such as the piperidine precursor to **28** could be intercepted in situ prior to elimination. The synthetic sequences developed in this work provide an efficient means of preparing 4,5,6‐trisubstituted pyrimidine derivatives via an S_N_Ar‐alkylation‐S_N_Ar sequence, leading to compounds with a diverse range of substituents, of utility for biological screening. This system has been shown to be problematic for the introduction of 5‐amino containing analogs in systems for which an intramolecular cyclization can occur, and results in novel fragmentation and cyclization processes forming a range of products, the exact nature of which depends on the structure, including the highly unusual 6‐imino‐1‐vinyl‐1,6‐dihydropyrimidin‐5‐olate **18**, a formally zwitterionic system that has not been previously described to our knowledge. As well as presenting reactions to be aware of with similar systems, our observations provide insights into the reactivity of chloropyrimidines bearing pendant amines and their cyclized derivatives.

## 3 EXPERIMENTAL SECTION

### 3.1 4‐Chloro‐6‐(cyclopentylamino)pyrimidin‐5‐ol **4**

To a solution of **2** (640.0 mg, 2.81 mmol) in anhydrous DCM (2.5 mL) was added a solution of boron tribromide 1.0M in DCM (14 mL, 14 mmol) drop‐wise over a 30 min period. The reaction mixture was stirred at room temperature for 15 min and then heated at 40°C for 15 hours, cooled to 0°C, and slowly quenched with water (violent reaction). The pH was brought to pH = 6 with sat. aq. NaHCO_3_. The aqueous layer was extracted with DCM (3 × 30 mL). The combined organic layers were dried (MgSO_4_), filtered, and concentrated in vacuo to afford the title compound as a light brown powder (588.0 mg, 2.75 mmol, 97%) which was carried forward without purification. *R*_f_ = 0.43 (50% EtOAc in 40–60 petrol); mp = 140.3–148.1 °C; UV λ_max_ (EtOH) nm: 255; IR ν_max_ cm^−1^: 3413, 3368 (NH), 2947, 2863, 2639, 2516, 2113, 1568, 1506, 1417, 1342, 1225, 1148, 1115, 1054; ^1^H‐NMR (500 MHz, MeOD) δ_H_ 1.52–1.59 (2H, m, CH_2_‐C*H*_2_‐CH*‐cyclopentane*), 1.61–1.68 (2H, m, C*H*_2_ *‐cyclopentane*), 1.74–1.82 (2H, m, C*H*_2_ *‐cyclopentane*), 2.02–2.08 (2H, m, CH_2_‐C*H*_2_‐CH*‐cyclopentane*), 4.35 (1H, quin, *J* = 7.0 Hz, C*H‐cyclopentane*), 7.85 (1H, s, *H‐pyrimidine*), NH and OH not visible; ^13^C‐NMR (125 MHz, MeOD) δ_C_ 24.7 (*C*H_2_ *‐cyclopentane* and *C*H_2_ *‐cyclopentane*), 33.6 (CH_2_‐*C*H_2_‐CH‐*C*H_2_ *‐cyclopentane*), 53.9 (*C*H*‐cyclopentane*), 134.4 (*C*‐O), 139.6 (*C*‐Cl), 149.9 (*C*H*‐pyrimidine*), 156.5 (*C*‐N); MS (ES^+^) *m/z* 214.1 [M(^35^Cl) + H]^+^ and *m/z* 216.1 [M(^37^Cl) + H]^+^, (ES^−^) *m/z* 212.0 [M(^35^Cl) + H]^−^ and *m/z* 214.0 [M(^37^Cl) + H]^−^.

### 3.2 N‐Cyclopentyl‐8‐methyl‐7,8‐dihydro‐6H‐pyrimido[5,4‐b][1,4]oxazin‐4‐amine 17, 4‐(cyclopentylamino)‐6‐(dimethyliminio)‐1‐vinyl‐1,6‐dihydropyrimidin‐5‐olate 18, 6‐chloro‐N‐cyclopentyl‐5‐(vinyloxy)pyrimidin‐4‐amine 19, N^4^‐cyclopentyl‐N^6^ ,N^6^ ‐dimethyl‐5‐(vinyloxy)pyrimidine‐4,6‐diamine **20**

To a stirred suspension of **4** (240.0 mg, 1.40 mmol) and K_2_CO_3_ (582.0 mg, 4.20 mmol) in MeCN (15 mL) and DMF (6 mL) was added 2‐chloro‐N,N‐dimethylamine hydrochloride (302.0 mg, 2.10 mmol). The reaction mixture was heated at 80 °C overnight, washed with water and sat. aq. NH_4_Cl (10 mL), extracted with DCM (3 × 10 mL), dried (MgSO_4_), filtered and concentrated in vacuo. The crude product was purified by flash chromatography (silica‐gel column, 0–40% EtOAc in 40–60 petrol, then 100% MeOH) to afford: **17** as a light brown oil (240 mg, 1.02 mmol, 73%). R_f_ = 0.65 (10% MeOH in DCM); UV λ_max_ (EtOH) nm: 228; IR ν_max_ cm^−1^: 3427, 2945, 2863, 1596, 1515, 1477, 1445, 1406, 1372, 1339; ^1^H‐NMR (500 MHz, CDCl_3_) δ_H_ 1.37–1.43 (2H, m, CH_2_‐C*H*_2_‐CH*‐cyclopentane*), 1.55–1.74 (4H, m, C*H*_2_ *‐cyclopentane*), 1.98–2.06 (2H, m, CH_2_‐C*H*_2_‐CH*‐cyclopentane*), 3.04 (3H, s, N‐C*H*_*3*_), 3.37 (2H, t, *J* = 4.5 Hz, C*H*_*2*_‐N), 4.17 (2H, t, *J* = 4.5 Hz, C*H*_*2*_‐O), 4.30 (1H, sex, *J* = 6.7 Hz, C*H‐cyclopentane*), 4.56 (1H, d, *J* = 6.7 Hz, N*H*), 7.93 (1H, s, *H‐pyrimidine*); ^13^C‐NMR (125 MHz, CDCl_3_) δ_C_ 23.6 (*C*H_2_ *‐cyclopentane* and *C*H_2_ *‐cyclopentane*), 33.7 (CH_2_‐*C*H_2_‐CH‐*C*H_2_ *‐cyclopentane*), 35.2 (N‐*C*H_3_), 48.2 (*C*H_2_‐N), 52.3 (*C*H*‐cyclopentane*), 63.7 (*C*H_2_‐O), 120.4 (*C*‐O), 148.3 (*C*‐N), 150.1 (*C*H*‐pyrimidine*), 150.2 (*C*‐N); MS (ES^+^) *m/z* 235.2 [M + H]^+^; **18** as a dark brown oil (54.0 mg, 0.22 mmol, 15%). UV λ_max_ (EtOH) nm: 226; IR ν_max_ cm^−1^: 3339, 2953, 2868, 1605, 1548, 1518, 1447, 1396, 1334; ^1^H‐NMR (500 MHz, CDCl_3_) δ_H_ 1.44–1.52 (2H, m, CH_2_‐C*H*_2_‐CH*‐cyclopentane*), 1.53–1.74 (4H, m, C*H*_2_ *‐cyclopentane*), 1.96–2.04 (2H, m, CH_2_‐C*H*_2_‐CH*‐cyclopentane*), 3.65 (6H, s, N[C*H*_*3*_]_*2*_), 4.26 (1H, sex, *J* = 6.9 Hz, C*H‐cyclopentane*), 5.32 (1H, dd, *J* = 8.1 and 3.9 Hz, H_b_), 5.47 (1H, dd, *J* = 15.4 and 3.9 Hz, H_c_), 6.27 (1H, d, *J* = 6.9 Hz, N*H*), 6.98 (1H, dd, *J* = 15.4 and 8.1 Hz, N‐C*H*_*a*_ = CH_2_), 7.47 (1H, s, *H‐pyrimidine*); ^13^C‐NMR (125 MHz, CDCl_3_) δ_C_ 23.7 (*C*H_2_ *‐cyclopentane* and *C*H_2_ *‐cyclopentane*), 33.2 (CH_2_‐*C*H_2_‐CH‐*C*H_2_ *‐cyclopentane*), 51.1 (N[*C*H_3_]_2_), 52.3 (*C*H*‐cyclopentane*), 108.7 (*C*H_2_‐*vinyl*), 136.1 (*C*‐O), 136.4 (*C*H*‐pyrimidine*), 142.1 (*C*H‐*vinyl*), 142.6 (*C*‐N), 161.9 (*C*‐NH); MS (ES^+^) *m/z* 249.3 [M + H]^+^; **19** as a light brown oil (5.0 mg, 0.021 mmol, 1%). UV λ_max_ (EtOH) nm: 249; IR ν_max_ cm^−1^: 3427, 3279, 2954, 2868, 1627, 1569, 1498, 1409, 1337; ^1^H‐NMR (500 MHz, CDCl_3_) δ_H_ 1.44–1.49 (2H, m, CH_2_‐C*H*_2_‐CH*‐cyclopentane*), 1.56–1.76 (4H, m, C*H*_2_ *‐cyclopentane*), 2.04–2.12 (2H, m, CH_2_‐C*H*_2_‐CH*‐cyclopentane*), 4.34–4.41 (1H, m, C*H‐cyclopentane*), 4.40 (1H, dd, *J* = 6.3 and 2.9 Hz, H_b_), 4.46 (1H, dd, *J* = 13.9 and 2.9 Hz, H_c_), 5.04–5.13 (1H, br m, N*H*), 6.46 (1H, dd, *J* = 13.9 and 6.3 Hz, O‐C*H*_*a*_ = CH_2_), 8.22 (1H, s, *H‐pyrimidine*); ^13^C‐NMR (125 MHz, CDCl_3_) δ_C_ 23.6 (*C*H_2_ *‐cyclopentane* and *C*H_2_ *‐cyclopentane*), 33.2 (CH_2_‐*C*H_2_‐CH‐*C*H_2_ *‐cyclopentane*), 52.9 (*C*H*‐cyclopentane*), 92.7 (*C*H_2_‐*vinyl*), 130.4 (*C*‐O), 148.1 (*C*H‐*vinyl*), 149.0 (*C*‐Cl) 154.2 (*C*H*‐pyrimidine*), 156.6 (*C*‐N); MS (ES^+^) *m/z* 240.2 [M(^35^Cl) + H]^+^ and *m/z* 242.2 [M(^37^Cl) + H]^+^; **20** as a brown oil (6.0 mg, 0.024 mmol, 1.7%). UV λ_max_ (EtOH) nm: 236; IR ν_max_ cm^−1^: 3436, 2950, 2866, 1583, 1486, 1412, 1326; ^1^H‐NMR (500 MHz, CDCl_3_) δ_H_ 1.37–1.43 (2H, m, CH_2_‐C*H*_2_‐CH*‐cyclopentane*), 1.55–1.74 (4H, m, C*H*_2_ *‐cyclopentane*), 1.98–2.06 (2H, m, CH_2_‐C*H*_2_‐CH*‐cyclopentane*), 3.07 (6H, s, N[C*H*_*3*_]_*2*_), 4.27 (1H, dd, *J* = 6.4 and 2.3 Hz, H_b_), 4.31 (1H, sex, *J* = 6.9 Hz, C*H‐cyclopentane*), 4.41 (1H, dd, *J* = 13.9 and 2.3 Hz, H_c_), 4.65 (1H, d, *J* = 6.9 Hz, N*H*), 6.31 (1H, dd, *J* = 13.9 and 6.4 Hz, O‐C*H*_*a*_ = CH_2_), 8.07 (1H, s, *H‐pyrimidine*); ^13^C‐NMR (125 MHz, CDCl_3_) δ_C_ 23.6 (*C*H_2_ *‐cyclopentane* z *C*H_2_ *‐cyclopentane*), 33.6 (CH_2_‐*C*H_2_‐CH‐*C*H_2_ *‐cyclopentane*), 39.2 (N[*C*H_3_]_2_), 48.2 (*C*H_2_‐N), 52.5 (*C*H*‐cyclopentane*), 92.0 (*C*H_2_‐*vinyl*), 118.9 (*C*‐O), 150.0 (*C*H‐*vinyl*), 153.3 (*C*‐N), 154.7 (*C*H*‐pyrimidine*), 156.1 (*C*‐N); MS (ES^+^) *m/z* 249.3 [M + H]^+^.

## Supporting information


**Appendix S1**. Supporting information

## Data Availability

All data supporting the manuscript are included in the submission, including full experimental data in the supporting information.
