# Supplementary material for: Cyclizations and fragmentations in the alkylation of 6‐chloro‐5‐hydroxy‐4‐aminopyrimidines with aminoalkyl chlorides
Source: J Heterocycl Chem. 2021 Feb 10;58(4):947–51. doi: 10.1002/jhet.4228 (PMC8600947; doi:10.1002/jhet.4228)

Cyclisations and fragmentations in the alkylation of 6-chloro-5-hydroxy-4-aminopyrimidines with aminoalkyl chlorides

Edwige M. H. Picazo,^a^ Amy B. Heptinstall,^a^ David M. Wilson,^b^ Céline Cano,^a^ Bernard T. Golding^c^ and Michael J. Waring^a^

^a^ Cancer Research UK Newcastle Drug Discovery Unit, Newcastle University Centre for Cancer, Chemistry, School of Natural and Environmental Sciences, Bedson Building, Newcastle University, Newcastle upon Tyne, NE1 7RU, UK.

Email: mike.waring@ncl.ac.uk

^b^ Oncology Innovative Medicines Unit, AstraZeneca, 310 Milton Rd. Cambridge CB4 0WG, UK.

^c^ Chemistry, School of Natural and Environmental Sciences, Bedson Building, Newcastle University, Newcastle upon Tyne, NE1 7RU, UK.

**Supplementary Material**

**Content**

1. Molecular modelling
2. General information
3. Experimental procedures
4. NMR spectra
5. **Molecular modelling**

Molecular modelling was carried out in Molecular Operating Environment (MOE) v. 2019.0102 (Chemical Computing Group, [www.chemcomp.com](http://www.chemcomp.com/)). Energy minimisation was carried out using the Amber10:EHT forcefield with a gradient of 0.1 RMS kcal/mol/Å^2^.

1. **General information**

Chemicals were purchased from reputable suppliers, and used without further purification. Anhydrous solvents were purchased from Sigma-Aldrich or Acros in SureSeal™ or Acroseal™ bottles, respectively.

TLC was performed on Merck silica gel 60 F254 plates, or Merck amino-modified NH_2_ silica plates where appropriate. Visualization was achieved with 254 nm UV light where possible or by staining with iodine, ninhydrin, phosphomolybdic acid, potassium permanganate, or vanillin. Purifications were carried out by flash column chromatography using a Biotage Isolera automated system with UV-directed collection between 200 nm and 400 nm, or a Biotage SP4 automated flash system with UV monitoring at 298 nm and collection at 254 nm. Grace Resolv pre-packed flash cartridges were used for normal phase separations.

FTIR spectra were recorded neat with an Agilent Cary 630 FTIR spectrometer with an internal referencing. UV spectra were recorded in ethanol on a Hitachi U-2800A spectrophotometer. LC-MS analyses were conducted using a Waters Acquity UPLC system with PDA and ELSD.

^1^H NMR spectra were obtained using an internal deuterium lock at ambient probe temperatures (unless otherwise stated) on a Bruker Avance III 500 spectrometer using a frequency of 500 MHz or a Bruker Avance III 300 spectrometer using a frequency of 300 MHz. ^13^C spectra were acquired using broadband proton spin decoupling at ambient probe temperature (unless otherwise stated) using an internal deuterium lock on the Bruker Avance III 500 spectrometer operating at a frequency of 125 MHz. Chemical shifts (δ_H_ and δ_C_) are reported in ppm to the nearest 0.01 ppm and coupling constants are reported in Hz to the nearest 0.1 Hz. Deuterated solvents are referenced at 7.26 ppm (^1^H) and 77.2 ppm (^13^C) for CDCl3, 3.31 ppm (^1^H) and 49.0 ppm (^13^C) for MeOD, 2.50 ppm (^1^H) and 39.5 ppm (^13^C) for DMSO-d6. Data are reported in the format: chemical shift, integration, multiplicity, coupling constant(s) and assignment. The abbreviations for spin multiplicity are quoted as follows: s = singlet; d = doublet; t = triplet; q = quartet, quin = quintet, sex = sextet, sept = septet and m = multiplet. Combinations of these abbreviations are employed to describe more complex splitting patterns (e.g. dd = doublet of doublets) and where broadening of the peak is observed, spin multiplicity is accompanied by the prefix br = broad. Assignments were determined either by unambiguous chemical shift coupling patterns, by patterns observed in two-dimensional experiments (^1^H-^1^H COSY, HMBC or HSQC) or by analogy to fully interpreted spectra for related compounds.

1. **Experimental procedures**

6-chloro-*N*-cyclopentyl-5-methoxypyrimidin-4-amine **2**

To a stirred solution of 4,6-dichloromethoxypyrimidine **1** (0.200 g, 1.12 mmol) in anhydrous toluene (5.6 mL) was added cyclopentylamine (0.2 mL, 2.24 mmol). The reaction mixture was heated at 100 °C for 16 h. Upon cooling the reaction mixture was diluted with DCM (10 mL), washed with water and sat. aq. NH_4_Cl (2x 10 mL). The organic layers were dried (MgSO_4_), filtered and concentrated *in vacuo* to afford the title compound as a yellow oil (238.0 mg, 1.05 mmol, 93%) which was carried forward without purification. R_f_ = 0.26 (25% EtOAc in 40-60 petrol). UV λ_max_ (EtOH) nm: 251; IR ν_max_ cm^-1^: 3308 (NH), 2951, 2839, 2117, 1641, 1575, 1495, 1448, 1397, 1338, 1249, 1015; ^1^H-NMR (500 MHz, CDCl_3_) δ_H_ 1.43-1.50 (2H, m, CH_2_-C*H*_2_-CH*-cyclopentane*), 1.60-1.78 (4H, m, C*H*_2_*-cyclopentane* and C*H*_2_*-cyclopentane*), 2.06-2.13 (2H, m, CH_2_-C*H*_2_-CH*-cyclopentane*), 3.85 (3H, s, C*H_3_*-O), 4.35 (1H, quin, *J* = 7.2 Hz, C*H-cyclopentane*), 5.33 (1H, br s , N*H*), 8.14 (1H, s, *H-pyrimidine*); ^13^C-NMR (125 MHz, CDCl_3_) δ_C_ 23.8 (*C*H_2_*-cyclopentane* and *C*H_2_*-cyclopentane*), 33.4 (CH_2_-*C*H_2_-CH-*C*H_2_*-cyclopentane*), 52.8 (*C*H*-cyclopentane*), 60.1 (O-*C*H_3_), 135.5 (*C*-O), 147.8 (*C*-Cl), 152.3 (*C*H*-pyrimidine*), 157.5 (*C*-N); MS (ES^+^) *m/z* 228.0 [M(^35^Cl)+H]^+^ and *m/z* 230.1 [M(^37^Cl)+H]^+^.

*N*^4^-cyclopentyl-5-methoxy-*N*^6^-(4-methoxybenzyl)pyrimidine-4,6-diamine **3a**

To a solution of **2** (50.0 mg, 0.22 mmol) in anhydrous dioxane (1 mL) in a microwave vial were added p-methoxybenzylamine (0.1 mL, 0.66 mmol) and DIPEA (0.07 mL, 0.44 mmol). The resulting mixture was heated at 180 °C for 0.5 h and then 220 °C for 2 h under microwave irradiation. The reaction mixture was diluted with DCM (10 mL) and washed with sat. aq. NH_4_Cl (10 mL). The aqueous layer was extracted with DCM (3 × 10 mL). The combined organic layers were dried (MgSO_4_), filtered and concentrated *in vacuo*. The crude product was purified by flash chromatography (silica-gel column, 0-5% MeOH in DCM) to afford the title compound as a pale oil (41.0 mg, 0.12 mmol, 57%). R_f_ = 0.64 (5% MeOH in DCM); UV λ_max_ (EtOH) nm: 228; IR ν_max_ cm^-1^: 3328 (NH), 3201, 2951, 2865, 2111, 1674, 1591, 1508, 1316, 1243, 1173, 1136; ^1^H-NMR (500 MHz, CDCl_3_) δ_H_ 1.40-1.47 (2H, m, CH_2_-C*H*_2_-CH*-cyclopentane*), 1.57-1.61 (1H, br s, N*H*), 1.61-1.76 (4H, m, C*H*_2_*-cyclopentane* and C*H*_2_*-cyclopentane*), 2.03-2.11 (2H, m, CH_2_-C*H*_2_-CH*-cyclopentane*), 3.62 (3H, s, C*H_3_*-O-*pyrimidine*), 3.80 (3H, s, C*H_3_*-O-*phenyl*), 4.31-4.39 (1H, m, C*H-cyclopentane*), 4.57 (2H, d, *J* = 5.7 Hz, C*H_2_*-*benzyl*), 4.84-4.86 (1H, br s, N*H*), 6.86 (2H, d, *J* = 8.8 Hz, *H*-3 and *H*-5), 7.24 (2H, d, *J* = 8.8 Hz, *H*-2 and *H*-6), 8.09 (1H, s, *H-pyrimidine*); ^13^C-NMR (125 MHz, CDCl_3_) δ_C_ 23.8 (*C*H_2_*-cyclopentane* and *C*H_2_*-cyclopentane*), 33.9 (CH_2_-*C*H_2_-CH-*C*H_2_*-cyclopentane*), 44.4 (*C*H_2_-*benzyl*), 52.5 (*C*H*-cyclopentane*), 55.5 (O-*C*H_3_*-pyrimidine*), 58.4 (O-*C*H_3_-*phenyl*), 114.2 (*C*-3 and *C*-5), 122.3 (*C*-O*-pyrimidine*), 129.2 (*C*-2 and *C*-6), 131.7 (*C*-1), 153.6 (*C*H*-pyrimidine*), 154.1 (*C*-N), 154.3 (*C*-N), 159.1 (*C*-4); MS (ES^+^) *m/z* 329.2 [M+H]^+^.

*N*^4^-cyclopentyl-5-methoxypyrimidine-4,6-diamine **3b**

A solution of **3a** (14.0 mg, 0.042 mmol) in DCM (0.15 mL) and TFA (0.35 mL) was heated at 45 °C overnight. The reaction mixture was added drop-wise to a cool solution of NaHCO_3_ until pH = 9 was reached. The aqueous layer was extracted with DCM (3 × 10 mL). The combined organic layers were dried (MgSO_4_), filtered and concentrated *in vacuo*. The crude product was purified by flash chromatography (silica-gel column, 0-20% MeOH in DCM) to afford the title compound as light yellow oil (4.0 mg, 0.019 mmol, 50%). R_f_ = 0.36 (5% MeOH in DCM); UV λ_max_ (EtOH) nm: 221; ^1^H-NMR (500 MHz, CDCl_3_) δ_H_ 1.41-1.47 (2H, m, CH_2_-C*H*_2_-CH*-cyclopentane*), 1.60-1.76 (4H, m, C*H*_2_*-cyclopentane* and C*H*_2_*-cyclopentane*), 2.04-2.10 (2H, m, CH_2_-C*H*_2_-CH*-cyclopentane*), 3.67 (3H, s, O-C*H_3_*), 4.35 (1H, sex, *J* = 7.1 Hz, C*H-cyclopentane*), 4.56-4.63 (2H, br s, N*H_2_*), 4.75 (1H, br d, *J* = 7.1 Hz, N*H*), 7.96 (1H, s, *H-pyrimidine*); ^13^C-NMR (125 MHz, CDCl_3_) δ_C_ 23.8 (*C*H_2_*-cyclopentane* and *C*H_2_*-cyclopentane*), 33.8 (CH_2_-*C*H_2_-CH-*C*H_2_*-cyclopentane*), 52.5 (*C*H*-cyclopentane*), 58.4 (O-*C*H_3_), 122.5 (*C*-O), 153.5 (*C*H*-pyrimidine*), 154.2 (*C*-N), 155.3 (*C*-N); MS (ES^+^) *m/z* 209.2 [M+H]^+^.

4-chloro-6-(cyclopentylamino)pyrimidin-5-ol **4**

To a solution of **2** (640.0 mg, 2.81 mmol) in anhydrous DCM (2.5 mL) was added a solution of boron tribromide 1.0 M in DCM (14 mL, 14 mmol) drop-wise over a 30 min period. The reaction mixture was stirred at room temperature for 15 min and then heated at 40 °C for 15 h, cooled to 0 °C and slowly quenched with water (violent reaction). The pH was brought to pH = 6 with sat. aq. NaHCO_3_. The aqueous layer was extracted with DCM (3 × 30 mL). The combined organic layers were dried (MgSO_4_), filtered and concentrated *in vacuo* to afford the title compound as a light brown powder (588.0 mg, 2.75 mmol, 97%) which was carried forward without purification. R_f_ = 0.43 (50% EtOAc in 40-60 petrol); mp = 140.3 – 148.1 °C; UV λ_max_ (EtOH) nm: 255; IR ν_max_ cm^-1^: 3413, 3368 (NH), 2947, 2863, 2639, 2516, 2113, 1568, 1506, 1417, 1342, 1225, 1148, 1115, 1054; ^1^H-NMR (500 MHz, MeOD) δ_H_ 1.52-1.59 (2H, m, CH_2_-C*H*_2_-CH*-cyclopentane*), 1.61-1.68 (2H, m, C*H*_2_*-cyclopentane*), 1.74-1.82 (2H, m, C*H*_2_*-cyclopentane*), 2.02-2.08 (2H, m, CH_2_-C*H*_2_-CH*-cyclopentane*), 4.35 (1H, quin, *J* = 7.0 Hz, C*H-cyclopentane*), 7.85 (1H, s, *H-pyrimidine*), NH and OH not visible; ^13^C-NMR (125 MHz, MeOD) δ_C_ 24.7 (*C*H_2_*-cyclopentane* and *C*H_2_*-cyclopentane*), 33.6 (CH_2_-*C*H_2_-CH-*C*H_2_*-cyclopentane*), 53.9 (*C*H*-cyclopentane*), 134.4 (*C*-O), 139.6 (*C*-Cl), 149.9 (*C*H*-pyrimidine*), 156.5 (*C*-N); MS (ES^+^) *m/z* 214.1 [M(^35^Cl)+H]^+^ and *m/z* 216.1 [M(^37^Cl)+H]^+^, (ES^-^) *m/z* 212.0 [M(^35^Cl)+H]^-^ and *m/z* 214.0 [M(^37^Cl)+H]^-^.

6-chloro-*N*-cyclopentyl-5-isopropoxypyrimidin-4-amine **5**

To a suspension of **4** (0.100 g, 0.47 mmol) and K_2_CO_3_ (97.0 mg, 0.70 mmol) in MeCN (11 mL) and DMF (4 mL) was added 2-bromopropane (0.07 mL, 0.70 mmol). The reaction mixture was heated at 80 °C for 15 h, washed with water and sat. aq. NH_4_Cl (15 mL), extracted with DCM (3x 20 mL), dried (MgSO_4_), filtered and concentrated *in vacuo* to afford the title compound as a dark brown oil (116.0 mg, 0.45 mmol, 97%) which was carried forward without purification. UV λ_max_ (EtOH) nm: 251; IR ν_max_ cm^-1^: 3215, 3108 (NH), 2966, 2866, 1562, 1492, 1403, 1350, 1252, 1175, 1146, 1098; ^1^H-NMR (500 MHz, MeOD) δ_H_ 1.33 (6H, d, *J* = 6.1 Hz, CH(C*H_3_*)_2_), 1.53-1.60 (2H, m, CH_2_-C*H*_2_-CH*-cyclopentane*), 1.61-1.69 (2H, m, C*H*_2_*-cyclopentane*), 1.73-1.81 (2H, m, C*H*_2_*-cyclopentane*), 2.00-2.06 (2H, m, CH_2_-C*H*_2_-CH*-cyclopentane*), 4.38 (1H, quin, *J* = 7.1 Hz, C*H-cyclopentane*), 4.52 (1H, sept, *J* = 6.1 Hz, C*H*(CH_3_)_2_), 8.00 (1H, s, *H-pyrimidine*); ^13^C-NMR (125 MHz, MeOD) δ_C_ 22.5 (CH(*C*H_3_)_2_), 24.8 (*C*H_2_*-cyclopentane* and *C*H_2_*-cyclopentane*), 33.5 (CH_2_-*C*H_2_-CH-*C*H_2_*-cyclopentane*), 53.9 (*C*H*-cyclopentane*), 78.2 (*C*H(CH_3_)_2_), 134.9 (*C*-O), 148.5 (*C*-Cl), 153.3 (*C*H*-pyrimidine*), 160.3 (*C*-N); MS (ES^+^) *m/z* 256.1 [M(^35^Cl)+H]^+^ and *m/z* 258.1 [M(^37^Cl)+H]^+^.

*N*^4^-cyclopentyl-5-isopropoxy-*N*^6^-(4-methoxybenzyl)pyrimidine-4,6-diamine **6a**

To a solution of **5** (130.0 mg, 0.5 mmol) in anhydrous dioxane (1 mL) in a microwave vial were added p-methoxybenzylamine (0.22 mL, 1.5 mmol) and DIPEA (0.18 mL, 1 mmol). The resulting mixture heated at 220 °C for 2 h under microwave irradiation. The reaction mixture was diluted with DCM (10 mL) and washed with sat. aq. NH_4_Cl (10 mL). The aqueous layer was extracted with DCM (3 × 10 mL). The combined organic layers were dried (MgSO_4_), filtered and concentrated *in vacuo*. The crude product was purified by flash chromatography (silica-gel column, 0-5% MeOH in DCM) to afford the title compound as a pale oil (90 mg, 50%). UV λ_max_ (EtOH) nm: 229; IR ν_max_ cm^-1^: 3430 (NH), 2955, 2866, 1678, 1586, 1508, 1463, 1314, 1243, 1173, 1149, 1102, 1031; ^1^H-NMR (500 MHz, CDCl_3_) δ_H_ 1.26 (6H, d, *J* = 6.1 Hz, C(C*H_3_*)_2_), 1.39-1.46 (2H, m, CH_2_-C*H*_2_-CH*-cyclopentane*), 1.61-1.75 (4H, m, C*H*_2_*-cyclopentane* and C*H*_2_*-cyclopentane*), 2.02-2.09 (2H, m, CH_2_-C*H*_2_-CH*-cyclopentane*), 3.80 (3H, s, C*H_3_*-O-*phenyl*), 4.10 (1H, sept, *J* = 6.1 Hz, C*H*(CH_3_)_2_), 4.36 (1H, quin, *J* = 7.0 Hz, C*H-cyclopentane*), 4.56 (2H, d, *J* = 5.8 Hz, C*H_2_*-*benzyl*), 4.78 (1H, br t, *J* = 5.8 Hz, N*H*), 6.87 (2H, d, *J* = 8.7 Hz, *H*-3 and *H*-5), 7.27 (2H, d, *J* = 8.8 Hz, *H*-2 and *H*-6), 8.09 (1H, s, *H-pyrimidine*); ^13^C-NMR (125 MHz, CDCl_3_) δ_C_ 23.1 (C(*C*H_3_)_2_), 23.7 (*C*H_2_*-cyclopentane* and *C*H_2_*-cyclopentane*), 33.9 (CH_2_-*C*H_2_-CH-*C*H_2_*-cyclopentane*), 44.5 (*C*H_2_-*benzyl*), 52.5 (*C*H*-cyclopentane*), 55.4 (O-*C*H_3_-*phenyl*), 74.3 (*C*(CH_3_)_2_), 114.2 (*C*-3 and *C*-5), 119.9 (*C*-O-*pyrimidine*), 128.9 (*C*-2 and *C*-6), 131.8 (*C*-1), 153.1 (*C*H*-pyrimidine*), 155.0 (*C*-N), 155.1 (*C*-N), 159.0 (*C*-4); MS (ES^+^) *m/z* 357.2 [M+H]^+^.

*N*^4^-cyclopentyl-5-isopropoxypyrimidine-4,6-diamine **6b**

A solution of **6a** (90.0 mg, 0.25 mmol) in DCM (1 mL) and TFA (2 mL) was heated at 45 °C overnight. The reaction mixture was added drop-wise to a cool solution of NaHCO_3_ until pH = 9 was reached. The aqueous layer was extracted with DCM (3 × 10 mL). The combined organic layers were dried (MgSO_4_), filtered and concentrated *in vacuo*. The crude product was purified by flash chromatography (silica-gel column, 0-10% MeOH in DCM) to afford the title compound as light yellow oil (40.0 mg, 0.17 mmol, 67%). Rf = 0.36 (5% MeOH in DCM); UV λ_max_ (EtOH) nm: 221; IR ν_max_ cm^-1^: 3305 (NH), 3125, 2961, 2866, 2323, 1645, 1589; ^1^H-NMR (500 MHz, CDCl_3_) δ_H_ 1.27 (6H, d, *J* = 6.1 Hz, C(C*H_3_*)_2_), 1.37-1.43 (2H, m, CH_2_-C*H*_2_-CH*-cyclopentane*), 1.52-1.71 (4H, m, C*H*_2_*-cyclopentane* and C*H*_2_*-cyclopentane*), 2.00-2.05 (2H, m, CH_2_-C*H*_2_-CH*-cyclopentane*), 4.13 (1H, sept, *J* = 6.1 Hz, C*H*(CH_3_)_2_), 4.31 (1H, sex, *J* = 6.8 Hz, C*H-cyclopentane*), 4.65-4.70 (2H, br s, N*H_2_*), 4.70-4.75 (1H, br d, *J* = 7.4 Hz, N*H*), 7.93 (1H, s, *H-pyrimidine*); ^13^C-NMR (125 MHz, CDCl_3_) δ_C_ 23.0 (C(*C*H_3_)_2_), 23.7 (*C*H_2_*-cyclopentane* and *C*H_2_*-cyclopentane*), 33.7 (CH_2_-*C*H_2_-CH-*C*H_2_*-cyclopentane*), 52.4 (CH*-cyclopentane*), 74.1 (*C*(CH_3_)_2_), 120.1 (*C*-O), 153.0 (*C*H*-pyrimidine*), 155.0 (*C*-N), 156.3 (*C*-N); MS (ES^+^) *m/z* 237.1 [M+H]^+^.

5-(4-((*tert*-butyldiphenylsilyl)oxy)butoxy)-6-chloro-*N*-cyclopentylpyrimidin-4-amine **7**

To a suspension of **4** (0.100 g, 0.47 mmol) and K_2_CO_3_ (130.0 mg, 0.94 mmol) in MeCN (3.5 mL) and DMF (0.6 mL) was added *tert*-butyl(4-chlorobutoxy)diphenylsilane (243 mg, 0.70 mmol). The reaction mixture was heated at 80 °C for 15 h, washed with water and sat. aq. NH_4_Cl (10 mL), extracted with DCM (3x 10 mL), dried (MgSO_4_), filtered and concentrated *in vacuo*. The crude product was purified by flash chromatography (silica-gel column, 0-10% MeOH in DCM) to afford the title compound as a light orange oil (181.0 mg, 0.34 mmol, 73%). R_f_ = 0.51 (5% MeOH on DCM); ^1^H-NMR (500 MHz, CDCl_3_) δ_H_ 1.05 (9H, s, C(C*H_3_*)_3_), 1.40-1.47 (2H, m, CH_2_-C*H*_2_-CH*-cyclopentane*), 1.62-1.75 (6H, m, O-CH_2_-CH_2_-C*H_2_*-CH_2_-O, C*H_2_-cyclopentane* and C*H_2_-cyclopentane*), 1.86-1.91 (2H, m, O-CH_2_-C*H_2_*-CH_2_-CH_2_-O), 2.04-2.11 (2H, m, CH_2_-C*H*_2_-CH*-cyclopentane*), 3.73 (2H, t, *J* = 6.1 Hz, C*H_2_*-O), 3.99 (2H, t, *J* = 6.5 Hz, C*H_2_*-O), 4.35 (1H, sex, *J* = 6.9 Hz, C*H-cyclopentane*), 5.29 (1H, d, *J* = 6.9 Hz, N*H*), 7.36-7.46 (6H, m, H-Ar), 7.64-7.66 (4H, m, H-Ar), 8.14 (1H, s, *H-pyrimidine*); ^13^C-NMR (125 MHz, CDCl_3_) δ_C_ 19.2 (*C*(CH_3_)_3_), 23.6 (*C*H_2_*-cyclopentane* and *C*H_2_*-cyclopentane*), 26.7 (*C*H_2_), 26.9 (C(*C*H_3_)_3_), 29.1 (*C*H_2_), 33.3 (CH_2_-*C*H_2_-CH-*C*H_2_*-cyclopentane*), 52.8 (*C*H*-cyclopentane*), 63.2 (*C*H_2_-O), 73.1 (*C*H_2_-O), 127.7 (*C*H-Ar, *C*H-Ar, *C*H-Ar, and *C*H-Ar), 129.7 (*C*H-Ar, *C*H-Ar), 133.8 (*C*-Ar and *C*-Ar), 134.6 (*C*-O*-pyrimidine*), 135.6 (*C*H-Ar, *C*H-Ar, *C*H-Ar, and *C*H-Ar), 146.2 (*C*-Cl), 152.6 (*C*H*-pyrimidine*), 157.6 (*C*-N); MS (ES^+^) *m/z* 524.4 [M(^35^Cl)+H]^+^ and *m/z* 526.4 [M(^37^Cl)+H]^+^.

5-((5-((*tert*-butyldiphenylsilyl)oxy)pentyl)oxy)-6-chloro-*N*-cyclopentylpyrimidin-4-amine **8**

To a suspension of **4** (0.200 g, 0.94 mmol) and K_2_CO_3_ (230.0 mg, 1.87 mmol) in MeCN (3.5 mL) and DMF (0.6 mL) was added *tert*-butyl((5-chloropentyl)oxy)diphenylsilane (510 mg, 0.94 mmol). The reaction mixture was heated at 80 °C for 15 h, washed with water and sat. aq. NH_4_Cl (10 mL), extracted with DCM (3x 10 mL), dried (MgSO_4_), filtered and concentrated *in vacuo*. The crude product was purified by flash chromatography (silica-gel column, 0-10% MeOH in DCM) to afford the title compound as a light orange oil (143.0 mg, 0.27 mmol, 28%). R_f_ = 0.67 (5% MeOH on DCM); ^1^H-NMR (300 MHz, CDCl_3_) δ_H_ 1.04 (9H, s, C(C*H_3_*)_3_), 1.35-1.47 (2H, m, CH_2_-C*H*_2_-CH*-cyclopentane*), 1.51-1.79 (10H, m, O-CH_2_-C*H_2_*-C*H_2_*-C*H_2_*-CH_2_-O, C*H_2_-cyclopentane* and C*H_2_-cyclopentane*), 2.01-2.11 (2H, m, CH_2_-C*H*_2_-CH*-cyclopentane*), 3.68 (2H, t, *J* = 6.1 Hz, C*H_2_*-O), 3.95 (2H, t, *J* = 6.5 Hz, C*H_2_*-O), 4.33 (1H, sex, *J* = 6.9 Hz, C*H-cyclopentane*), 5.26 (1H, d, *J* = 6.9 Hz, N*H*), 7.36-7.46 (6H, m, H-Ar), 7.63-7.67 (4H, m, H-Ar), 8.12 (1H, s, *H-pyrimidine*); ^13^C-NMR (75 MHz, CDCl_3_) δ_C_ 19.2 (*C*(CH_3_)_3_), 22.4 (O-CH_2_-CH_2_-*C*H_2_-CH_2_-CH_2_-O), 23.6 (*C*H_2_*-cyclopentane* and *C*H_2_*-cyclopentane*), 26.9 (C(*C*H_3_)_3_), 32.2 (*C*H_2_), 33.2 (*C*H_2_), 33.3 (CH_2_-*C*H_2_-CH-*C*H_2_*-cyclopentane*), 52.7 (*C*H*-cyclopentane*), 63.6 (*C*H_2_-O), 73.1 (*C*H_2_-O), 127.7 (*C*H-Ar, *C*H-Ar, *C*H-Ar, and *C*H-Ar), 129.6 (*C*H-Ar, *C*H-Ar), 133.9 (*C*-Ar and *C*-Ar), 134.6 (*C*-O*-pyrimidine*), 135.6 (*C*H-Ar, *C*H-Ar, *C*H-Ar, and *C*H-Ar), 147.6 (*C*-Cl), 152.8 (*C*H*-pyrimidine*), 157.6 (*C*-N); MS (ES^+^) *m/z* 538.4 [M(^35^Cl)+H]^+^ and *m/z* 540.4 [M(^37^Cl)+H]^+^.

5-((6-((*tert*-butyldiphenylsilyl)oxy)hexyl)oxy)-6-chloro-*N*-cyclopentylpyrimidin-4-amine **9**

To a suspension of **4** (0.200 g, 0.94 mmol) and K_2_CO_3_ (230.0 mg, 1.87 mmol) in MeCN (3.5 mL) and DMF (0.6 mL) was added ((6-bromohexyl)oxy)(*tert*-butyl)diphenylsilane (590 mg, 0.7 mmol). The reaction mixture was heated at 80 °C for 15 h, washed with water and sat. aq. NH_4_Cl (10 mL), extracted with DCM (3x 10 mL), dried (MgSO_4_), filtered and concentrated *in vacuo*. The crude product was purified by flash chromatography (silica-gel column, 0-10% MeOH in DCM) to afford the title compound as a light orange oil (138.0 mg, 0.25 mmol, 27%). R_f_ = 0.58 (5% MeOH on DCM); ^1^H-NMR (300 MHz, CDCl_3_) δ_H_ 1.04 (9H, s, C(C*H_3_*)_3_), 1.38-1.49 (6H, m, O-CH_2_-CH_2_-C*H_2_*-C*H_2_*-CH_2_-CH_2_-O and CH_2_-C*H*_2_-CH*-cyclopentane*), 1.53-1.80 (8H, m, O-CH_2_-C*H_2_*-CH_2_-CH_2_-C*H_2_*-CH_2_-O, C*H_2_-cyclopentane* and C*H_2_-cyclopentane*), 2.02-2.14 (2H, m, CH_2_-C*H*_2_-CH*-cyclopentane*), 3.67 (2H, t, *J* = 6.3 Hz, C*H_2_*-O), 3.96 (2H, t, *J* = 6.5 Hz, C*H_2_*-O), 4.34 (1H, sex, *J* = 6.9 Hz, C*H-cyclopentane*), 5.29 (1H, d, *J* = 6.9 Hz, N*H*), 7.31-7.48 (6H, m, H-Ar), 7.61-7.71 (4H, m, H-Ar), 8.13 (1H, s, *H-pyrimidine*); ^13^C-NMR (75 MHz, CDCl_3_) δ_C_ 19.2 (*C*(CH_3_)_3_), 23.6 (*C*H_2_*-cyclopentane* and *C*H_2_*-cyclopentane*), 25.6 (O-CH_2_-CH_2_-*C*H_2_-CH_2_-CH_2_-CH_2_-O), 25.8 (O-CH_2_-CH_2_-CH_2_-*C*H_2_-CH_2_-CH_2_-O), 26.9 (C(*C*H_3_)_3_), 30.2 (O-CH_2_-*C*H_2_-CH_2_-CH_2_-CH_2_-CH_2_-O), 32.4 (O-CH_2_-CH_2_-CH_2_-CH_2_-*C*H_2_-CH_2_-O), 33.3 (CH_2_-*C*H_2_-CH-*C*H_2_*-cyclopentane*), 52.7 (*C*H*-cyclopentane*), 63.7 (*C*H_2_-O), 73.1 (*C*H_2_-O), 127.7 (*C*H-Ar, *C*H-Ar, *C*H-Ar, and *C*H-Ar), 129.6 (*C*H-Ar, *C*H-Ar), 134.0 (*C*-Ar and *C*-Ar), 134.6 (*C*-O*-pyrimidine*), 135.6 (*C*H-Ar, *C*H-Ar, *C*H-Ar, and *C*H-Ar), 147.6 (*C*-Cl), 152.8 (*C*H*-pyrimidine*), 157.6 (*C*-N); MS (ES^+^) *m/z* 551.4 [M(^35^Cl)+H]^+^ and *m/z* 552.4 [M(^37^Cl)+H]^+^.

5-(4-((*tert*-butyldiphenylsilyl)oxy)butoxy)-*N*^4^-cyclopentyl-*N*^6^-(2,4-dimethoxybenzyl) pyrimidine-4,6-diamine **10**

To a solution of **7** (170 mg, 0.32 mmol) in anhydrous dioxane (3 mL) in a microwave vial were added 2,4-dimethoxybenzylamine (0.15 mL, 0.97 mmol) and DIPEA (0.11 mL, 0.65 mmol). The resulting mixture was heated at 220 °C for 5 h under microwave irradiation. After addition of extra DIPEA (0.06 mL, 0.32 mmol), the reaction mixture was heated at 220 °C for 1 h under microwave irradiation. The reaction mixture was diluted with DCM (10 mL) and washed with sat. aq. NH_4_Cl (10 mL). The aqueous layer was extracted with DCM (3 × 10 mL). The combined organic layers were dried (MgSO_4_), filtered and concentrated *in vacuo*. The crude product was purified twice by flash chromatography (silica-gel column, 0-10% MeOH in DCM followed by silica-gel column, 0-50% EtOAc in petroleum ether) to afford the title compound as a light orange oil (70 mg, 0.11 mmol, 33%). R_f_ = 0.51 (5% MeOH in DCM). ^1^H-NMR (500 MHz, CDCl_3_) δ_H_ 1.05 (9H, s, C(C*H_3_*)_3_), 1.32-1.44 (2H, m, CH_2_-C*H*_2_-CH*-cyclopentane*), 1.50-1.75 (6H, m, O-CH_2_-CH_2_-C*H_2_*-CH_2_-O, C*H_2_-cyclopentane* and C*H_2_-cyclopentane*), 1.75-1.84 (2H, m, O-CH_2_-C*H_2_*-CH_2_-CH_2_-O), 1.98-2.10 (2H, m, CH_2_-C*H*_2_-CH*-cyclopentane*), 3.66-3.71 (4H, m, C*H_2_*-O and C*H_2_*-O), 3.77 (6H, s, C*H_3_*-O and C*H_3_*-O), 4.32 (1H, sex, *J* = 6.9 Hz, C*H-cyclopentane*), 4.56 (2H, d, *J* = 5.8 Hz, C*H_2_*-*benzyl*), 6.39-6.44 (2H, m, *H*-3 and *H*-5), 7.19-7.25 (1H, m, *H*-6), 7.34-7.46 (6H, m, H-Ar), 7.64-7.66 (4H, m, H-Ar), 8.08 (1H, s, *H-pyrimidine*); ^13^C-NMR (125 MHz, CDCl_3_) δ_C_ 19.2 (*C*(CH_3_)_3_), 23.6 (*C*H_2_*-cyclopentane* and *C*H_2_*-cyclopentane*), 26.1 (*C*H_2_), 26.9 (C(*C*H_3_)_3_), 29.2 (*C*H_2_), 33.7 (CH_2_-*C*H_2_-CH-*C*H_2_*-cyclopentane*), 37.5 (*C*H_2_-*benzyl*), 55.2 (O-*C*H_3_*-phenyl*), 55.3 (O-*C*H_3_*-phenyl*), 57.5 (*C*H*-cyclopentane*), 63.4 (*C*H_2_-O), 70.8 (*C*H_2_-O), 98.7 (*C*-3), 103.9 (*C*-5), 106.7 (*C*-1), 127.7 (*C*H-Ar, *C*H-Ar, *C*H-Ar, and *C*H-Ar), 129.7 (*C*H-Ar, *C*H-Ar), 130.2 (*C*-6), 133.4 (*C*-O*-pyrimidine*), 133.8 (*C*-Ar and *C*-Ar), 135.6 (*C*H-Ar, *C*H-Ar, *C*H-Ar, and *C*H-Ar), 152.0 (*C*H*-pyrimidine*), 155.3 (*C*-N), 155.5 (*C*-N), 158.6 (*C*-2), 163.2 (*C*-4); MS (ES^+^) *m/z* 656.5 [MH]^+^.

5-((5-((*tert*-butyldiphenylsilyl)oxy)pentyl)oxy)-*N*^4^-cyclopentyl-*N*^6^-(2,4-dimethoxybenzyl) pyrimidine-4,6-diamine **11**

To a solution of **9** (140 mg, 0.26 mmol) in anhydrous dioxane (2 mL) in a microwave vial were added 2,4-dimethoxybenzylamine (0.12 mL, 0.78 mmol) and DIPEA (0.09 mL, 0.52 mmol). The resulting mixture was heated at 220 °C for 7 h under microwave irradiation. The reaction mixture was diluted with DCM (10 mL) and washed with sat. aq. NH_4_Cl (10 mL). The aqueous layer was extracted with DCM (3 × 10 mL). The combined organic layers were dried (MgSO_4_), filtered and concentrated *in vacuo*. The crude product was purified by flash chromatography (silica-gel column, 0-80% EtOAc in petroleum ether) to afford the title compound as a light orange oil (71 mg, 0.11 mmol, 41%). R_f_ = 0.51 (5% MeOH in DCM). ^1^H-NMR (300 MHz, CDCl_3_) δ_H_ 1.04 (9H, s, C(C*H_3_*)_3_), 1.35-1.45 (2H, m, CH_2_-C*H*_2_-CH*-cyclopentane*), 1.45-1.73 (10H, m, O-CH_2_-C*H_2_*-C*H_2_*-C*H_2_*-CH_2_-O, C*H_2_-cyclopentane* and C*H_2_-cyclopentane*), 1.96-2.09 (2H, m, CH_2_-C*H*_2_-CH*-cyclopentane*), 3.68 (4H, m, C*H_2_*-O and C*H_2_*-O), 3.77 (3H, s, C*H_3_*-O), 3.78 (3H, s, C*H_3_*-O), 4.31 (1H, sex, *J* = 6.9 Hz, C*H-cyclopentane*), 4.54 (2H, d, *J* = 6.0 Hz, C*H_2_*-*benzyl*), 5.07 (1H, t, *J* = 6.0 Hz, CH_2_-N*H*), 6.39-6.44 (2H, m, *H*-3 and *H*-5), 7.20 (1H, d, *J* = 8.0 Hz, *H*-6), 7.36-7.46 (6H, m, H-Ar), 7.64-7.68 (4H, m, H-Ar), 8.07 (1H, s, *H-pyrimidine*).

5-((6-((*tert*-butyldiphenylsilyl)oxy)hexyl)oxy)-*N*^4^-cyclopentyl-*N*^6^-(2,4-dimethoxybenzyl)pyrimidine-4,6-diamine **12**

To a solution of **10** (135.0 mg, 0.24 mmol) in anhydrous dioxane (2 mL) in a microwave vial were added 2,4-dimethoxybenzylamine (0.12 mL, 0.72 mmol) and DIPEA (0.09 mL, 0.48 mmol). The resulting mixture was heated at 220 °C for 6 h under microwave irradiation. The reaction mixture was diluted with DCM (10 mL) and washed with sat. aq. NH_4_Cl (10 mL). The aqueous layer was extracted with DCM (3 × 10 mL). The combined organic layers were dried (MgSO_4_), filtered and concentrated *in vacuo*. The crude product was purified by flash chromatography (silica-gel column, 0-90% EtOAc in petroleum ether) to afford the title compound as a light orange oil (90 mg, 0.13 mmol, 54%). R_f_ = 0.51 (5% MeOH in DCM). ^1^H-NMR (300 MHz, CDCl_3_) δ_H_ 1.04 (9H, s, C(C*H_3_*)_3_), 1.33-1.43 (6H, m, O-CH_2_-CH_2_-C*H_2_*-C*H_2_*-CH_2_-CH_2_-O and CH_2_-C*H*_2_-CH*-cyclopentane*), 1.50-1.74 (8H, m, O-CH_2_-C*H_2_*-CH_2_-CH_2_-C*H_2_*-CH_2_-O, C*H_2_-cyclopentane* and C*H_2_-cyclopentane*), 1.97-2.09 (2H, m, CH_2_-C*H*_2_-CH*-cyclopentane*), 3.62-3.68 (4H, m, C*H_2_*-O and C*H_2_*-O), 3.76 (3H, s, C*H_3_*-O), 3.79 (3H, s, C*H_3_*-O), 4.32 (1H, sex, *J* = 6.9 Hz, C*H-cyclopentane*), 4.55 (2H, d, *J* = 6.0 Hz, C*H_2_*-*benzyl*), 5.05-5.13 (1H, br m, CH_2_-N*H*), 6.37-6.47 (2H, m, *H*-3 and *H*-5), 7.20 (1H, d, *J* = 8.2 Hz, *H*-6), 7.33-7.44 (6H, m, H-Ar), 7.64-7.68 (4H, m, H-Ar), 8.07 (1H, s, *H-pyrimidine*); ^13^C-NMR (75 MHz, CDCl_3_) δ_C_ 19.2 (*C*(CH_3_)_3_), 23.6 (*C*H_2_*-cyclopentane* and *C*H_2_*-cyclopentane*), 25.7 (O-CH_2_-CH_2_-*C*H_2_-CH_2_-CH_2_-CH_2_-O), 26.0 (O-CH_2_-CH_2_-CH_2_-*C*H_2_-CH_2_-CH_2_-O), 26.9 (C(*C*H_3_)_3_), 30.2 (O-CH_2_-*C*H_2_-CH_2_-CH_2_-CH_2_-CH_2_-O), 32.5 (O-CH_2_-CH_2_-CH_2_-CH_2_-*C*H_2_-CH_2_-O), 33.7 (CH_2_-*C*H_2_-CH-*C*H_2_*-cyclopentane*), 36.9 (*C*H_2_-*benzyl*), 52.9 (*C*H*-cyclopentane*), 55.4 (O-*C*H_3_*-phenyl*), 55.4 (O-*C*H_3_*-phenyl*), 63.7 (*C*H_2_-O), 70.4 (*C*H_2_-O), 98.6 (*C*-3), 103.8 (*C*-5), 107.8 (*C*-1), 127.6 (*C*H-Ar, *C*H-Ar, *C*H-Ar, and *C*H-Ar), 129.6 (*C*H-Ar, *C*H-Ar), 131.4 (*C*-6), 132.7 (*C*-O*-pyrimidine*), 134.0 (*C*-Ar and *C*-Ar), 135.6 (*C*H-Ar, *C*H-Ar, *C*H-Ar, and *C*H-Ar), 151.2 (*C*H*-pyrimidine*), 157.2 (*C*-N), 158.2 (*C*-N), 160.6 (*C*-2), 173.6 (*C*-4); MS (ES^+^) *m/z* 683.5 [M+H]^+^.

4-((4-amino-6-(cyclopentylamino)pyrimidin-5-yl)oxy)butan-1-ol **13**

A solution of **10** (60.0 mg, 0.091 mmol) in DCM (1.5 mL) and TFA (3.0 mL) was heated at 45 °C overnight. The reaction mixture was added drop-wise to a cool solution of NaHCO_3_ until pH = 9 was reached. The aqueous layer was extracted with DCM (3 × 10 mL). The combined organic layers were dried (MgSO_4_), filtered and concentrated *in vacuo*. The crude product was purified by flash chromatography (silica-gel column, 0-40% MeOH in DCM) and semi-preparative HPLC to afford the title compound an off-white oil (10.0 mg, 0.038 mmol, 41%). R_f_ = 0.16 (5% MeOH in DCM); UV λ_max_ (EtOH) nm: 222; IR ν_max_ cm^-1^: 3428, 3315, 3207, 3157, 2930, 2871, 1638, 1594; ^1^H-NMR (300 MHz, CDCl_3_) δ_H_ 1.36-1.48 (2H, m, CH_2_-C*H*_2_-CH*-cyclopentane*), 1.58-1.91 (8H, m, O-CH_2_-C*H_2_*-C*H_2_*-CH_2_-O, C*H_2_-cyclopentane* and C*H_2_-cyclopentane*), 2.00-2.11 (2H, m, CH_2_-C*H*_2_-CH*-cyclopentane*), 2.13-2.25 (1H, br s , O*H*), 3.75 (2H, t, *J* = 5.9 Hz, C*H_2_*-O), 3.83 (2H, t, *J* = 6.1 Hz, C*H_2_*-O), 4.35 (1H, sex, *J* = 6.8 Hz, C*H-cyclopentane*), 4.76 (2H, br s, N*H_2_*), 4.88 (1H, d, *J* = 6.8 Hz, N*H*), 8.01 (1H, s, *H-pyrimidine*); ^13^C-NMR (75 MHz, CDCl_3_) δ_C_ 23.6 (*C*H_2_*-cyclopentane* and *C*H_2_*-cyclopentane*), 27.3 (*C*H_2_), 29.3 (*C*H_2_), 33.6 (CH_2_-*C*H_2_-CH-*C*H_2_*-cyclopentane*), 57.4 (*C*H*-cyclopentane*), 62.6 (*C*H_2_-O), 70.9 (*C*H_2_-O), 116.2 (*C*-O*-pyrimidine*), 155.4 (*C*H*-pyrimidine*), 155.4 (*C*-N), 155.4 (*C*-N); MS (ES^+^) *m/z* 267.5 [MH]^+^.

5-((4-amino-6-(cyclopentylamino)pyrimidin-5-yl)oxy)pentan-1-ol **14**

A solution of **11** (60.0 mg, 0.090 mmol) in DCM (1.8 mL) and TFA (5.0 mL) was heated at 45 °C overnight. The reaction mixture was added drop-wise to a cool solution of NaHCO_3_ until pH = 9 was reached. The aqueous layer was extracted with DCM (3 × 10 mL). The combined organic layers were dried (MgSO_4_), filtered and concentrated *in vacuo*. The crude product was purified by flash chromatography (silica-gel column, 0-8% MeOH in DCM) to afford the title compound as a light orange oil (14.0 mg, 0.050 mmol, 55%). R_f_ = 0.44 (7% MeOH in DCM); UV λ_max_ (EtOH) nm: 222; IR ν_max_ cm^-1^: 3316, 3183, 2934, 2863, 1586; ^1^H-NMR (300 MHz, CDCl_3_) δ_H_ 1.35-1.47 (2H, m, CH_2_-C*H*_2_-CH*-cyclopentane*), 1.51-1.82 (10H, m, O-CH_2_-C*H_2_*-C*H_2_*-C*H_2_*-CH_2_-O, C*H_2_-cyclopentane* and C*H_2_-cyclopentane*), 2.00-2.11 (2H, m, CH_2_-C*H*_2_-CH*-cyclopentane*), 2.21-2.50 (1H, br s, O*H*), 3.68 (2H, t, *J* = 6.1 Hz, C*H_2_*-O), 3.78 (2H, t, *J* = 6.5 Hz, C*H_2_*-O), 4.33 (1H, sex, *J* = 6.9 Hz, C*H-cyclopentane*), 4.72-4.82 (3H, br m, N*H_2_* and N*H*), 7.94 (1H, s, *H-pyrimidine*); ^13^C-NMR (75 MHz, CDCl_3_) δ_C_ 22.5 (O-CH_2_-CH_2_-*C*H_2_-CH_2_-CH_2_-O), 23.7 (*C*H_2_*-cyclopentane* and *C*H_2_*-cyclopentane*), 30.1 (*C*H_2_), 32.4 (*C*H_2_), 33.7 (CH_2_-*C*H_2_-CH-*C*H_2_*-cyclopentane*), 52.5 (*C*H*-cyclopentane*), 62.5 (*C*H_2_-O), 73.1 (*C*H_2_-O), 121.4 (*C*-O), 152.9 (*C*H*-pyrimidine*), 154.2 (*C*-N), 155.6 (*C*-N); MS (ES^+^) *m/z* 281.3 [M+H]^+^.

6-((4-amino-6-(cyclopentylamino)pyrimidin-5-yl)oxy)hexan-1-ol **15**

A solution of **12** (80.0 mg, 0.12 mmol) in DCM (1.5 mL) and TFA (3.0 mL) was heated at 45 °C overnight. The reaction mixture was added drop-wise to a cool solution of NaHCO_3_ until pH = 9 was reached. The aqueous layer was extracted with DCM (3 × 10 mL). The combined organic layers were dried (MgSO_4_), filtered and concentrated *in vacuo*. The crude product was purified by flash chromatography (silica-gel column, 0-10% MeOH in DCM) to afford the title compound as a light orange oil (21.0 mg, 0.071 mmol, 61%). R_f_ = 0.37 (7% MeOH in DCM); UV λ_max_ (EtOH) nm: 222; IR ν_max_ cm^-1^: 3322, 3196, 2933, 2861, 1589; ^1^H-NMR (300 MHz, CDCl_3_) δ_H_ 1.36-1.53 (6H, m, O-CH_2_-CH_2_-C*H_2_*-C*H_2_*-CH_2_-CH_2_-O and CH_2_-C*H*_2_-CH*-cyclopentane*), 1.55-1.80 (8H, m, O-CH_2_-C*H_2_*-CH_2_-CH_2_-C*H_2_*-CH_2_-O, C*H_2_-cyclopentane* and C*H_2_-cyclopentane*), 2.01-2.13 (2H, m, CH_2_-C*H*_2_-CH*-cyclopentane*), 3.67 (2H, t, *J* = 6.6 Hz, C*H_2_*-O), 3.77 (2H, t, *J* = 6.8 Hz, C*H_2_*-O), 4.33 (1H, sex, *J* = 6.8 Hz, C*H-cyclopentane*), 4.68-4.81 (3H, br m, N*H_2_* and N*H*), 5.08-5.16 (1H, br m, N*H*), 7.95 (1H, s, *H-pyrimidine*), OH not visible; ^13^C-NMR (75 MHz, CDCl_3_) δ_C_ 23.7 (*C*H_2_*-cyclopentane* and *C*H_2_*-cyclopentane*), 25.7 (O-CH_2_-CH_2_-*C*H_2_-CH_2_-CH_2_-CH_2_-O), 26.1 (O-CH_2_-CH_2_-CH_2_-*C*H_2_-CH_2_-CH_2_-O), 30.4 (O-CH_2_-*C*H_2_-CH_2_-CH_2_-CH_2_-CH_2_-O), 32.7 (O-CH_2_-CH_2_-CH_2_-CH_2_-*C*H_2_-CH_2_-O), 33.8 (CH_2_-*C*H_2_-CH-*C*H_2_*-cyclopentane*), 52.5 (*C*H*-cyclopentane*), 62.8 (*C*H_2_-O), 71.3 (*C*H_2_-O), 121.4 (*C*-O*-pyrimidine*), 152.8 (*C*H*-pyrimidine*), 154.1 (*C*-N), 155.6 (*C*-N); MS (ES^+^) *m/z* 295.3 [M+H]^+^.

6-chloro-*N*-cyclopentyl-5-(2-(dimethylamino)ethoxy)pyrimidin-4-amine **16**

To a stirred suspension of **4** (100.0 mg, 0.47 mmol) and K_2_CO_3_ (161.0 mg, 1.71 mmol) in MeCN (5 mL) and DMF (2 mL) was added 2-chloro-N,N-dimethylamine hydrochloride (100.0 mg, 0.70 mmol). The reaction mixture was heated at 80 °C for 2 h, washed with water and sat. aq. NH_4_Cl (10 mL), extracted with DCM (3x 10 mL), dried (MgSO_4_), filtered and concentrated *in vacuo*. The crude product was purified by flash chromatography (silica-gel column, 0-10% MeOH in DCM) to afford the title compound as a white solid (70 mg, 0.25 mmol, 53%). mp = 150.6 – 151.2 °C; UV λ_max_ (EtOH) nm: 253; IR ν_max_ cm^-1^: 3350, 3250, 2952, 2860, 1671, 1609, 1576, 1524, 1446, 1429, 1343; ^1^H-NMR (500 MHz, CDCl_3_) δ_H_ 1.38-1.46 (2H, m, CH_2_-C*H*_2_-CH*-cyclopentane*), 1.61-1.77 (4H, m, C*H*_2_*-cyclopentane*), 2.06-2.15 (2H, m, CH_2_-C*H*_2_-CH*-cyclopentane*), 2.32 (6H, s, N(C*H_3_*)_2_), 2.59 (2H, t, *J* = 4.1 Hz, C*H_2_*-N), 3.99 (2H, t, *J* = 4.1 Hz, C*H_2_*-O), 4.28 (1H, sex, *J* = 7.1 Hz, C*H-cyclopentane*), 7.80 (1H, br m, N*H*), 8.12 (1H, s, *H-pyrimidine*); ^13^C-NMR (125 MHz, CDCl_3_) δ_C_ 23.8 (*C*H_2_*-cyclopentane* and *C*H_2_*-cyclopentane*), 33.8 (CH_2_-*C*H_2_-CH-*C*H_2_*-cyclopentane*), 45.6 (N(*C*H_3_)_2_), 52.5 (*C*H*-cyclopentane*), 59.5 (*C*H_2_-N), 70.9 (*C*H_2_-O), 134.6 (*C*-O), 148.3 (*C*-Cl), 153.8 (*C*H*-pyrimidine*), 158.2 (*C*-N); MS (ES^+^) *m/z* 285.2 [M(^35^Cl)+H]^+^ and *m/z* 287.2 [M(^37^Cl)+H]^+^.

*N*-cyclopentyl-8-methyl-7,8-dihydro-6*H*-pyrimido[5,4-*b*][1,4]oxazin-4-amine **17**

To a stirred suspension of **4** (240.0 mg, 1.40 mmol) and K_2_CO_3_ (582.0 mg, 4.20 mmol) in MeCN (15 mL) and DMF (6 mL) was added 2-chloro-N,N-dimethylamine hydrochloride (302.0 mg, 2.10 mmol). The reaction mixture was heated at 80 °C overnight, washed with water and sat. aq. NH_4_Cl (10 mL), extracted with DCM (3x 10 mL), dried (MgSO_4_), filtered and concentrated *in vacuo*. The crude product was purified by flash chromatography (silica-gel column, 0-40% EtOAc in 40-60 petrol, then 100% MeOH) to afford the title compound as a light brown oil (240 mg, 1.02 mmol, 73%). R_f_ = 0.65 (10% MeOH in DCM); UV λ_max_ (EtOH) nm: 228; IR ν_max_ cm^-1^: 3427, 2945, 2863, 1596, 1515, 1477, 1445, 1406, 1372, 1339; ^1^H-NMR (500 MHz, CDCl_3_) δ_H_ 1.37-1.43 (2H, m, CH_2_-C*H*_2_-CH*-cyclopentane*), 1.55-1.74 (4H, m, C*H*_2_*-cyclopentane*), 1.98-2.06 (2H, m, CH_2_-C*H*_2_-CH*-cyclopentane*), 3.04 (3H, s, N-C*H_3_*), 3.37 (2H, t, *J* = 4.5 Hz, C*H_2_*-N), 4.17 (2H, t, *J* = 4.5 Hz, C*H_2_*-O), 4.30 (1H, sex, *J* = 6.7 Hz, C*H-cyclopentane*), 4.56 (1H, d, *J* = 6.7 Hz, N*H*), 7.93 (1H, s, *H-pyrimidine*); ^13^C-NMR (125 MHz, CDCl_3_) δ_C_ 23.6 (*C*H_2_*-cyclopentane* and *C*H_2_*-cyclopentane*), 33.7 (CH_2_-*C*H_2_-CH-*C*H_2_*-cyclopentane*), 35.2 (N-*C*H_3_), 48.2 (*C*H_2_-N), 52.3 (*C*H*-cyclopentane*), 63.7 (*C*H_2_-O), 120.4 (*C*-O), 148.3 (*C*-N), 150.1 (*C*H*-pyrimidine*), 150.2 (*C*-N); MS (ES^+^) *m/z* 235.2 [M+H]^+^.

4-(cyclopentylamino)-6-(dimethyliminio)-1-vinyl-1,6-dihydropyrimidin-5-olate **18**

To a stirred suspension of **4** (240.0 mg, 1.40 mmol) and K_2_CO_3_ (582.0 mg, 4.20 mmol) in MeCN (15 mL) and DMF (6 mL) was added 2-chloro-N,N-dimethylamine hydrochloride (302.0 mg, 2.10 mmol). The reaction mixture was heated at 80 °C overnight, washed with water and sat. aq. NH_4_Cl (10 mL), extracted with DCM (3x 10 mL), dried (MgSO_4_), filtered and concentrated *in vacuo*. The crude product was purified by flash chromatography (silica-gel column, 0-40% EtOAc in 40-60 petrol, then 100% MeOH) to afford the title compound as a dark brown oil (54.0 mg, 0.22 mmol, 15%). UV λ_max_ (EtOH) nm: 226; IR ν_max_ cm^-1^: 3339, 2953, 2868, 1605, 1548, 1518, 1447, 1396, 1334; ^1^H-NMR (500 MHz, CDCl_3_) δ_H_ 1.44-1.52 (2H, m, CH_2_-C*H*_2_-CH*-cyclopentane*), 1.53-1.74 (4H, m, C*H*_2_*-cyclopentane*), 1.96-2.04 (2H, m, CH_2_-C*H*_2_-CH*-cyclopentane*), 3.65 (6H, s, N(C*H_3_*)*_2_*), 4.26 (1H, sex, *J* = 6.9 Hz, C*H-cyclopentane*), 5.32 (1H, dd, *J* = 8.1 and 3.9 Hz, H_b_), 5.47 (1H, dd, *J* = 15.4 and 3.9 Hz, H_c_), 6.27 (1H, d, *J* = 6.9 Hz, N*H*), 6.98 (1H, dd, *J* = 15.4 and 8.1 Hz, N-C*H_a_*=CH_2_), 7.47 (1H, s, *H-pyrimidine*); ^13^C-NMR (125 MHz, CDCl_3_) δ_C_ 23.7 (*C*H_2_*-cyclopentane* and *C*H_2_*-cyclopentane*), 33.2 (CH_2_-*C*H_2_-CH-*C*H_2_*-cyclopentane*), 51.1 (N(*C*H_3_)_2_), 52.3 (*C*H*-cyclopentane*), 108.7 (*C*H_2_-*vinyl*), 136.1 (*C*-O), 136.4 (*C*H*-pyrimidine*), 142.1 (*C*H-*vinyl*), 142.6 (*C*-N), 161.9 (*C*-NH); MS (ES^+^) *m/z* 249.3 [M+H]^+^.

6-chloro-*N*-cyclopentyl-5-(vinyloxy)pyrimidin-4-amine **19**

To a stirred suspension of **4** (240.0 mg, 1.40 mmol) and K_2_CO_3_ (582.0 mg, 4.20 mmol) in MeCN (15 mL) and DMF (6 mL) was added 2-chloro-N,N-dimethylamine hydrochloride (302.0 mg, 2.10 mmol). The reaction mixture was heated at 80 °C overnight, washed with water and sat. aq. NH_4_Cl (10 mL), extracted with DCM (3x 10 mL), dried (MgSO_4_), filtered and concentrated *in vacuo*. The crude product was purified by flash chromatography (silica-gel column, 0-40% EtOAc in 40-60 petrol, then 100% MeOH) to afford the title compound as a light brown oil (5.0 mg, 0.021 mmol, 1%). UV λ_max_ (EtOH) nm: 249; IR ν_max_ cm^-1^: 3427, 3279, 2954, 2868, 1627, 1569, 1498, 1409, 1337; ^1^H-NMR (500 MHz, CDCl_3_) δ_H_ 1.44-1.49 (2H, m, CH_2_-C*H*_2_-CH*-cyclopentane*), 1.56-1.76 (4H, m, C*H*_2_*-cyclopentane*), 2.04-2.12 (2H, m, CH_2_-C*H*_2_-CH*-cyclopentane*), 4.34-4.41 (1H, m, C*H-cyclopentane*), 4.40 (1H, dd, *J* = 6.3 and 2.9 Hz, H_b_), 4.46 (1H, dd, *J* = 13.9 and 2.9 Hz, H_c_), 5.04-5.13 (1H, br m, N*H*), 6.46 (1H, dd, *J* = 13.9 and 6.3 Hz, O-C*H_a_*=CH_2_), 8.22 (1H, s, *H-pyrimidine*); ^13^C-NMR (125 MHz, CDCl_3_) δ_C_ 23.6 (*C*H_2_*-cyclopentane* and *C*H_2_*-cyclopentane*), 33.2 (CH_2_-*C*H_2_-CH-*C*H_2_*-cyclopentane*), 52.9 (*C*H*-cyclopentane*), 92.7 (*C*H_2_-*vinyl*), 130.4 (*C*-O), 148.1 (*C*H-*vinyl*), 149.0 (*C*-Cl) 154.2 (*C*H*-pyrimidine*), 156.6 (*C*-N); MS (ES^+^) *m/z* 240.2 [M(^35^Cl)+H]^+^ and *m/z* 242.2 [M(^37^Cl)+H]^+^.

*N*^4^-cyclopentyl-*N*^6^,*N*^6^-dimethyl-5-(vinyloxy)pyrimidine-4,6-diamine **20**

To a stirred suspension of **4** (240.0 mg, 1.40 mmol) and K_2_CO_3_ (582.0 mg, 4.20 mmol) in MeCN (15 mL) and DMF (6 mL) was added 2-chloro-N,N-dimethylamine hydrochloride (302.0 mg, 2.10 mmol). The reaction mixture was heated at 80 °C overnight, washed with water and sat. aq. NH_4_Cl (10 mL), extracted with DCM (3x 10 mL), dried (MgSO_4_), filtered and concentrated *in vacuo*. The crude product was purified by flash chromatography (silica-gel column, 0-40% EtOAc in 40-60 petrol, then 100% MeOH) to afford the title compound as a brown oil (6.0 mg, 0.024 mmol, 1.7%). UV λ_max_ (EtOH) nm: 236; IR ν_max_ cm^-1^: 3436, 2950, 2866, 1583, 1486, 1412, 1326; ^1^H-NMR (500 MHz, CDCl_3_) δ_H_ 1.37-1.43 (2H, m, CH_2_-C*H*_2_-CH*-cyclopentane*), 1.55-1.74 (4H, m, C*H*_2_*-cyclopentane*), 1.98-2.06 (2H, m, CH_2_-C*H*_2_-CH*-cyclopentane*), 3.07 (6H, s, N(C*H_3_*)*_2_*), 4.27 (1H, dd, *J* = 6.4 and 2.3 Hz, H_b_), 4.31 (1H, sex, *J* = 6.9 Hz, C*H-cyclopentane*), 4.41 (1H, dd, *J* = 13.9 and 2.3 Hz, H_c_), 4.65 (1H, d, *J* = 6.9 Hz, N*H*), 6.31 (1H, dd, *J* = 13.9 and 6.4 Hz, O-C*H_a_*=CH_2_), 8.07 (1H, s, *H-pyrimidine*); ^13^C-NMR (125 MHz, CDCl_3_) δ_C_ 23.6 (*C*H_2_*-cyclopentane* and *C*H_2_*-cyclopentane*), 33.6 (CH_2_-*C*H_2_-CH-*C*H_2_*-cyclopentane*), 39.2 (N(*C*H_3_)_2_), 48.2 (*C*H_2_-N), 52.5 (*C*H*-cyclopentane*), 92.0 (*C*H_2_-*vinyl*), 118.9 (*C*-O), 150.0 (*C*H-*vinyl*), 153.3 (*C*-N), 154.7 (*C*H*-pyrimidine*), 156.1 (*C*-N); MS (ES^+^) *m/z* 249.3 [M+H]^+^.

6-chloro-*N*-cyclopentyl-5-(3-(dimethylamino)propoxy)pyrimidin-4-amine **21**

To a stirred suspension of **4** (100.0 mg, 0.47 mmol) and K_2_CO_3_ (161.0 mg, 1.71 mmol) in MeCN (5 mL) and DMF (2 mL) was added 3-dimethylamino-1-propylchloride hydrochloride (110.0 mg, 0.70 mmol). The reaction mixture was heated at 80 °C for 3 h, washed with water and sat. aq. NH_4_Cl (15 mL), extracted with DCM (3x 20 mL), dried (MgSO_4_), filtered and concentrated *in vacuo*. The crude product was purified by flash chromatography (silica-gel column, 0-20% MeOH in DCM) to afford the title compound as a dark yellow oil (98 mg, 0.33 mmol, 70%). R_f_ = 0.27 (5% MeOH in DCM); UV λ_max_ (EtOH) nm: 251; IR ν_max_ cm^-1^: 3215, 3124, 2947, 2864, 2821, 2776, 1568, 1498, 1445, 1405, 1337; ^1^H-NMR (500 MHz, CDCl_3_) δ_H_ 1.38-1.46 (2H, m, CH_2_-C*H*_2_-CH*-cyclopentane*), 1.60-1.77 (4H, m, C*H*_2_*-cyclopentane*), 1.95 (2H, quin, *J* = 6.0 Hz, CH_2_-C*H_2_*-CH_2_), 2.04-2.12 (2H, m, CH_2_-C*H*_2_-CH*-cyclopentane*), 2.32 (6H, s, N(C*H_3_*)_2_), 2.52 (2H, t, *J* = 6.0 Hz, C*H_2_*-N), 3.93 (2H, t, *J* = 6.0 Hz, C*H_2_*-O), 4.44 (1H, sex, *J* = 7.1 Hz, C*H-cyclopentane*), 6.83 (1H, d, *J* = 7.1 Hz, N*H*), 8.12 (1H, s, *H-pyrimidine*); ^13^C-NMR (125 MHz, CDCl_3_) δ_C_ 23.8 (*C*H_2_*-cyclopentane* and *C*H_2_*-cyclopentane*), 26.7 (CH_2_-*C*H_2_-CH_2_), 32.8 (CH_2_-*C*H_2_-CH-*C*H_2_*-cyclopentane*), 45.5 (N(*C*H_3_)_2_), 52.3 (*C*H*-cyclopentane*), 56.0 (*C*H_2_-N), 68.7 (*C*H_2_-O), 133.9 (*C*-O), 148.4 (*C*-Cl), 153.1 (*C*H*-pyrimidine*), 157.7 (*C*-N); MS (ES^+^) *m/z* 299.2 [M(^35^Cl)+H]^+^ and *m/z* 301.2 [M(^37^Cl)+H]^+^.

*N*^4^-cyclopentyl-5-(3-(dimethylamino)propoxy)-*N*^6^-(4-methoxybenzyl)pyrimidine-4,6-diamine **22**

To a stirred solution of **21** (98.0 mg, 0.33 mmol) in anhydrous dioxane (1.6 mL) in a microwave vial were added p-methoxybenzylamine (0.13 mL, 0.98 mmol) and DIPEA (0.11 mL, 0.66 mmol). The resulting mixture was heated at 220 °C for 2 h under microwave irradiation. The reaction mixture was diluted with DCM (10 mL) and washed with sat. aq. NH_4_Cl (10 mL). The aqueous layer was extracted with DCM (3 × 10 mL). The combined organic layers were dried (MgSO_4_), filtered and concentrated *in vacuo*. The crude product was purified by flash chromatography (silica-gel column, 0-5% MeOH in DCM) to afford the title compound as a pale oil (8.0 mg, 0.02 mmol, 6%). Rf = 0.3 (5% MeOH in DCM); UV λ_max_ (EtOH) nm: 234; IR ν_max_ cm^-1^: 3247, 2951, 2866, 1670, 1596, 1510, 1458, 1317; ^1^H-NMR (500 MHz, CDCl_3_) δ_H_ 1.37-1.44 (2H, m, CH_2_-C*H*_2_-CH*-cyclopentane*), 1.57-1.74 (4H, m, C*H*_2_*-cyclopentane* and C*H*_2_*-cyclopentane*), 1.89 (2H, quin, *J* = 6.2 Hz, N-CH_2_-C*H_2_*-CH_2_-O), 2.00-2.05 (8H, m, N(C*H_3_*)_2_ and CH_2_-C*H*_2_-CH*-cyclopentane*), 2.46 (2H, t, *J* = 6.2 Hz, C*H_2_*-N), 3.74-3.81 (5H, m, C*H_3_*-O-phenyl and C*H_2_*-O), 4.37 (1H, sex, *J* = 7.3 Hz, C*H-cyclopentane*), 4.59 (2H, d, *J* = 5.8 Hz, C*H_2_*-*benzyl*), 4.57 (1H, d, *J* = 7.3 Hz, N*H*), 6.76-6.80 (1H, br m, CH_2_-N*H*), 6.81 (2H, d, *J* = 8.7 Hz, *H*-3 and *H*-5), 7.19 (2H, d, *J* = 8.7 Hz, *H*-3 and *H*-5), 8.05 (1H, s, *H-pyrimidine*); ^13^C-NMR (125 MHz, CDCl_3_) δ_C_ 23.8 (*C*H_2_*-cyclopentane* and *C*H_2_*-cyclopentane*), 26.7 (CH_2_-*C*H_2_-CH_2_), 33.7 (CH_2_-*C*H_2_-CH_2_-O), 33.7 (CH_2_-*C*H_2_-CH-*C*H_2_*-cyclopentane*), 44.0 (*C*H_2_-*benzyl*), 45.1 (N(*C*H_3_)_2_), 52.3 (*C*H*-cyclopentane*), 55.4 (O-*C*H_3_*-phenyl*), 55.4 (*C*H_2_-N), 66.8 (*C*H_2_-O), 114.0 (*C*-3 and *C*-5), 120.5 (*C*-O*-pyrimidine*), 128.7 (*C*-2 and *C*-6), 132.6 (*C*-1), 153.4 (*C*H*-pyrimidine*), 154.5 (*C*-N), 154.7 (*C*-N), 158.8 (*C*-4); MS (ES^+^) *m/z* 400.3 [M+H]^+^.

*N*-cyclopentyl-9-methyl-6,7,8,9-tetrahydropyrimido[5,4-*b*][1,4]oxazepin-4-amine **23**

To a stirred solution of **21** (98.0 mg, 0.33 mmol) in anhydrous dioxane (1.6 mL) in a microwave vial were added p-methoxybenzylamine (0.13 mL, 0.98 mmol) and DIPEA (0.11 mL, 0.66 mmol). The resulting mixture was heated at 220 °C for 2 h under microwave irradiation. The reaction mixture was diluted with DCM (10 mL) and washed with sat. aq. NH_4_Cl (10 mL). The aqueous layer was extracted with DCM (3 × 10 mL). The combined organic layers were dried (MgSO_4_), filtered and concentrated *in vacuo*. The crude product was purified by flash chromatography (silica-gel column, 0-5% MeOH in DCM) to afford the title compound as a pale oil (53.0 mg, 0.21 mmol, 64%). UV λ_max_ (EtOH) nm: 234; IR ν_max_ cm^-1^: 3429, 2948, 2865, 1582, 1478, 1437, 1404, 1377, 1319; ^1^H-NMR (500 MHz, CDCl_3_) δ_H_ 1.38-1.46 (2H, m, CH_2_-C*H*_2_-CH*-cyclopentane*), 1.60-1.74 (4H, m, C*H*_2_*-cyclopentane*), 2.01-2.12 (4H, m, CH_2_-C*H_2_*-CH_2_ and CH_2_-C*H*_2_-CH*-cyclopentane*), 3.06 (3H, s, N-C*H_3_*), 3.43-3.46 (2H, m, C*H_2_*-N), 4.14 (2H, t, *J* = 6.2 Hz, C*H_2_*-O), 4.31 (1H, sex, *J* = 7.1 Hz, C*H-cyclopentane*), 4.95 (1H, d, *J* = 7.1 Hz, N*H*), 8.02 (1H, s, *H-pyrimidine*); ^13^C-NMR (125 MHz, CDCl_3_) δ_C_ 23.7 (*C*H_2_*-cyclopentane* and *C*H_2_*-cyclopentane*), 29.2 (CH_2_-*C*H_2_-CH_2_), 33.6 (CH_2_-*C*H_2_-CH-*C*H_2_*-cyclopentane*), 38.7 (N-*C*H_3_), 50.4 (*C*H_2_-N), 52.4 (*C*H*-cyclopentane*), 70.5 (*C*H_2_-O), 129.5 (*C*-O), 151.4 (*C*H*-pyrimidine*), 157.5 (*C*-N), 155.0 (*C*-N); MS (ES^+^) *m/z* 249.1 [M+H]^+^.

*tert*-butyl 4-(2-((4-chloro-6-(cyclopentylamino)pyrimidin-5-yl)oxy)ethyl)piperidine-1-carboxylate **24**

To a stirred suspension of **4** (100.0 mg, 0.47 mmol) and K_2_CO_3_ (96 mg, 0.71 mmol) in MeCN (5 mL) and DMF (1 mL) was added *N*-Boc-4-(2-bromoethyl)piperidine (205.0 mg, 0.71 mmol). The reaction mixture was heated at 80 °C for 16 h, washed with water and sat. aq. NH_4_Cl (15 mL), extracted with DCM (3x 20 mL), dried (MgSO_4_), filtered and concentrated *in vacuo*. The crude product was purified by flash chromatography (silica-gel column, 0-20% MeOH in DCM) to afford the title compound as a yellow oil (155 mg, 0.36 mmol, 78%). R_f_ = 0.27 (5% MeOH in DCM); ^1^H-NMR (500 MHz, CDCl_3_) δ_H_ 1.12-1.21 (2H, m, CH-C*H*_2_-*piperidine* and CH-C*H*_2_-*piperidine*), 1.39-1.48 (11H, m, C(C*H_3_*)_3_ and CH_2_-C*H_2_*-CH*-cyclopentane*), 1.61-1.76 (9H, m, CH-C*H_2_*, C*H*-*piperidine*, CH-C*H*_2_-*piperidine* and CH-C*H*_2_-*piperidine*, C*H_2_-cyclopentane* and C*H_2_-cyclopentane*), 2.04-2.12 (2H, m, CH_2_-C*H_2_*-CH*-cyclopentane*), 2.65-2.76 (2H, m, N-C*H*_2_-*piperidine* and N-C*H*_2_-*piperidine*), 4.03 (2H, t, *J* = 6.4 Hz, C*H_2_*-O), 4.05-4.20 (2H, m, N-C*H*_2_-*piperidine* and N-C*H*_2_-*piperidine*), 4.35 (1H, sex, *J* = 7.2 Hz, C*H-cyclopentane*), 5.25 (1H, d, *J* = 7.2 Hz, N*H*), 8.13 (1H, s, *H-pyrimidine*); ^13^C-NMR (125 MHz, CDCl_3_) δ_C_ 23.8 (*C*H_2_*-cyclopentane* and *C*H_2_*-cyclopentane*), 28.6 (C(*C*H_3_)_3_), 32.3 (CH-*C*H_2_-*piperidine* and CH-*C*H_2_-*piperidine*), 33.0 (*C*H-*piperidine*), 33.5 (CH_2_-*C*H_2_-CH-*C*H_2_*-cyclopentane*), 36.9 (CH-*C*H_2_), 44.1 (N-*C*H_2_-*piperidine* and N-*C*H_2_-*piperidine*), 52.8 (*C*H*-cyclopentane*), 70.6 (*C*H_2_-O), 79.6 (*C*(CH_3_)_3_), 134.6 (*C*-O), 147.4 (*C*-Cl), 153.1 (*C*H*-pyrimidine*), 154.8 (*C*=O), 157.8 (*C*-N); MS (ES^+^) *m/z* 425.3 [M(^35^Cl)+H]^+^, *m/z* 427.3 [M(^37^Cl)+H]^+^ and *m/z* 369.2 [M(^35^Cl)-*t*Bu+H]^+^.

4-(cyclopentylamino)-6,7-dihydrospiro[pyrimido[5,4-*b*][1,4]oxazine-8,1'-pyrrolidin]-8-ium chloride **25**

To a stirred suspension of **4** (100.0 mg, 0.47 mmol) and K_2_CO_3_ (162.0 mg, 1.17 mmol) in MeCN (5 mL) and DMF (1 mL) was added 1-(2-bromoethyl)pyrrolidine hydrobromide (181.0 mg, 0.70 mmol). The reaction mixture was heated at 80 °C overnight, washed with sat. aq. NH_4_Cl (10 mL). The aqueous layer was extracted with DCM (3x 10 mL), brought to pH = 9 with sat. aq. NaHCO_3_, extracted with CHCl_3_/IPA 7/3 (1 x 10 mL), brought to pH = 2 with 1 M HCl, extracted with CHCl_3_/IPA 7/3 (1 x 10 mL), brought to pH = 6 with 2 M NaOH, extracted with CHCl_3_/IPA 7/3 (1 x 10 mL). The combined organic layers were dried (MgSO_4_), filtered and concentrated *in vacuo*. The crude product was purified by flash chromatography (silica-gel column, 0-10% MeOH in DCM) to afford the title compound as a light oil (94 mg, 0.30 mmol, 73%). UV λ_max_ (EtOH) nm: 244; IR ν_max_ cm^-1^: 3368, 3248, 2945, 2867, 1620, 1509, 1448, 1365, 1318; ^1^H-NMR (500 MHz, CDCl_3_) δ_H_ 1.51-1.59 (2H, m, CH_2_-C*H_2_*-CH*-cyclopentane*), 1.62-1.69 (2H, m, C*H*_2_*-cyclopentane*), 1.73-1.85 (2H, m, C*H*_2_*-cyclopentane*), 1.93-2.00 (2H, m, CH_2_-C*H_2_-pyrrolidine*), 2.03-2.11 (4H, m, CH_2_-C*H_2_-pyrrolidine* and CH_2_-C*H_2_*-CH*-cyclopentane*), 3.51-3.55 (4H, m, N-C*H_2_*-CH_2_-O, N-C*H*_2_*-pyrrolidine* and N-C*H*_2_*-pyrrolidine*), 4.33-4.38 (2H, m, C*H_2_*-O), 4.47 (1H, sex, *J* = 6.7 Hz, C*H-cyclopentane*), 4.55-4.61 (2H, m, N-C*H*_2_*-pyrrolidine* and N-C*H*_2_*-pyrrolidine*), 6.28 (1H, d, *J* = 6.7 Hz, N*H*), 8.63 (1H, s, *H-pyrimidine*); ^13^C-NMR (125 MHz, CDCl_3_) δ_C_ 23.9 (*C*H_2_*-cyclopentane* and *C*H_2_*-cyclopentane*), 25.2 (*C*H_2_-CH_2_*-pyrrolidine*), 25.6 (CH_2_-*C*H_2_*-pyrrolidine*), 33.2 (CH_2_-*C*H_2_-CH-*C*H_2_*-cyclopentane*), 51.3 (*C*H_2_-N), 52.8 (N-*C*H_2_*-pyrrolidine*), 53.5 (*C*H*-cyclopentane*), 56.6 (N-*C*H_2_*-pyrrolidine*), 63.7 (*C*H_2_-O), 123.8 (*C*-O), 141.9 (*C*-N), 146.5 (*C*H*-pyrimidine*), 152.5 (*C*-NH); MS (ES^+^) *m/z* 275.2 [M-Cl]^+^.

*N*-cyclopentyl-8-(4-((4-methoxybenzyl)amino)butyl)-7,8-dihydro-6*H*-pyrimido[5,4-*b*][1,4]oxazin-4-amine **26**

To a stirred solution of **25** (90.0 mg, 0.29 mmol) in anhydrous dioxane (1.7 mL) in a microwave vial were added p-methoxybenzylamine (0.09 mL, 0.65 mmol) and DIPEA (0.09 mL, 0.65 mmol). The resulting mixture was heated at 220 °C for 2 h under microwave irradiation. The reaction mixture was diluted with DCM (10 mL) and washed with sat. aq. NH_4_Cl (10 mL). The aqueous layer was extracted with DCM (3 × 10 mL). The combined organic layers were dried (MgSO_4_), filtered and concentrated *in vacuo*. The crude product was purified by flash chromatography (silica-gel column, 0-5% MeOH in DCM) to afford the title compound as a light brown solid (36.0 mg, 0.087 mmol, 28%). %). mp = 168.3 – 171.5 °C; UV λ_max_ (EtOH) nm: 228; IR ν_max_ cm^-1^: 3430, 2931, 2781, 2735, 2577, 2425, 1604, 1515, 1476, 1437, 1369, 1349; ^1^H-NMR (500 MHz, CDCl_3_) δ_H_ 1.35-1.43 (2H, m, CH_2_-C*H*_2_-CH*-cyclopentane*), 1.54-1.72 (6H, m, N-CH_2_-C*H_2_*, C*H_2_-cyclopentane* and C*H_2_-cyclopentane*), 1.77-1.86 (2H, m, NH-CH_2_-C*H_2_*), 1.96-2.05 (2H, m CH_2_-C*H*_2_-CH*-cyclopentane*), 2.84 (2H, t, *J* = 4.3 Hz, NH-C*H_2_*), 3.38 (2H, t, *J* = 4.3 Hz, N-C*H_2_*-CH_2_-O), 3.40-3.46 (2H, m, N-C*H_2_*), 3.72 (3H, s, C*H_3_*-O), 3.95 (2H, s, C*H_2_*-*benzyl*), 4.11 (2H, t, *J* = 4.3 Hz, C*H_2_*-O), 4.27 (1H, sex, *J* = 7.3 Hz, C*H-cyclopentane*), 4.47-4.55 (1H, br m, CH_2_-N*H*), 4.60 (1H, d, *J* = 7.3 Hz, CH-N*H*), 6.85 (2H, d, *J* = 8.7 Hz, *H*-3 and *H*-5), 7.46 (2H, d, *J* = 8.7 Hz, *H*-3 and *H*-5), 7.77 (1H, s, *H-pyrimidine*); ^13^C-NMR (125 MHz, CDCl_3_) δ_C_ 22.8 (NH-CH_2_-*C*H_2_), 23.5 (*C*H_2_*-cyclopentane* and *C*H_2_*-cyclopentane*), 24.4 (N-CH_2_-*C*H_2_), 33.5 (CH_2_-*C*H_2_-CH-*C*H_2_*-cyclopentane*), 45.6 (NH-*C*H_2_), 45.9 (N-*C*H_2_-CH_2_-O), 46.3 (N-*C*H_2_), 50.1 (*C*H_2_-*benzyl*), 52.1 (*C*H*-cyclopentane*), 55.1 (O-*C*H_3_), 63.6 (*C*H_2_-O), 114.3 (*C*-3 and *C*-5), 119.8 (*C*-O*-pyrimidine*), 122.1 (*C*-1), 147.2 (*C*-N), 149.7 (*C*H*-pyrimidine*), 152.5 (*C*-NH), 131.6 (*C*-2 and *C*-6), 160.2 (*C*-4); MS (ES^+^) *m/z* 412.3 [M+H]^+^ and *m/z* 292.3 [M-PMB+H]^+^.

6-chloro-N-cyclopentyl-5-(2-(piperidin-1-yl)ethoxy)pyrimidin-4-amine **27**

To a stirred suspension of **4** (239.0 mg, 1.12 mmol) and K_2_CO_3_ (387.0 mg, 2.80 mmol) in MeCN (6.2 mL) and DMF (1.2 mL) was added 1-(2-chloroethyl)piperidine hydrochloride (309.0 mg, 1.68 mmol). The reaction mixture was heated at 80 °C overnight, filtered and the filtrate concentrated *in vacuo*. The crude product was purified by flash chromatography (silica-gel column, 0-10% MeOH in DCM) to afford the title compound as a colourless oil (114 mg, 0.35 mmol, 31%). ^1^H-NMR (500 MHz, DMSO-d_6_) δ_H_ 1.35-1.42 (2H, m, C*H*_2_-*piperidine* and C*H*_2_-*piperidine*), 1.43-1.61 (8H, m, C*H*_2_-*piperidine* and C*H*_2_-*piperidine*, N-C*H*_2_-*piperidine* and N-C*H*_2_-*piperidine*, CH_2_-C*H*_2_-CH*-cyclopentane* and CH_2_-C*H*_2_-CH*-cyclopentane*, C*H*_2_*-cyclopentane* and C*H*_2_*-cyclopentane*), 1.67-1.73 (2H, m, C*H*_2_*-cyclopentane* and C*H*_2_*-cyclopentane*), 1.95-2.02 (2H, m, C*H_2_-piperidine*), 2.35-2.42 (2H, m, N-C*H*_2_-*piperidine* and N-C*H*_2_-*piperidine*), 2.57 (2H, t, *J* = 5.3 Hz, C*H_2_*-O), 4.04 (2H, t, *J* = 5.3 Hz, C*H_2_*-N), 4.30 (1H, sex, *J* = 7.5 Hz, C*H-cyclopentane*), 7.19 (1H, d, *J* = 7.5 Hz, N*H*), 8.05 (1H, s, *H-pyrimidine*); ^13^C-NMR (125 MHz, CDCl_3_) δ_C_ 24.0 (*C*H_2_*-cyclopentane* and *C*H_2_*-cyclopentane*), 24.4 (*C*H_2_*-piperidine*), 25.6 (*C*H_2_*-piperidine* and *C*H_2_*-piperidine*), 32.5 (CH_2_-*C*H_2_-CH-*C*H_2_*-cyclopentane*), 52.6 (*C*H*-cyclopentane*), 54.6 (N-*C*H_2_*-piperidine* and N-*C*H_2_*-piperidine*), 58.1 (*C*H_2_-N), 70.0 (*C*H_2_-O), 134.4 (*C*-O), 147.5 (*C*-N), 153.0 (*C*H*-pyrimidine*), 158.2 (*C*-NH);

N-cyclopentyl-6-(piperidin-1-yl)-5-(vinyloxy)pyrimidin-4-amine **28**

To a stirred suspension of **4** (239.0 mg, 1.12 mmol) and K_2_CO_3_ (387.0 mg, 2.80 mmol) in MeCN (6.2 mL) and DMF (1.2 mL) was added 1-(2-chloroethyl)piperidine hydrochloride (309.0 mg, 1.68 mmol). The reaction mixture was heated at 80 °C overnight, filtered and the filtrate concentrated *in vacuo*. The crude product was purified by flash chromatography (silica-gel column, 0-10% MeOH in DCM) to afford the title compound as a yellow oil (91 mg, 0.31 mmol, 28%). ^1^H-NMR (500 MHz, DMSO-d_6_) δ_H_ 1.40-1.48 (2H, m, CH_2_-C*H*_2_-CH*-cyclopentane* and CH_2_-C*H*_2_-CH*-cyclopentane*), 1.49-1.60 (4H, m, C*H_2_*-*piperidine*, C*H*_2_*-cyclopentane* and C*H*_2_*-cyclopentane*), 1.63-1.71 (2H, m, C*H*_2_*-cyclopentane* and C*H*_2_*-cyclopentane*), 1.72-1.79 (4H, m, C*H_2_*-*piperidine* and C*H_2_*-*piperidine*), 1.88-1.94 (2H, m, CH_2_-C*H*_2_-CH*-cyclopentane* and CH_2_-C*H*_2_-CH*-cyclopentane*), 3.50-3.58 (2H, m, N-C*H*_2_-*piperidine* and N-C*H*_2_-*piperidine*), 4.13 (1H, sex, *J* = 6.9 Hz, C*H-cyclopentane*), 4.87-4.98 (2H, m, N-C*H*_2_-*piperidine* and N-C*H*_2_-*piperidine*), 5.29 (1H, dd, *J* = 8.2 and 2.9 Hz, H_b_), 5.44 (1H, dd, *J* = 15.4 and 2.9 Hz, H_c_), 6.38 (1H, d, *J* = 6.9 Hz, N*H*), 6.64 (1H, dd, *J* = 15.4 and 8.2 Hz, O-C*H_a_*=CH_2_), 7.30 (1H, s, *H-pyrimidine*); ^13^C-NMR (125 MHz, CDCl_3_) δ_C_ 21.5 (*C*H_2_*-piperidine*), 21.7 (*C*H_2_*-piperidine* and *C*H_2_*-piperidine*), 23.8 (*C*H_2_*-cyclopentane* and *C*H_2_*-cyclopentane*), 33.2 (CH_2_-*C*H_2_-CH-*C*H_2_*-cyclopentane*), 52.1 (*C*H*-cyclopentane*), 58.8 (N-*C*H_2_*-piperidine* and N-*C*H_2_*-piperidine*), 110.0 (*C*H_2_-*vinyl*), 132.0 (*C*-O), 135.1 (*C*H*-pyrimidine*), 141.8 (*C*H-*vinyl*), 153.3 (*C*-N), 161.9 (*C*-N);

1. **NMR Spectra**

6-chloro-*N*-cyclopentyl-5-methoxypyrimidin-4-amine **2**

*N*^4^-cyclopentyl-5-methoxy-*N*^6^-(4-methoxybenzyl)pyrimidine-4,6-diamine **3a**

*N*^4^-cyclopentyl-5-methoxypyrimidine-4,6-diamine **3b**

4-chloro-6-(cyclopentylamino)pyrimidin-5-ol **4**

6-chloro-*N*-cyclopentyl-5-isopropoxypyrimidin-4-amine **5**

*N*^4^-cyclopentyl-5-isopropoxy-*N*^6^-(4-methoxybenzyl)pyrimidine-4,6-diamine **6a**

*N*^4^-cyclopentyl-5-isopropoxypyrimidine-4,6-diamine **6b**

5-(4-((*tert*-butyldiphenylsilyl)oxy)butoxy)-6-chloro-*N*-cyclopentylpyrimidin-4-amine **7**

5-((5-((*tert*-butyldiphenylsilyl)oxy)pentyl)oxy)-6-chloro-*N*-cyclopentylpyrimidin-4-amine **8**

5-((6-((*tert*-butyldiphenylsilyl)oxy)hexyl)oxy)-6-chloro-*N*-cyclopentylpyrimidin-4-amine **9**

5-(4-((*tert*-butyldiphenylsilyl)oxy)butoxy)-*N*^4^-cyclopentyl-*N*^6^-(2,4-dimethoxybenzyl)pyrimidine-4,6-diamine **10**

5-((5-((*tert*-butyldiphenylsilyl)oxy)pentyl)oxy)-*N*^4^-cyclopentyl-*N*^6^-(2,4-dimethoxybenzyl)pyrimidine-4,6-diamine **11**

5-((6-((*tert*-butyldiphenylsilyl)oxy)hexyl)oxy)-*N*^4^-cyclopentyl-*N*^6^-(2,4-dimethoxybenzyl)pyrimidine-4,6-diamine **12**

4-((4-amino-6-(cyclopentylamino)pyrimidin-5-yl)oxy)butan-1-ol **13**

5-((4-amino-6-(cyclopentylamino)pyrimidin-5-yl)oxy)pentan-1-ol **14**

6-((4-amino-6-(cyclopentylamino)pyrimidin-5-yl)oxy)hexan-1-ol **15**

6-chloro-*N*-cyclopentyl-5-(2-(dimethylamino)ethoxy)pyrimidin-4-amine **16**

*N*-cyclopentyl-8-methyl-7,8-dihydro-6*H*-pyrimido[5,4-*b*][1,4]oxazin-4-amine **17**

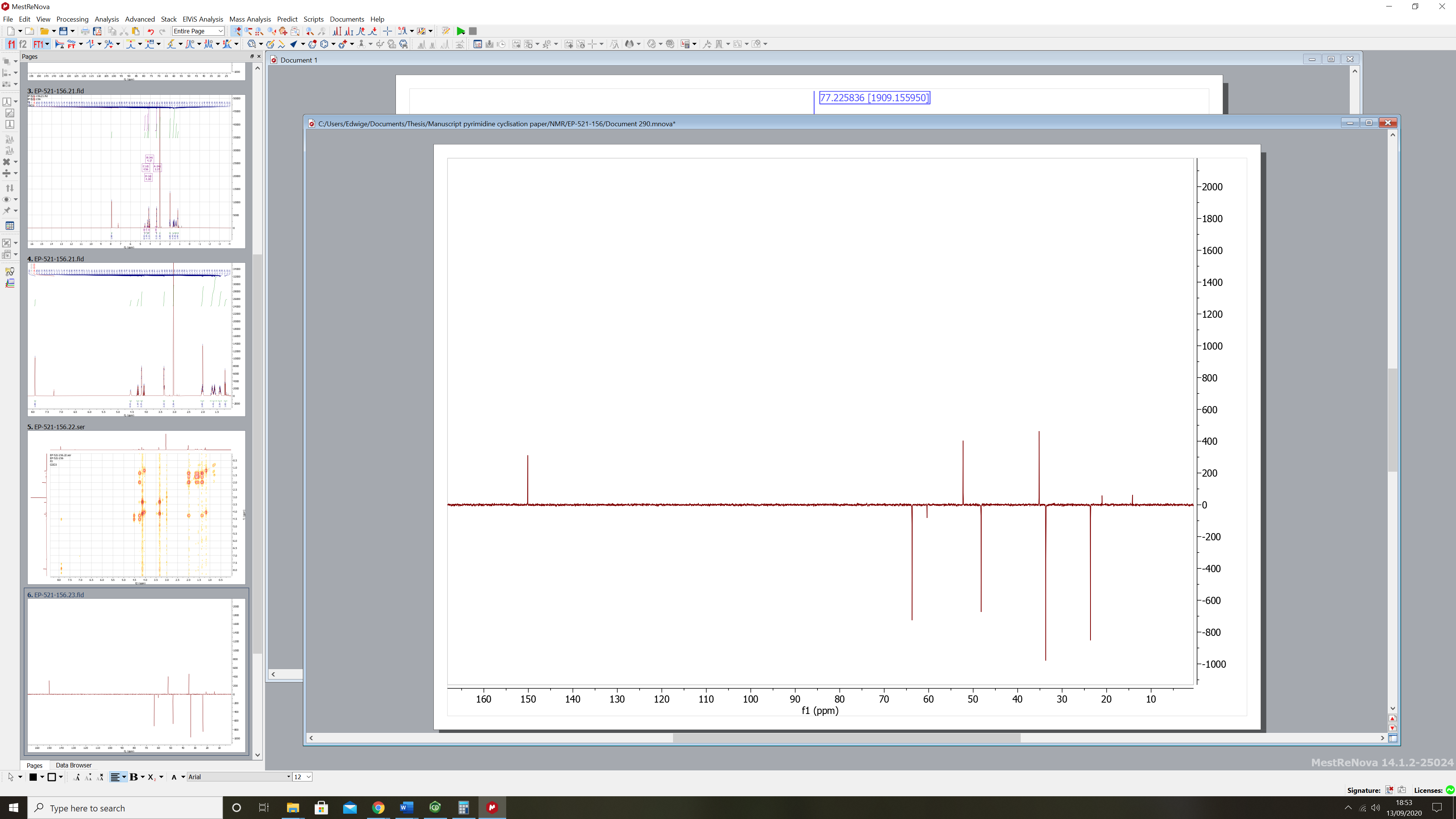


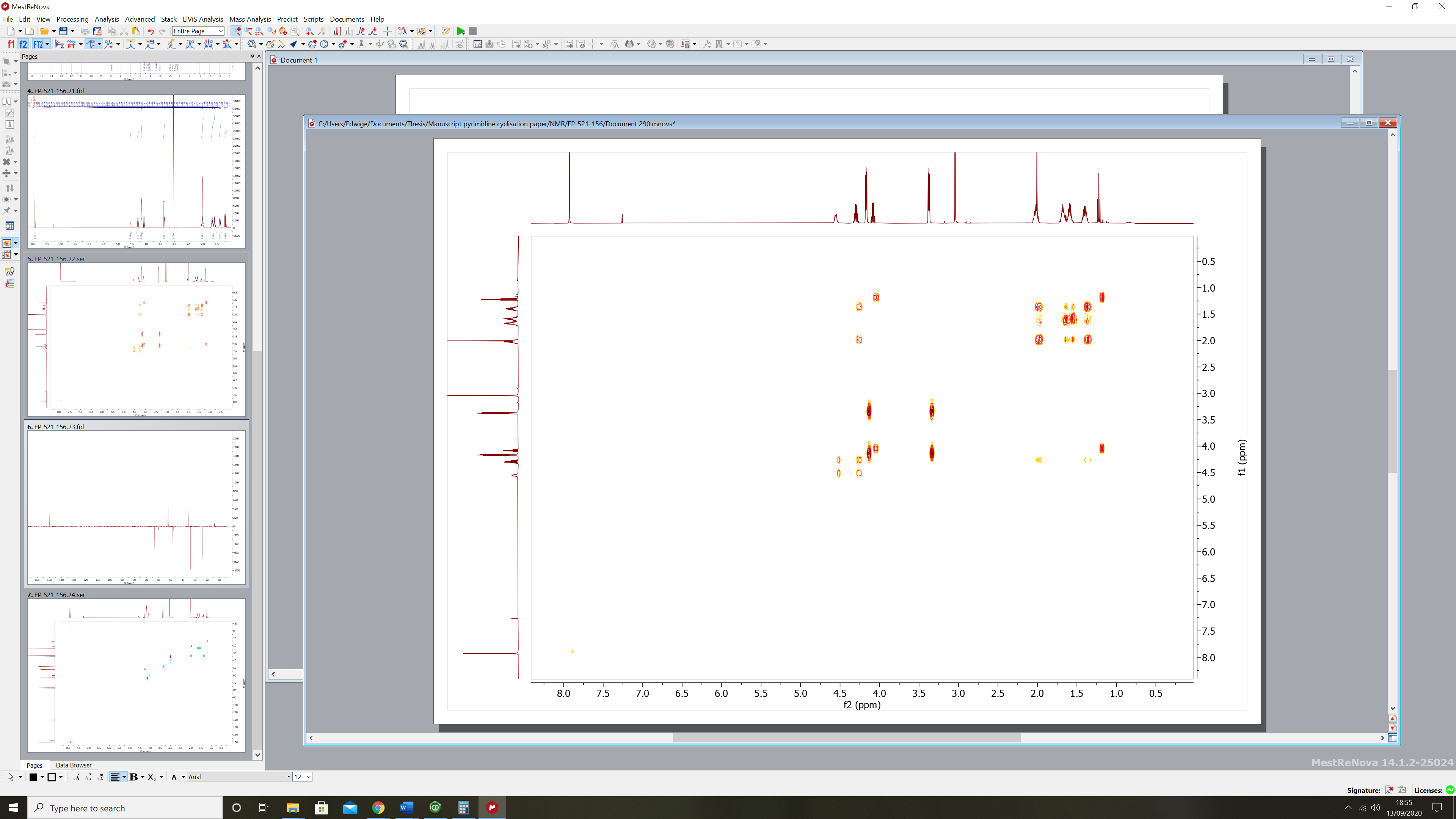


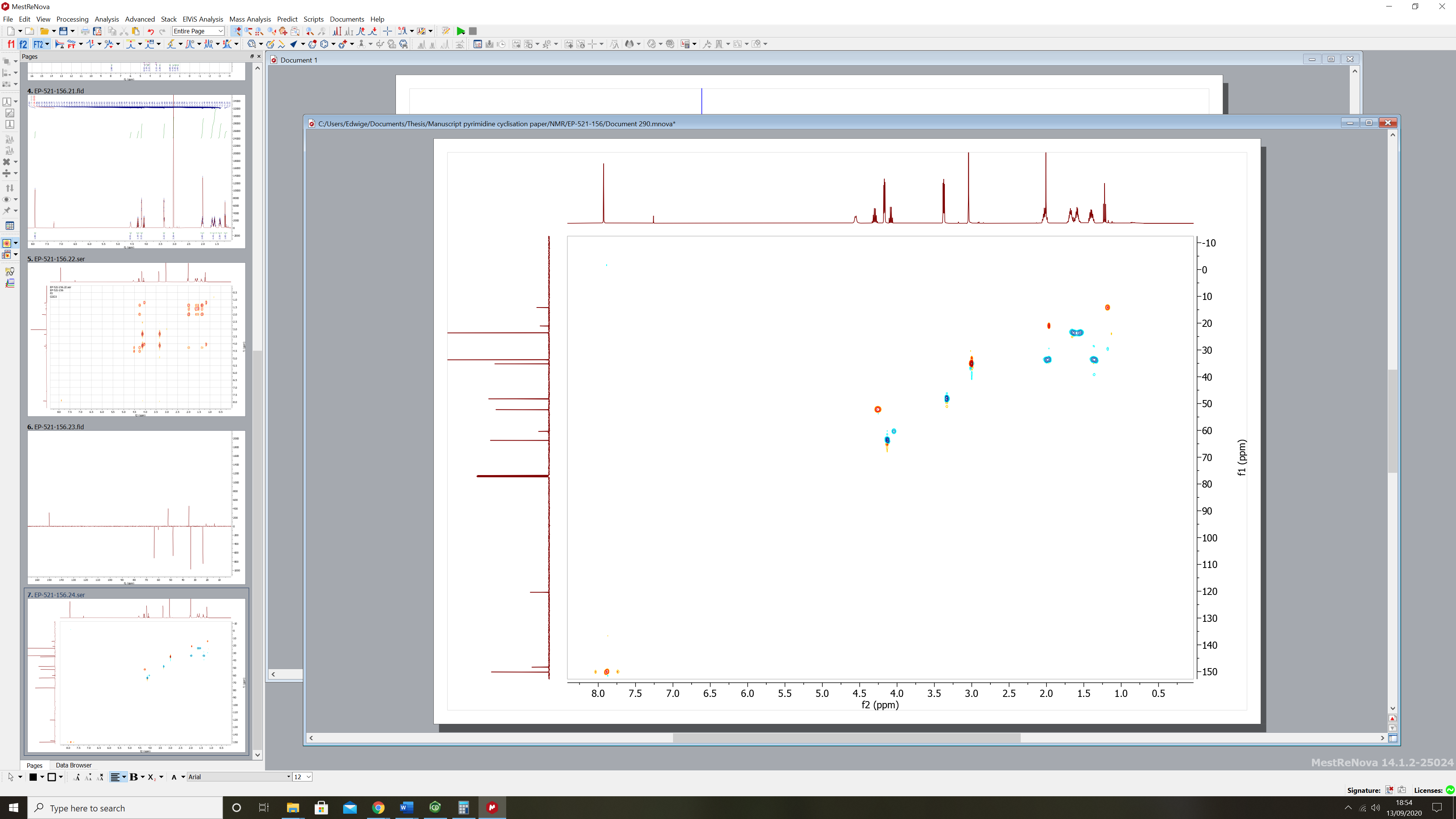


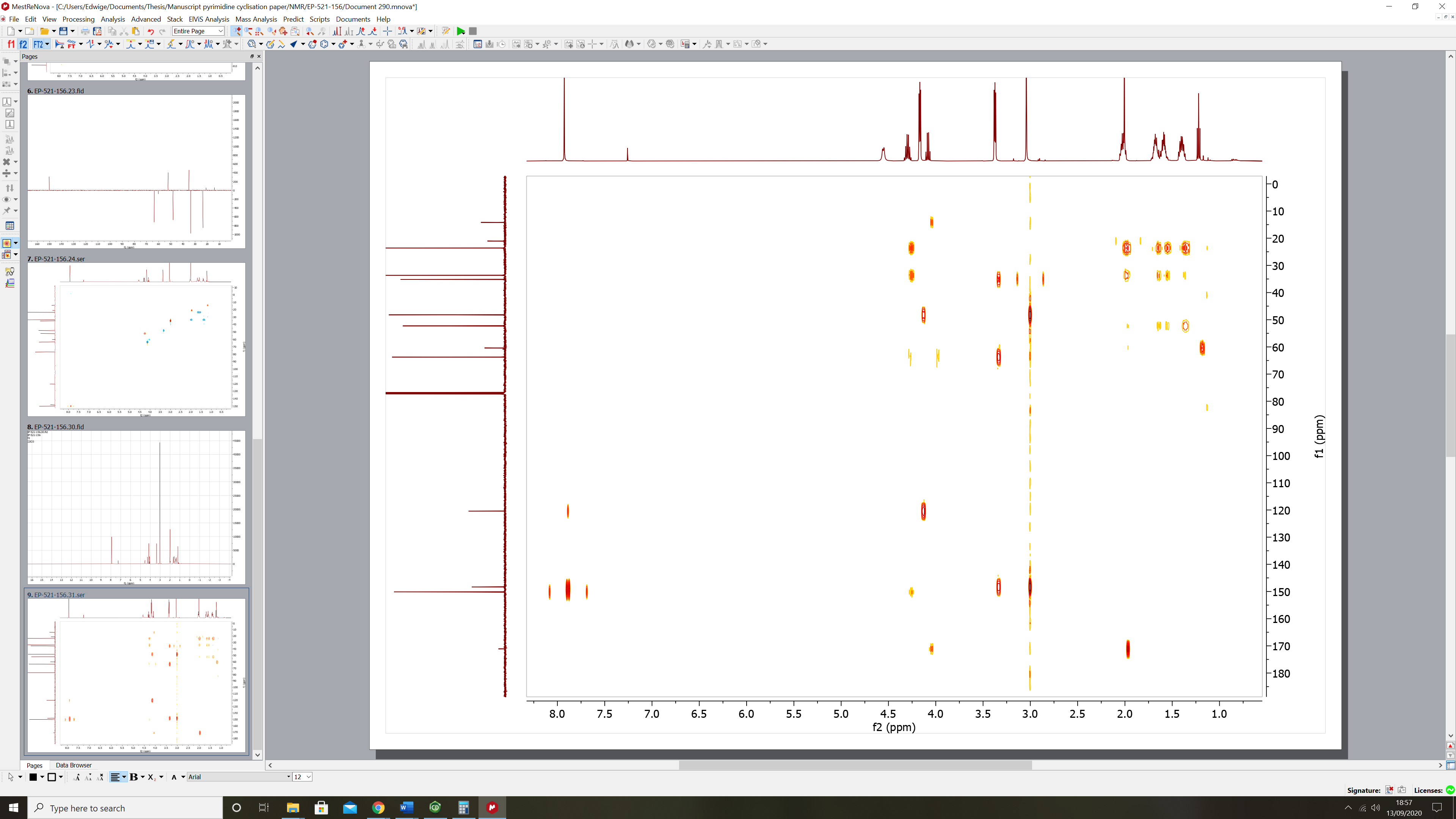


4-(cyclopentylamino)-6-(dimethyliminio)-1-vinyl-1,6-dihydropyrimidin-5-olate **18**

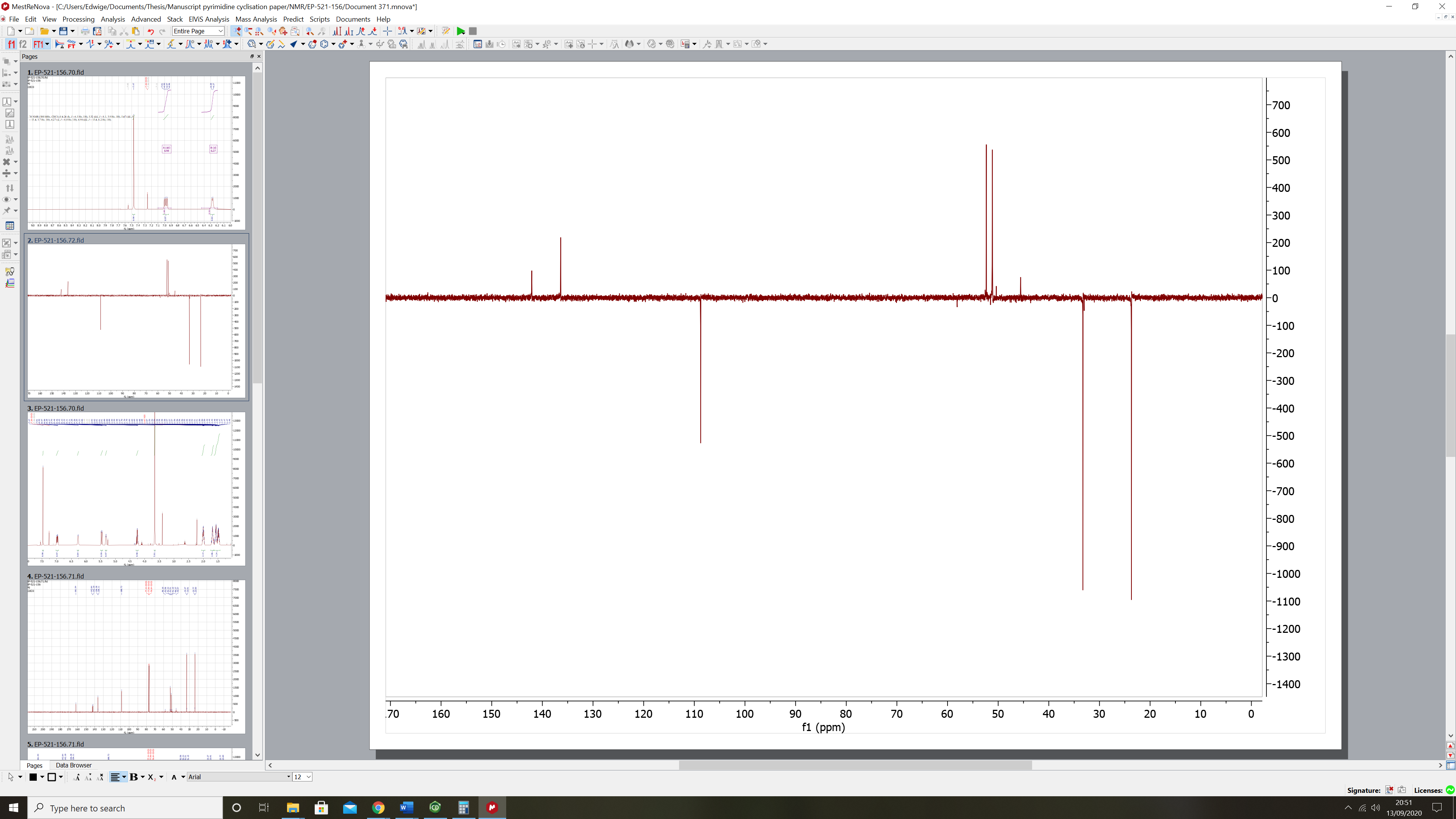


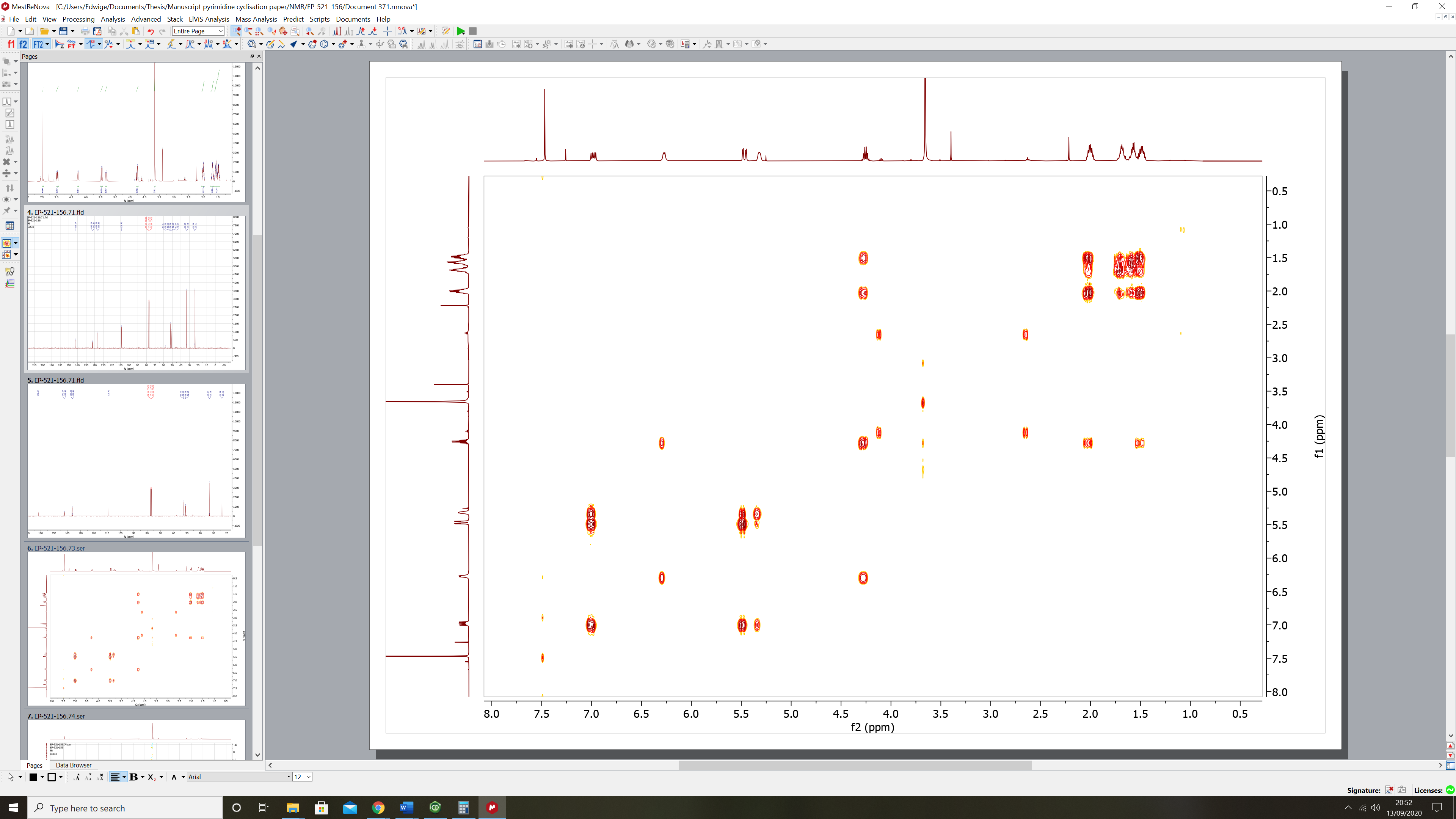


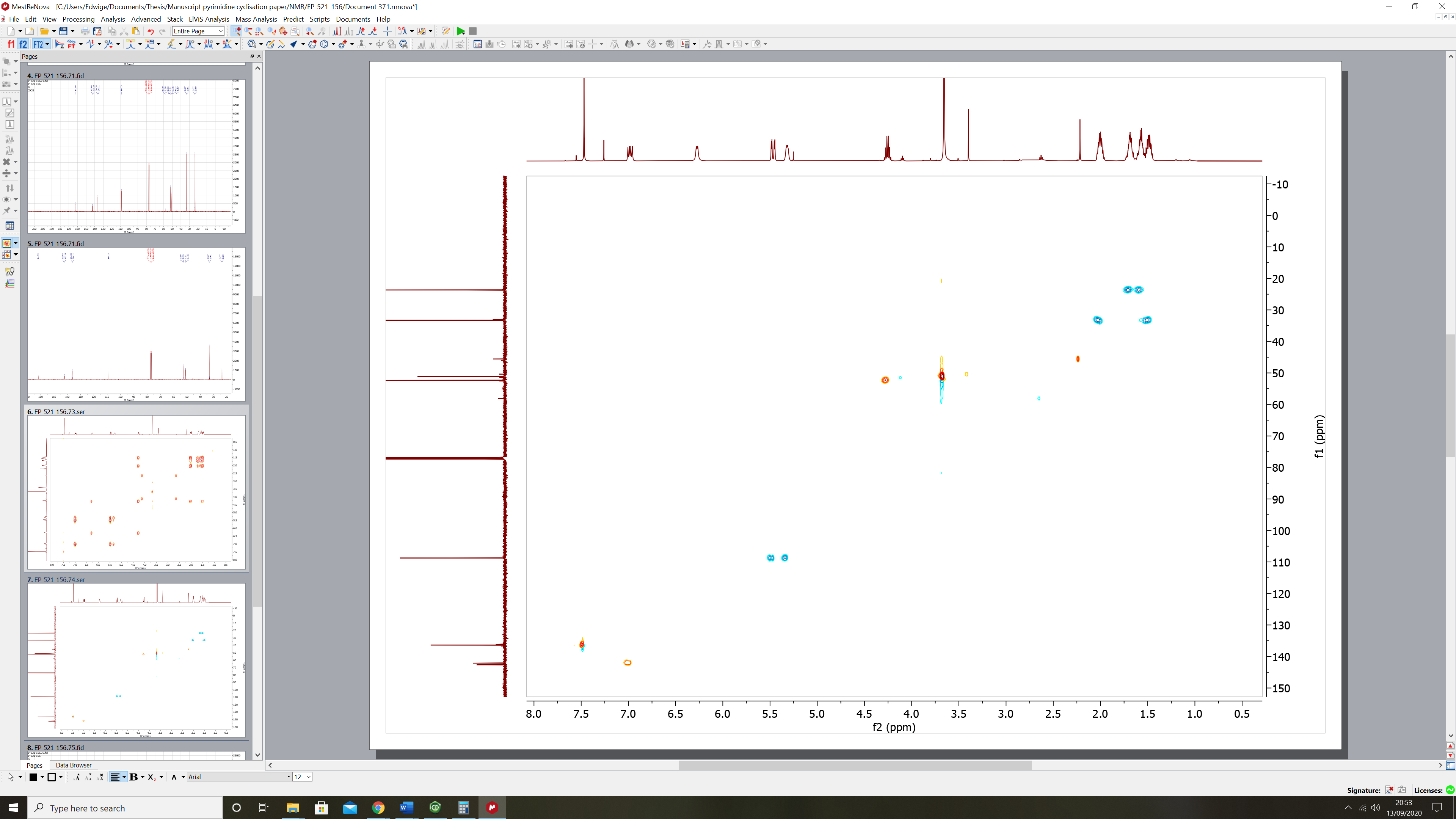


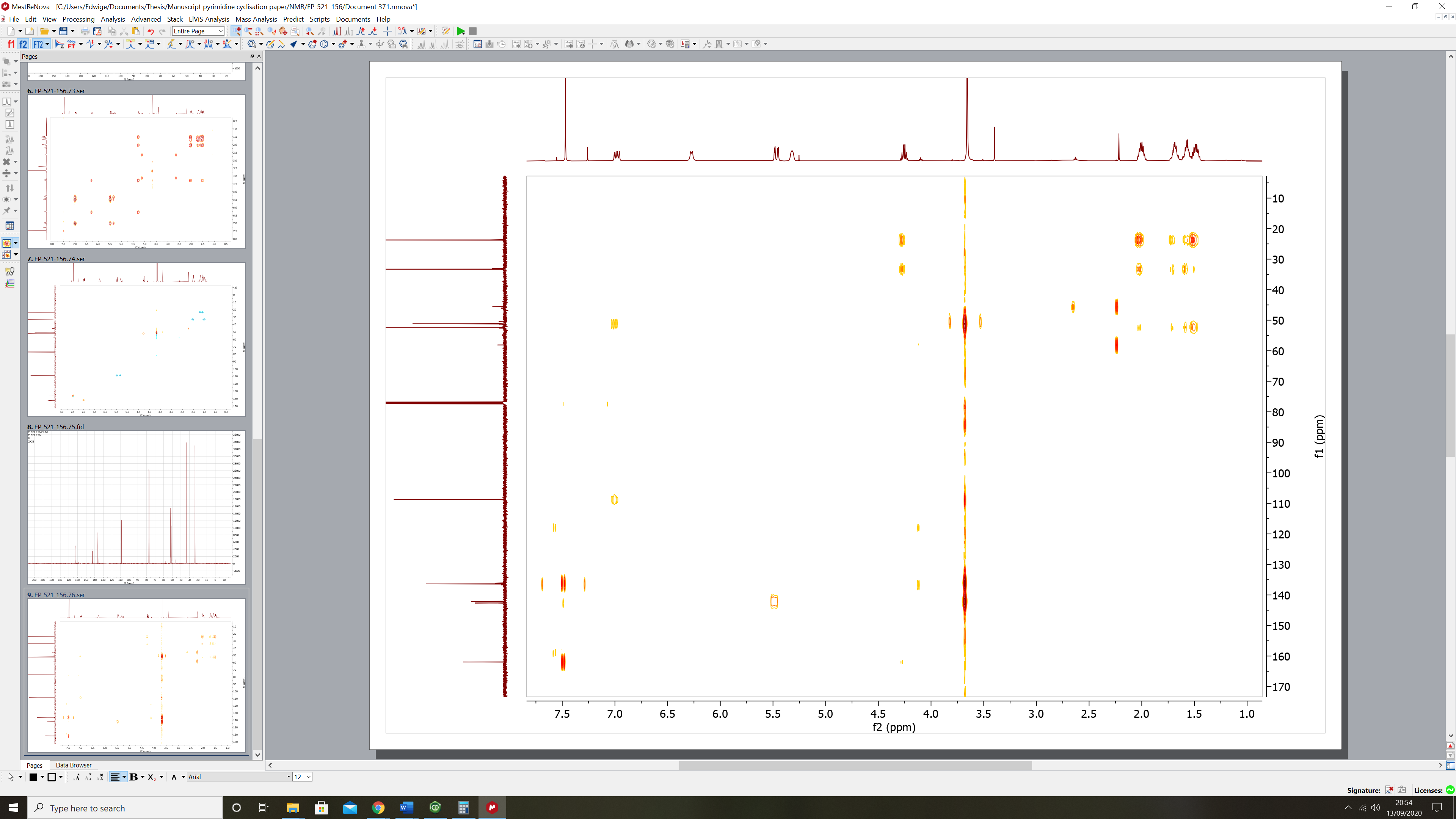


-chloro-*N*-cyclopentyl-5-(vinyloxy)pyrimidin-4-amine **19**

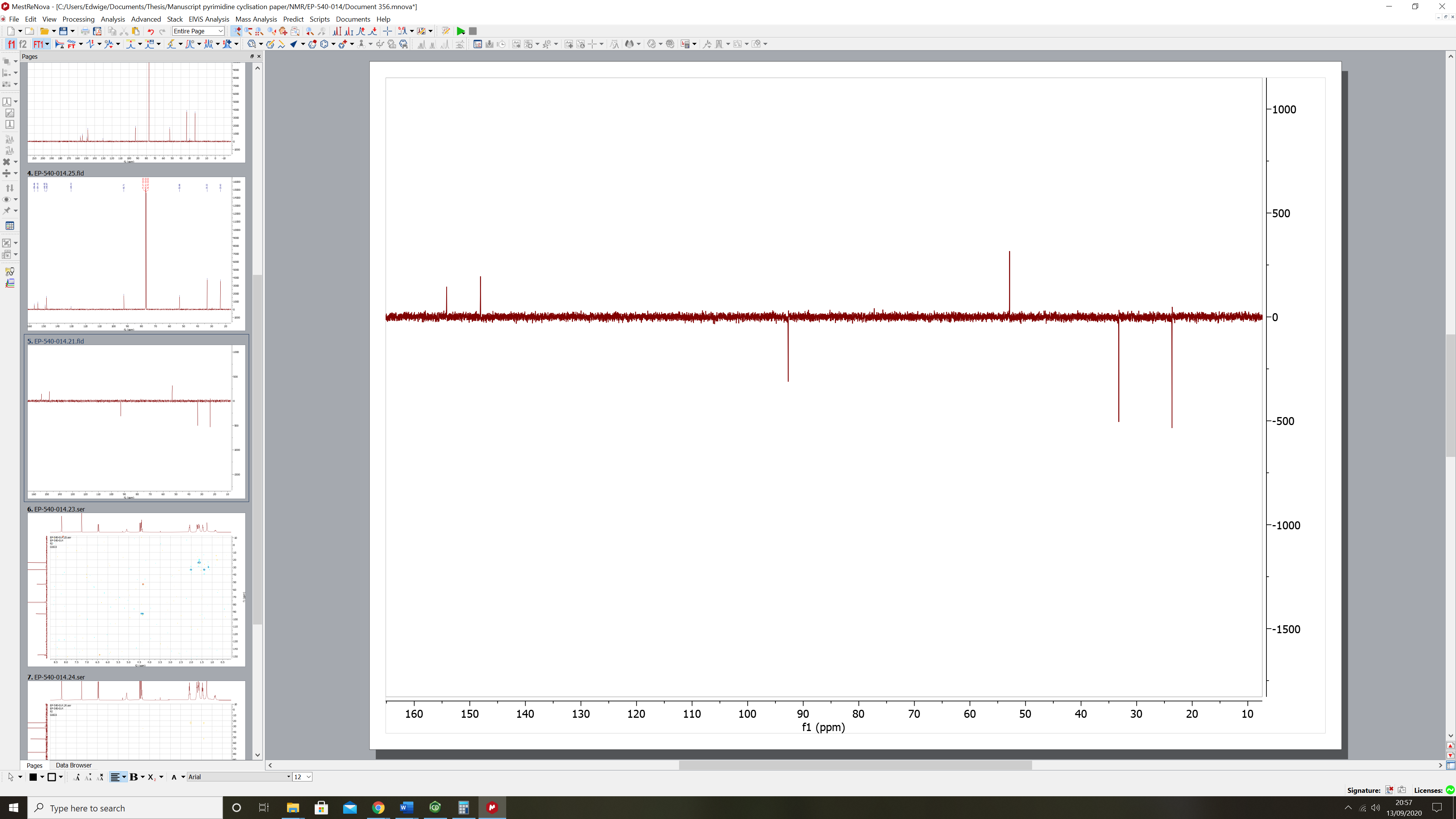


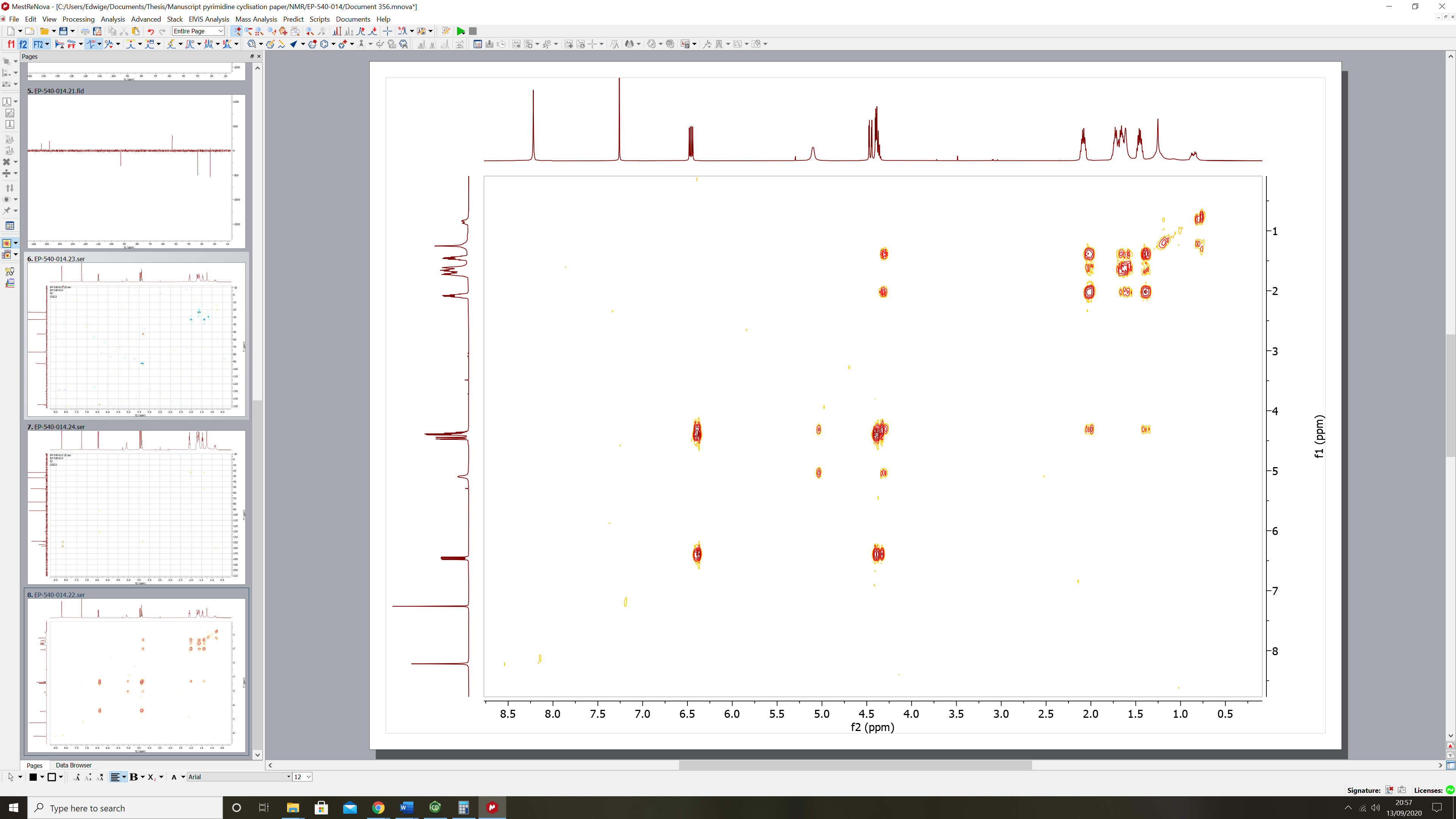


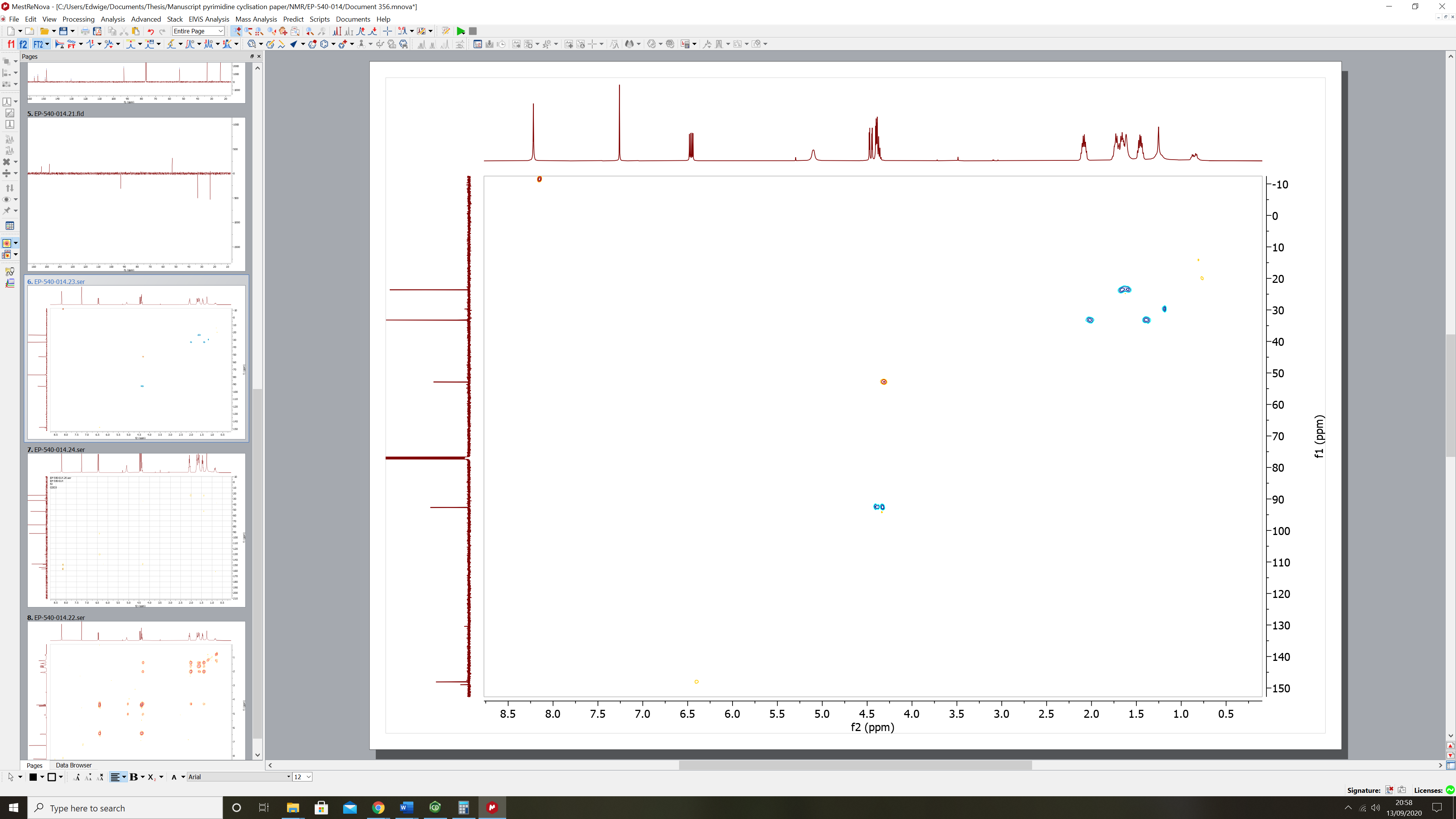


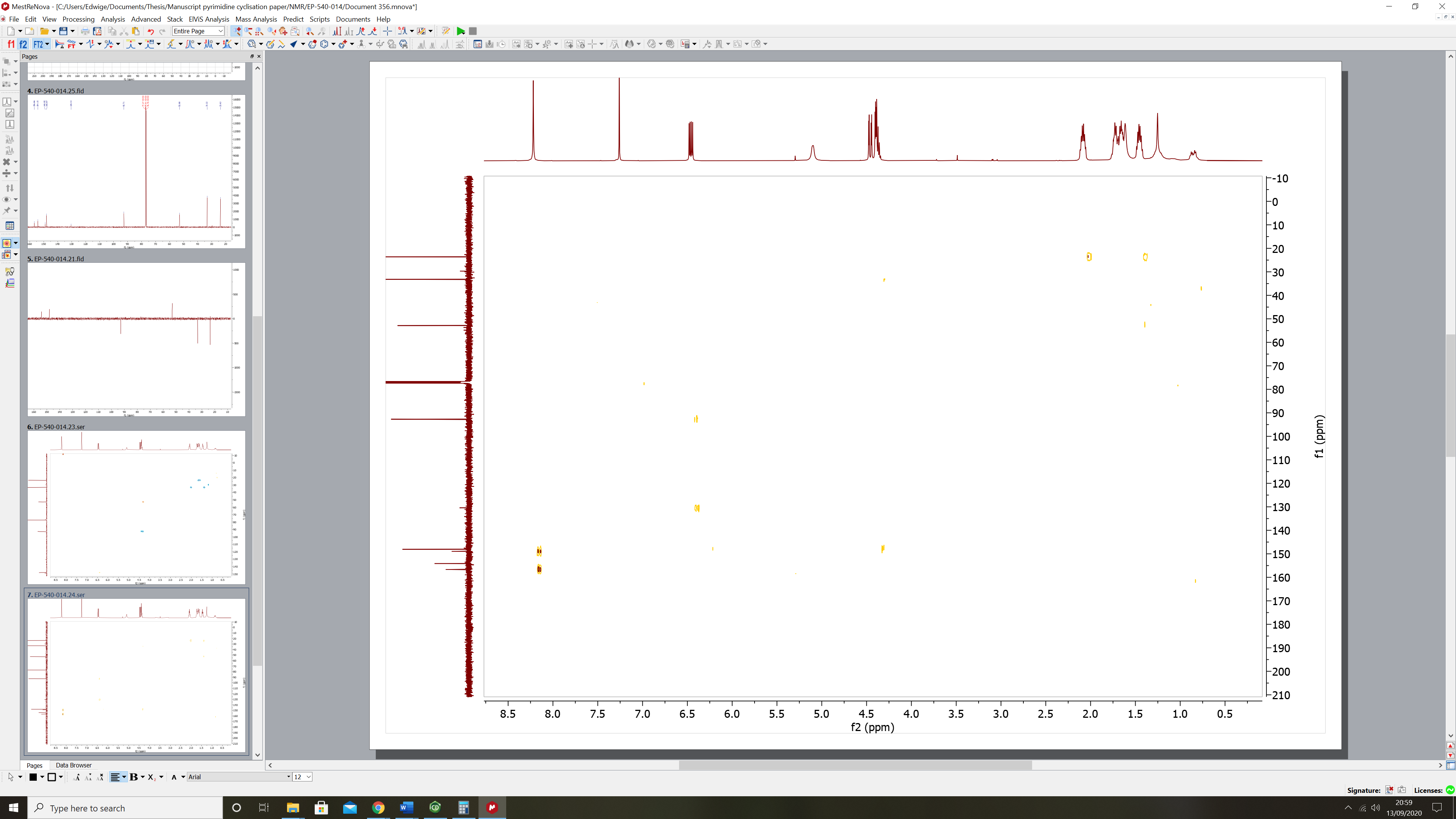


*N*^4^-cyclopentyl-*N*^6^,*N*^6^-dimethyl-5-(vinyloxy)pyrimidine-4,6-diamine **20**

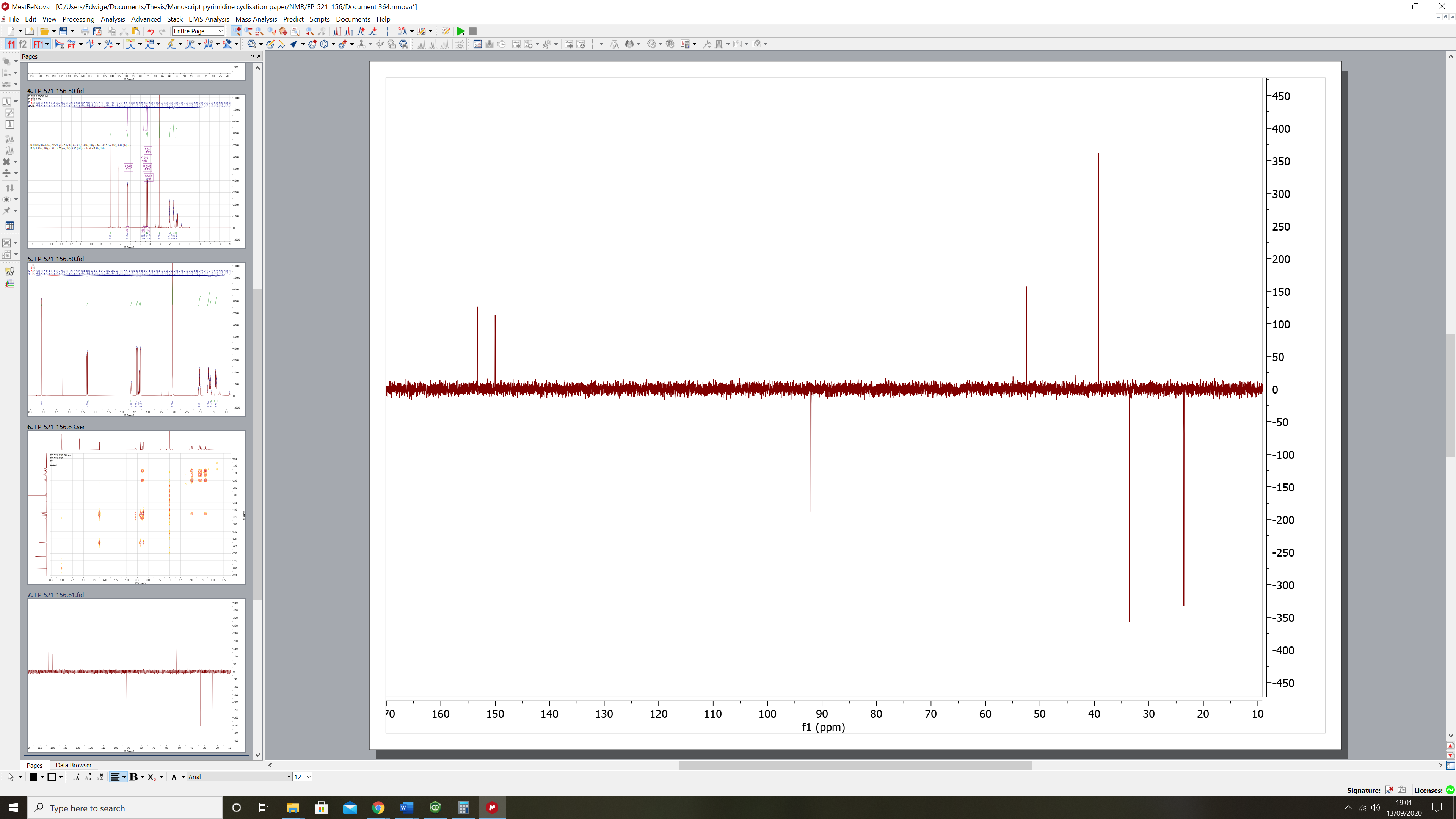


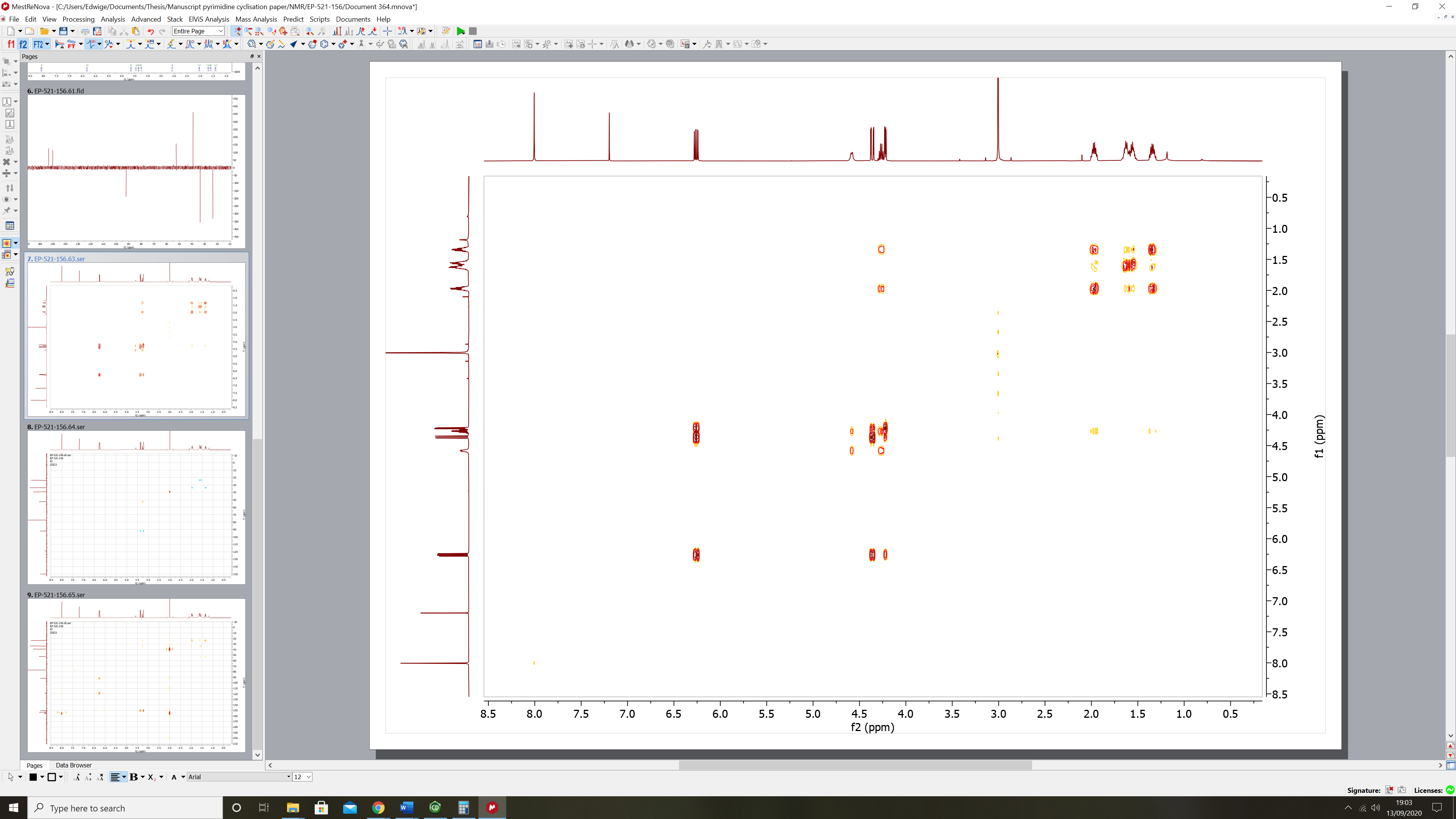


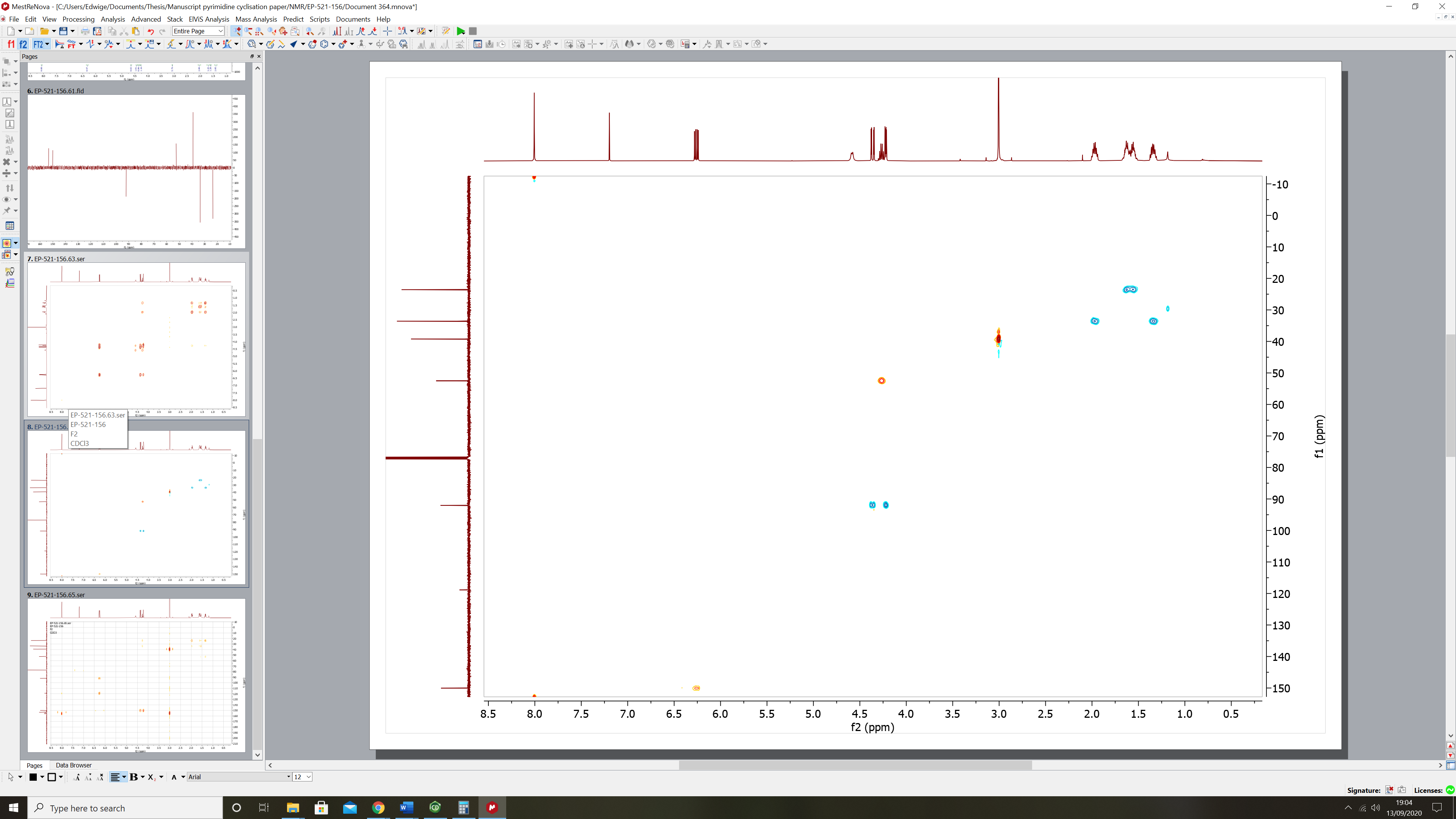


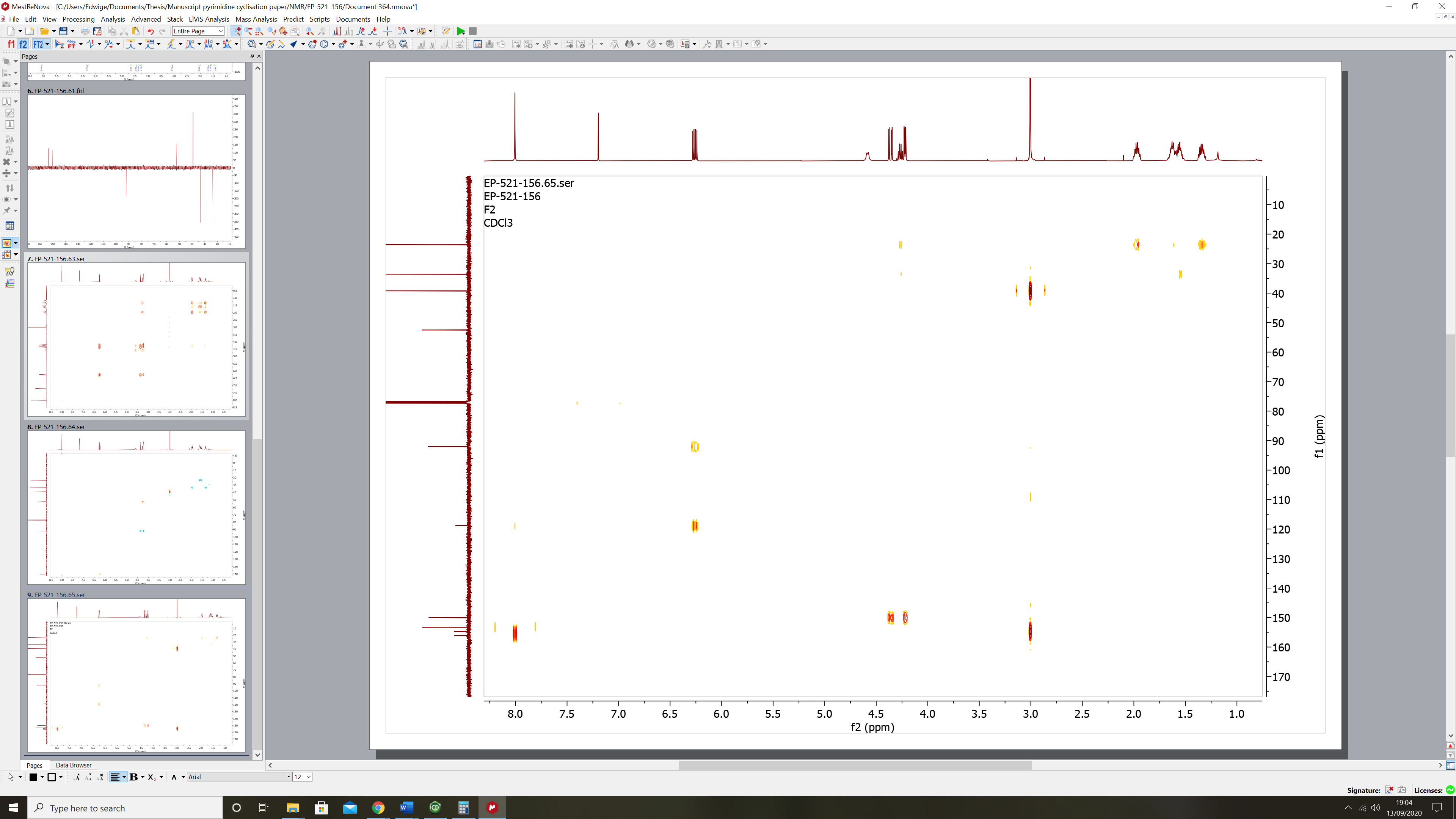


6-chloro-*N*-cyclopentyl-5-(3-(dimethylamino)propoxy)pyrimidin-4-amine **21**

*N*^4^-cyclopentyl-5-(3-(dimethylamino)propoxy)-*N*^6^-(4-methoxybenzyl)pyrimidine-4,6-diamine **22**

*N*-cyclopentyl-9-methyl-6,7,8,9-tetrahydropyrimido[5,4-*b*][1,4]oxazepin-4-amine **23**

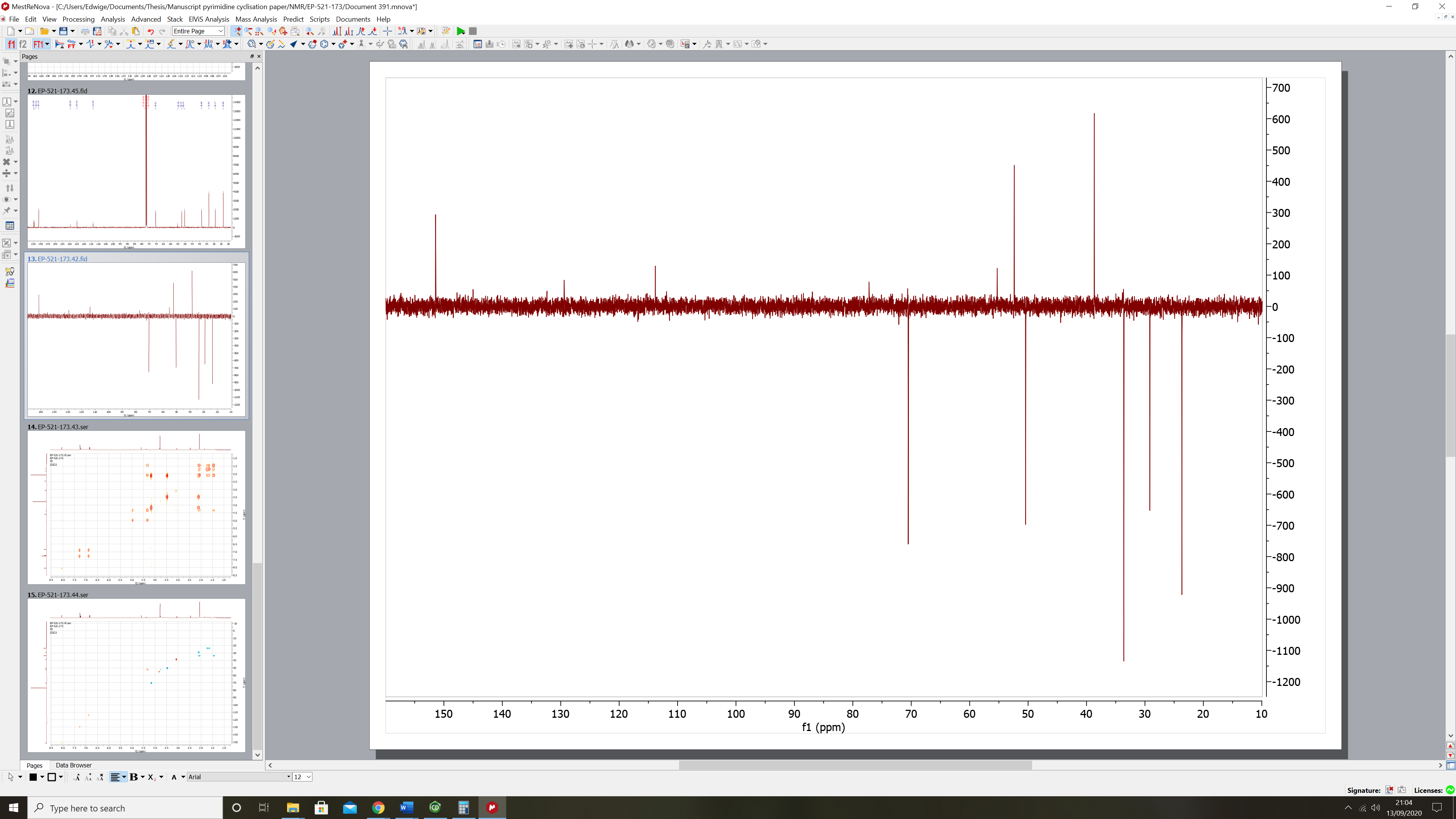


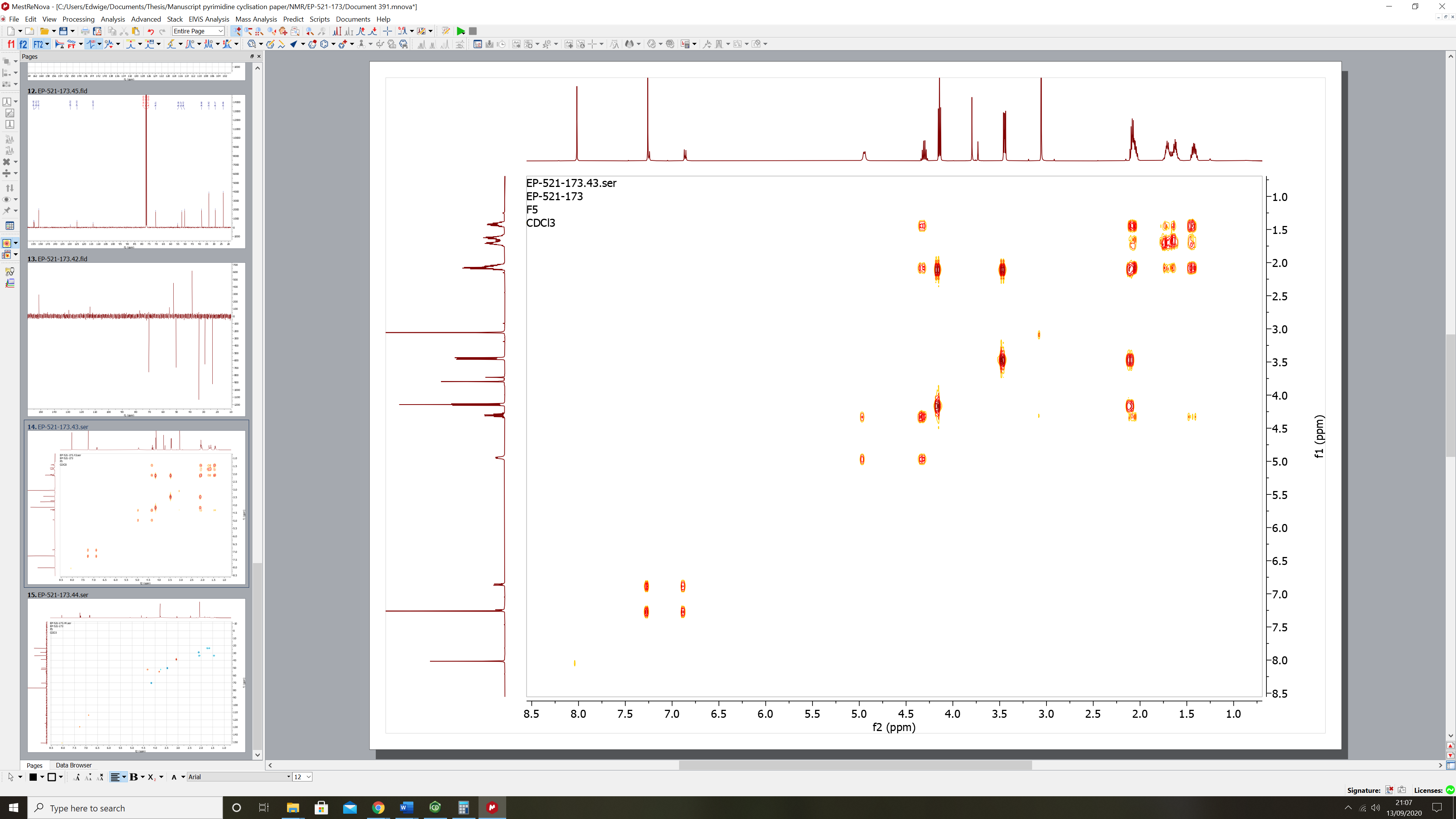


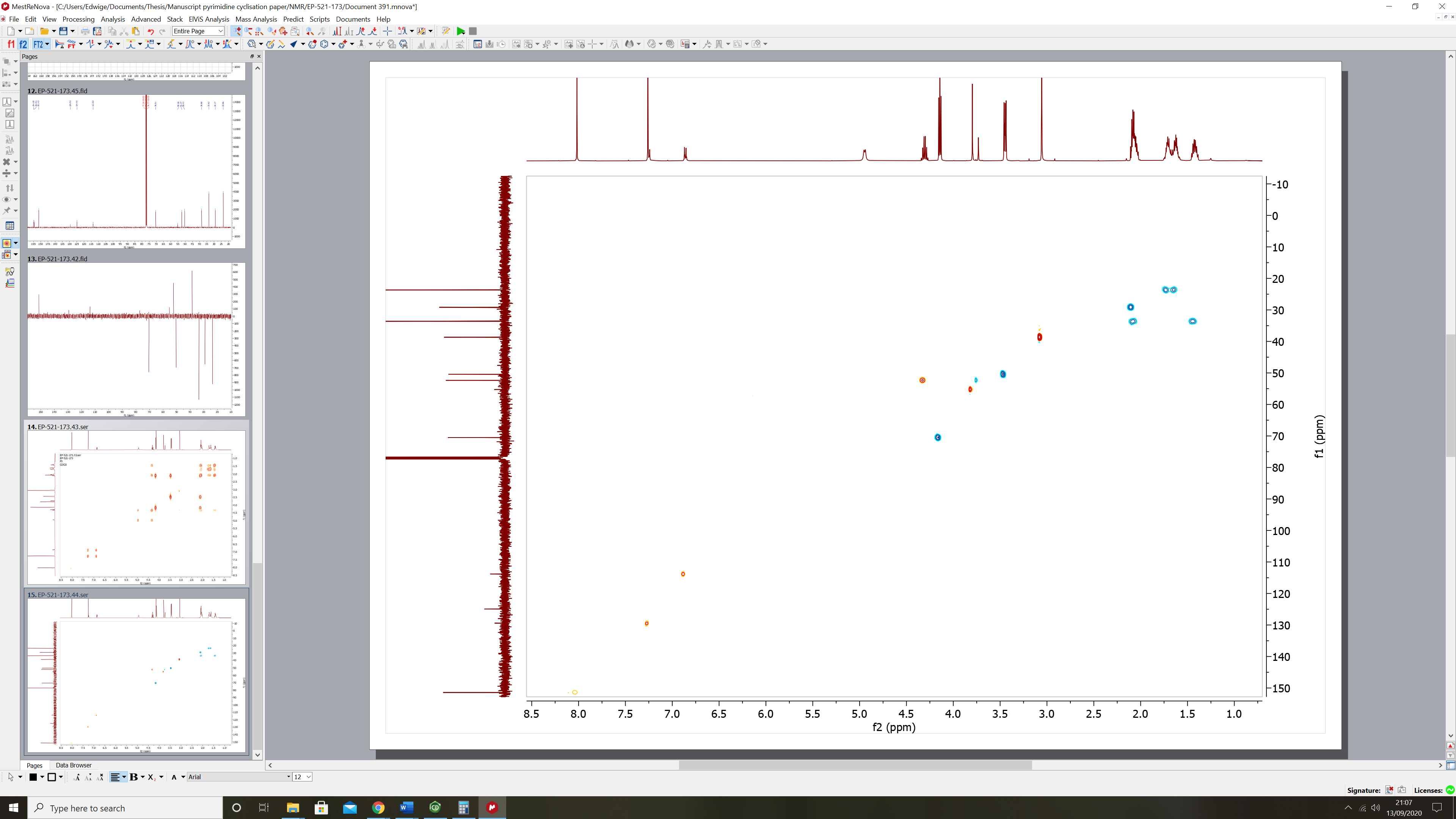


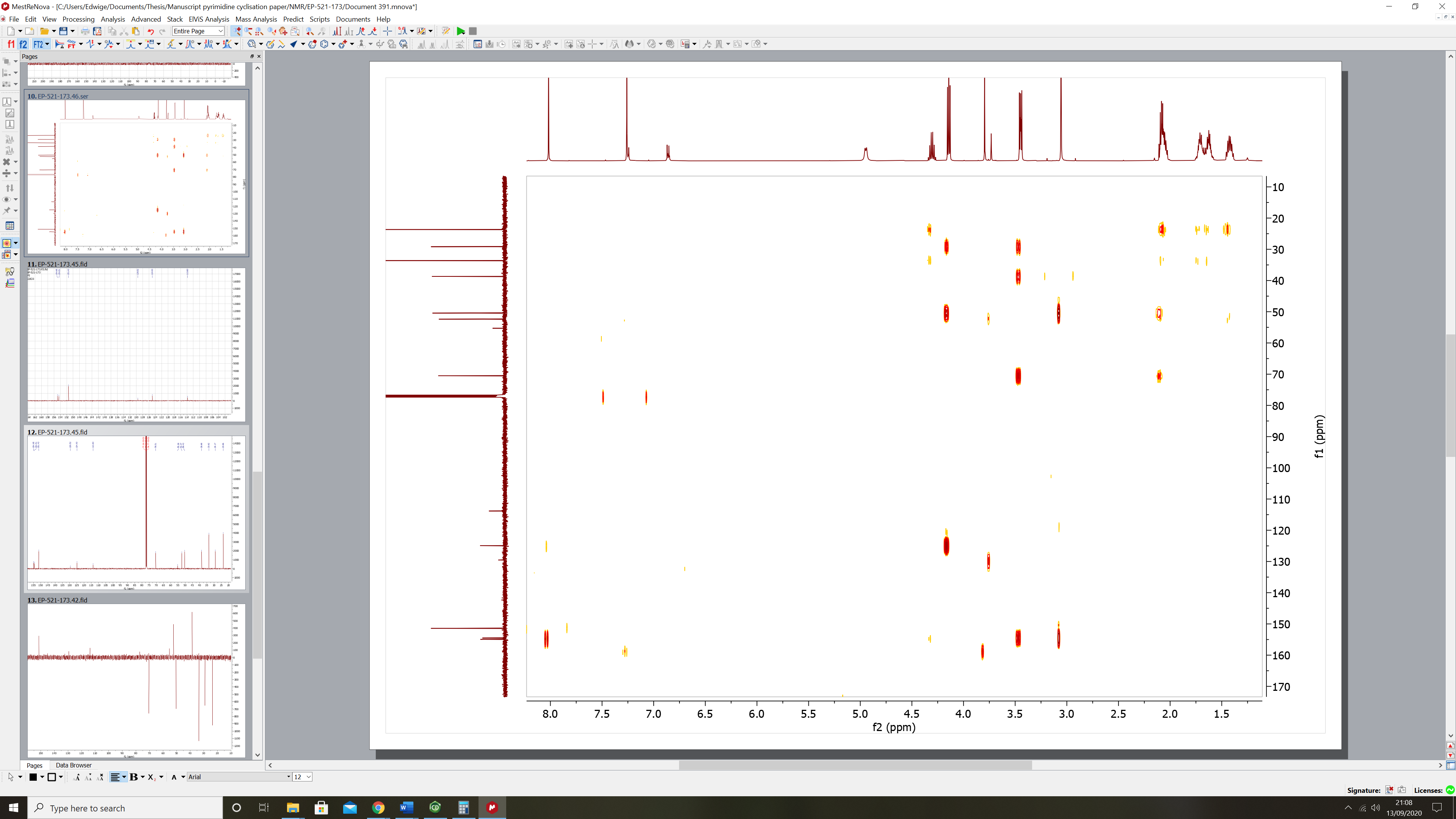


*tert*-butyl 4-(2-((4-chloro-6-(cyclopentylamino)pyrimidin-5-yl)oxy)ethyl)piperidine-1-carboxylate **24**

4-(cyclopentylamino)-6,7-dihydrospiro[pyrimido[5,4-*b*][1,4]oxazine-8,1'-pyrrolidin]-8-ium chloride **25**


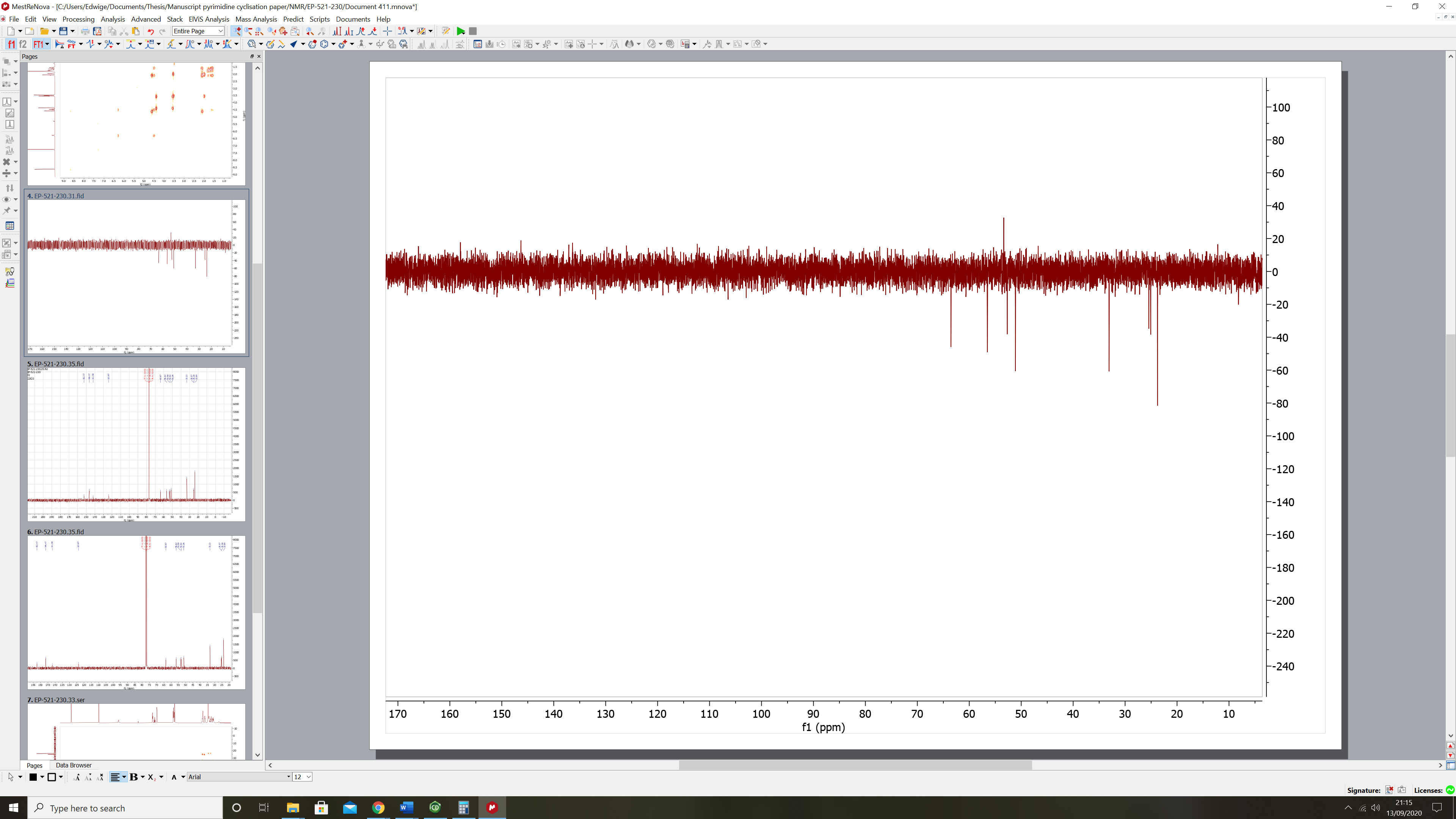


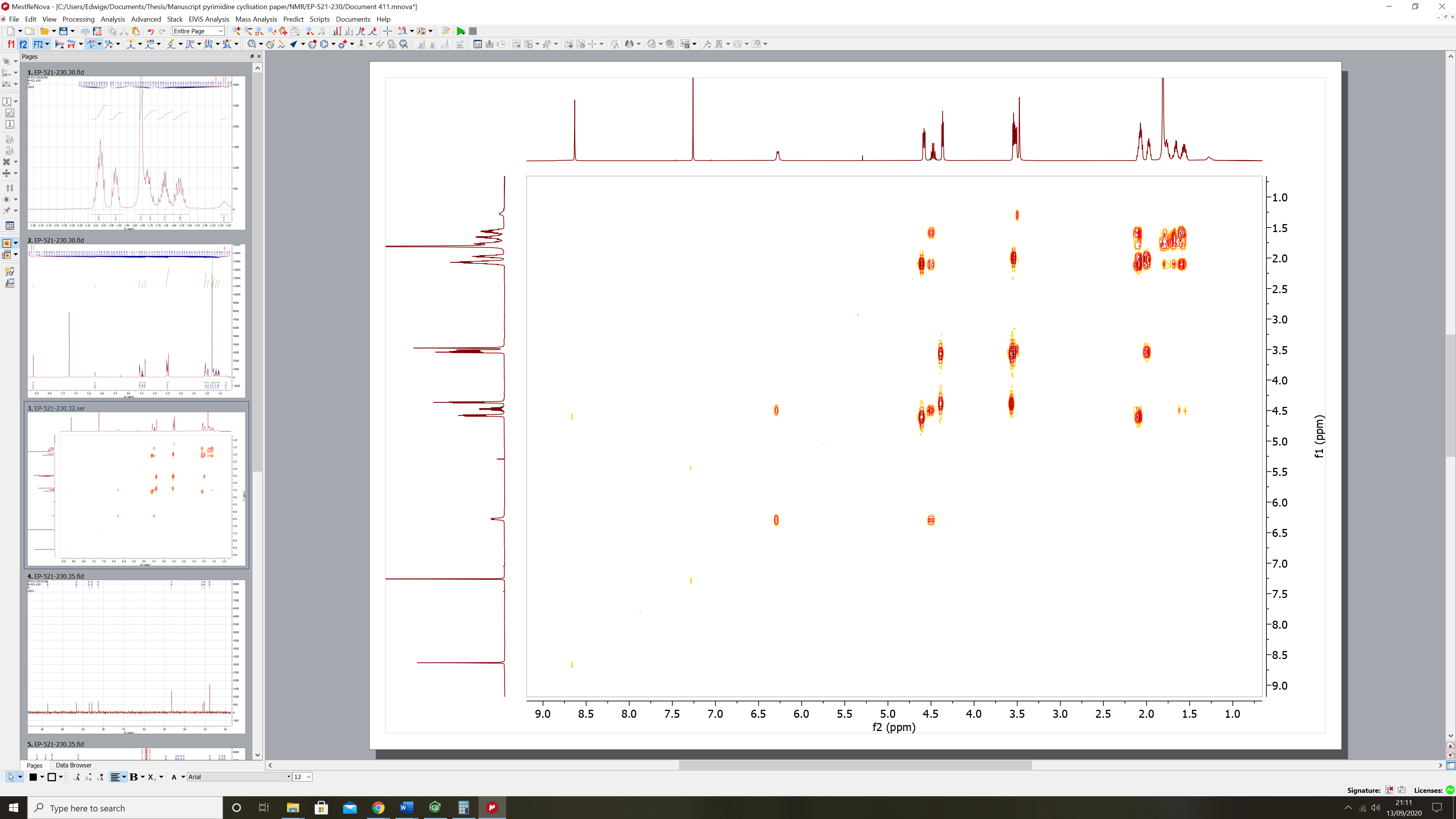


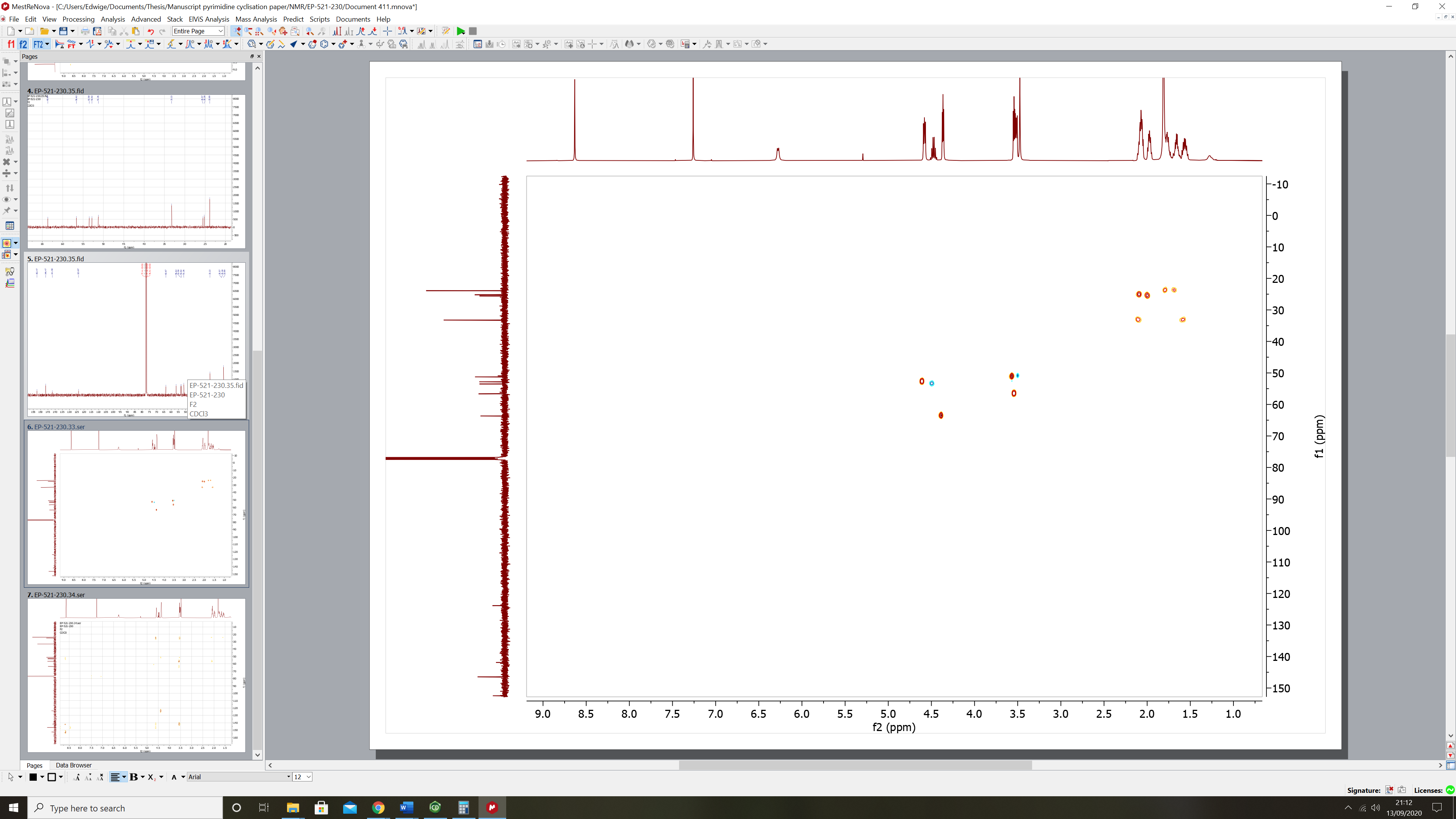


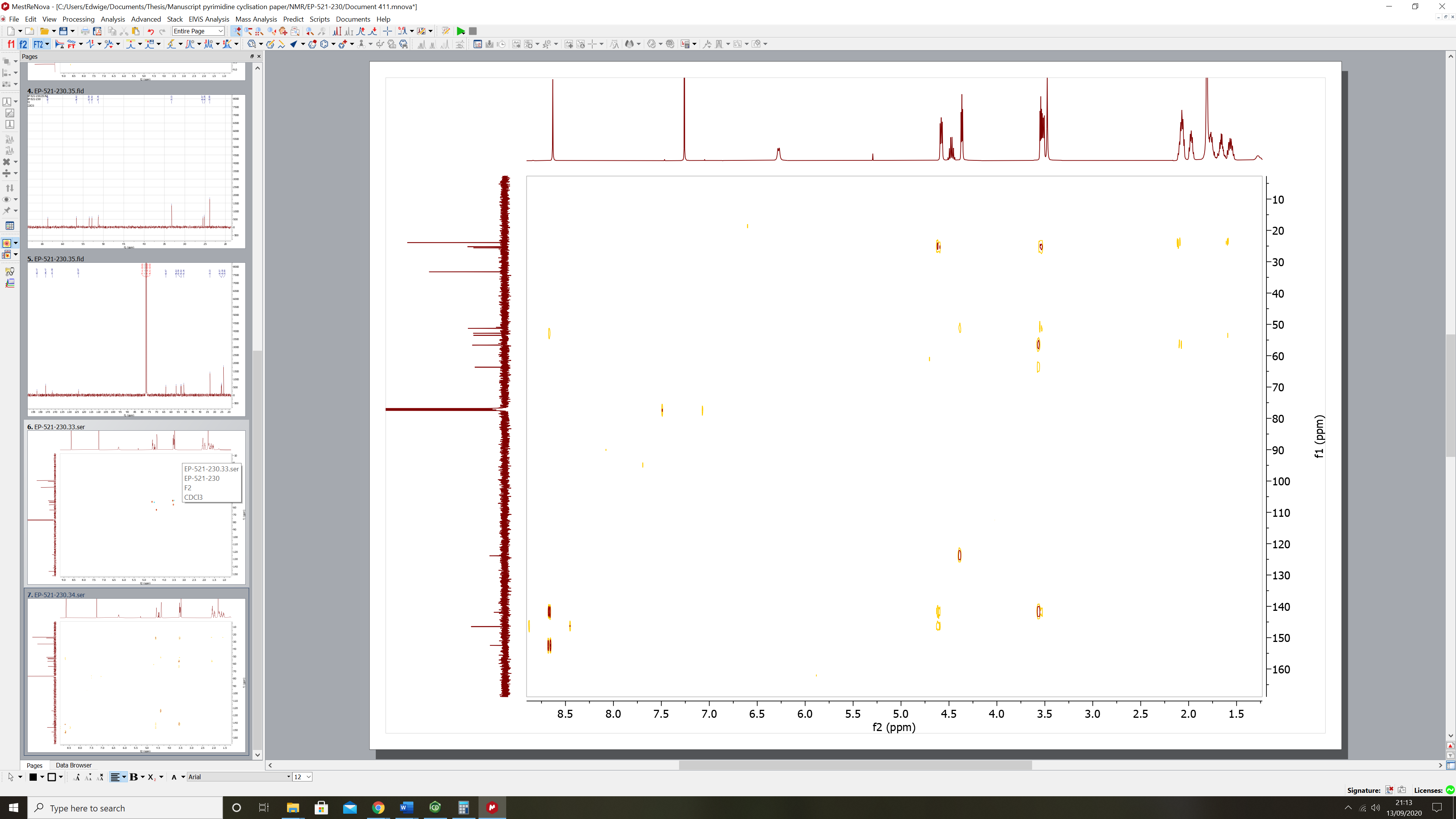


*N*-cyclopentyl-8-(4-((4-methoxybenzyl)amino)butyl)-7,8-dihydro-6*H*-pyrimido[5,4-*b*][1,4]oxazin-4-amine **26**

6-chloro-N-cyclopentyl-5-(2-(piperidin-1-yl)ethoxy)pyrimidin-4-amine **27**


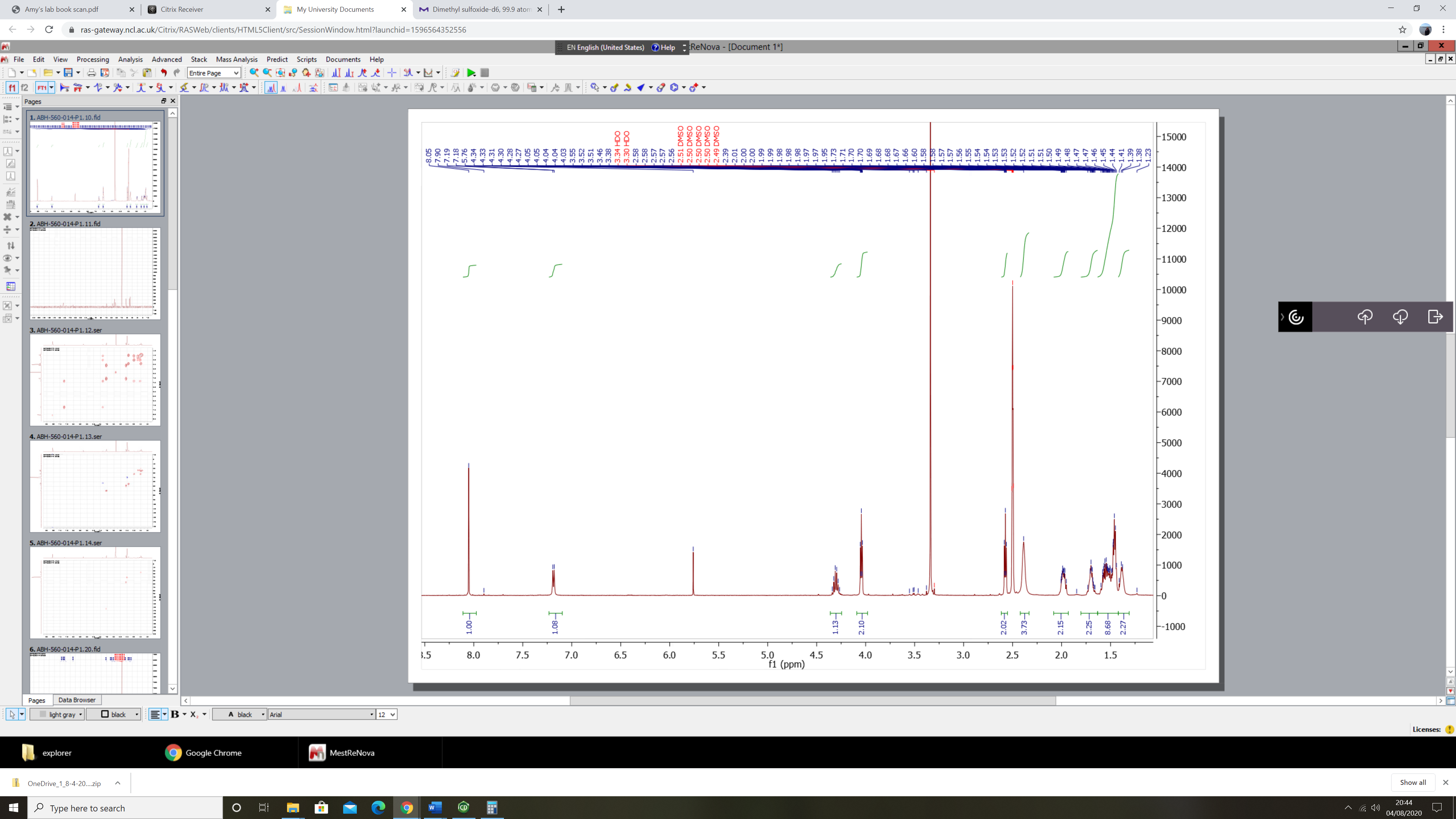


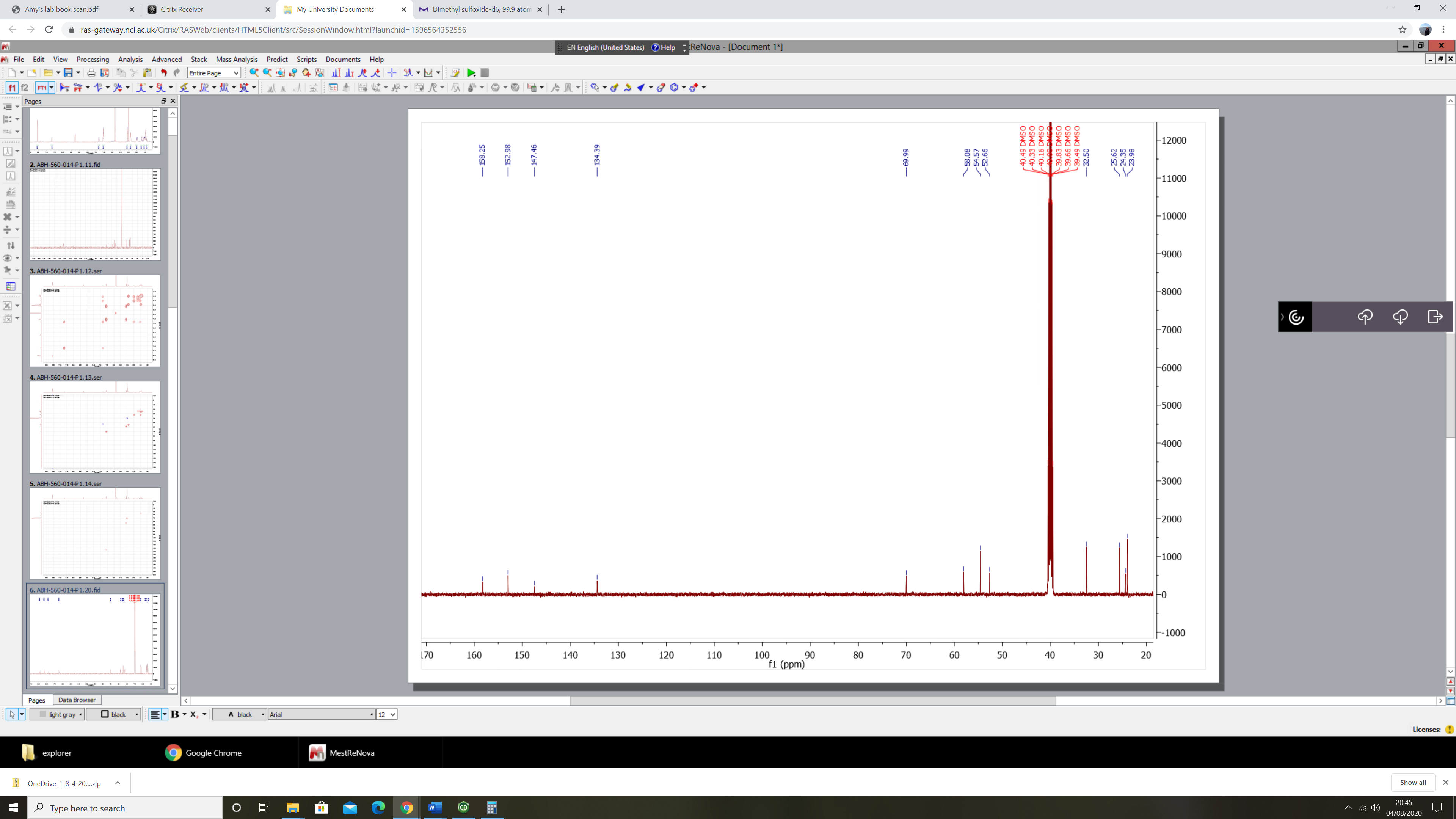


N-cyclopentyl-6-(piperidin-1-yl)-5-(vinyloxy)pyrimidin-4-amine **28**


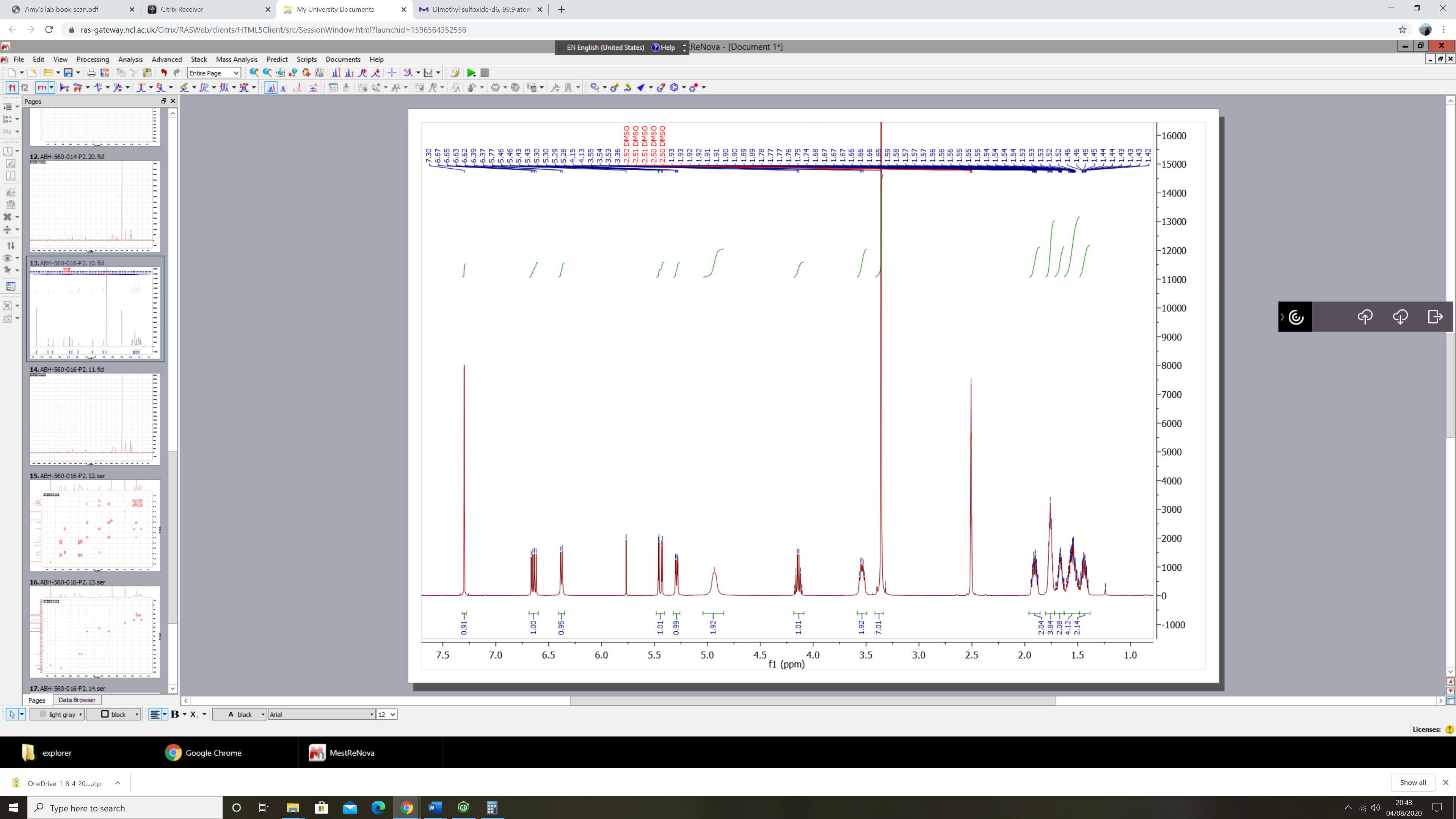


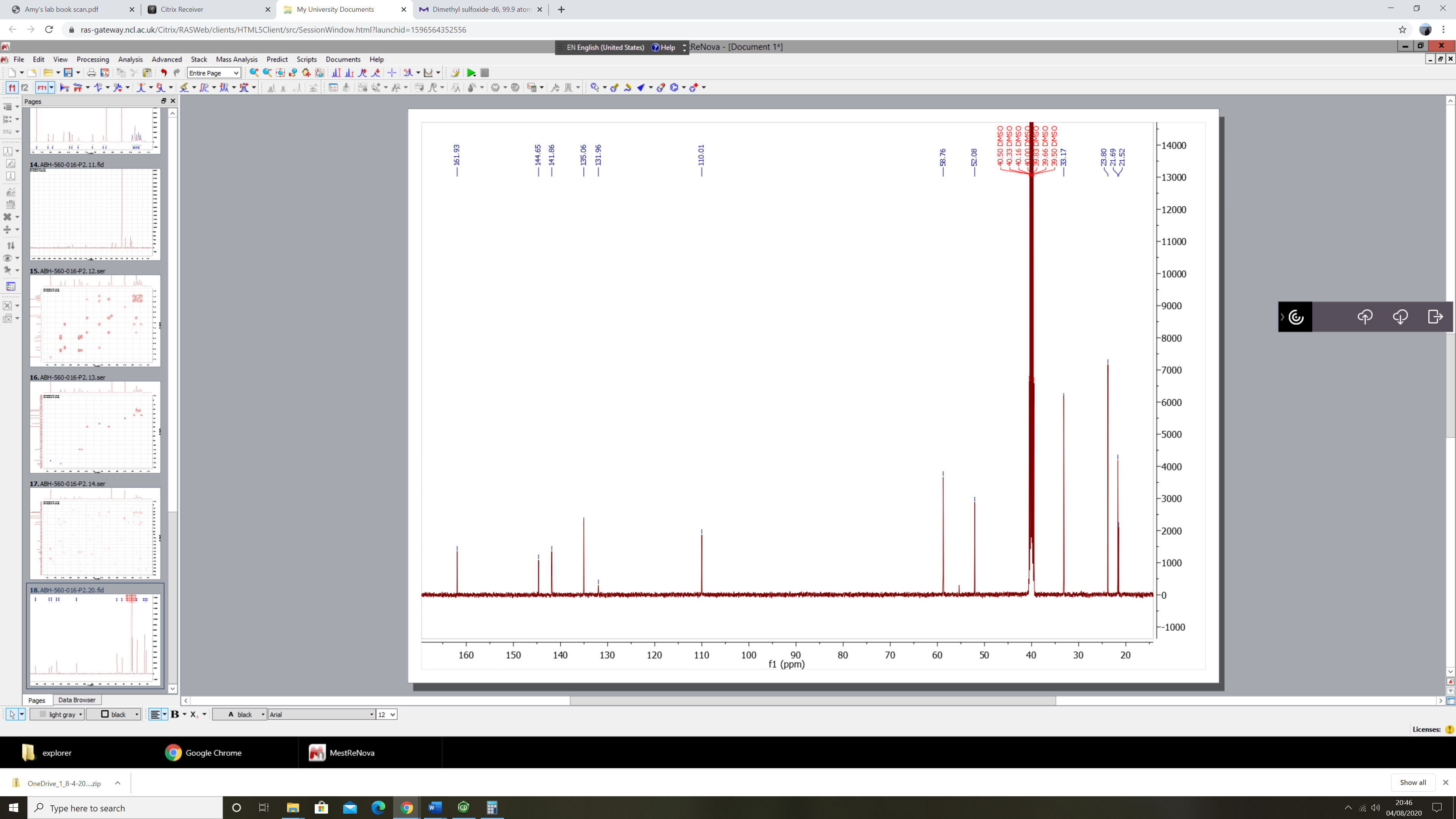

Supplement: Supplementary file 1 — Appendix S1. Supporting information [file JHET-58-947-s001.docx]
